# Supplementary material for: A critical realist analysis of nursing educators’ willingness to learn and teach patient safety in Sri Lanka: Study protocol
Source: PLoS One. 2025 May 19;20(5):e0323561. doi: 10.1371/journal.pone.0323561 (PMC12088512; doi:10.1371/journal.pone.0323561)
Supplement: S1 File — (DOCX) [file pone.0323561.s001.docx]

**Introduction**

Interview will be conducted in Sinhala, Tamil or English language as pre-determined by the interviewee.

| **Key** **Components:**  • Thank you  • Your name  • Purpose  • Confidentiality • Duration  • How interview will be conducted  • Opportunity for questions  • Signature of consent | I want to thank you for taking the time to meet with me today. My name is ____________________________ and I would like to know about your perceptions on patient safety education for nursing, in Sri Lanka and about your thoughts on adopting an innovative idea or a new educational innovation.  Specifically, I will be using your input to analyze how nursing educators’ individual perceptions on patient safety, characteristics of WHO Patient safety curriculum and environmental context contribute to their overall concerns on Patient safety education.  The interview should take about an hour. I will be recording the session because I don’t want to miss any of your comments. Although I will be taking some notes during the session, I can’t possibly write fast enough to get it all down. Because we’re recording, please be sure to speak up so that we don’t miss your comments.  Please do not introduce/ identify yourself or name third parties during the interview.  All responses will be kept confidential. This means that your interview responses will only be shared with research team members and we will ensure that any information we include in our report does not identify you as the respondent.  These recordings and transcripts will be stored in password protected files in an external storage device for 10 years.  You will be given an opportunity to review the transcription and provide consent to use this interview in the study.  Please note that, you don’t have to talk about anything you don’t want to. You may also ask to take a break or end the interview at any time.  Are there any questions about what I have just explained?  Are you willing to participate in this interview?  __________________ __________  Interviewee Date |
| --- | --- |

Adapted from: Boyce, Carolyn, and Palena Neale. Conducting in-depth interviews: A guide for designing and conducting in-depth interviews for evaluation input. Vol. 2. Watertown, MA: Pathfinder international, 2006

**Basic Interview protocol**

Individuals and perceptions/ innovation (PA)/ environmental context

**Part I- Patient safety education**

**Patient safety in hospitals (Environmental context)**

1. Have you ever encountered or witnessed any patient safety incidences during your training or service that you can still remember?
2. Can you explain your opinion on patient safety in Sri Lankan hospitals? Do you think we are giving enough attention to patient safety? Are there more important things that we should pay attention? Please explain.
3. According to your understanding, what are the contributing factors for patient safety issues in Sri Lanka?

**Individual innovativeness- patient safety (individual)**

1. Are you aware of the new patient safety curriculum of WHO?
   - If so, what is your opinion on it? Good/bad/ not practical/ worth giving a try?
2. What kind of training have you received on patient safety?
3. Whom would you contact if you would like to explore more on PS education? Any person or institution?
4. What are your thoughts on integrating patient safety education in to nursing curriculum? Should it be a separate subject or dispersed in other subjects? Please explain your idea.
5. What is your perception on new patient safety concepts mentioned below?

**CONCEPTS:** Adverse events in healthcare, Adverse events reporting, Human factors in healthcare safety, System factors in healthcare safety, Root cause analysis

- - Are they relevant to SL context? If no, please explain why
  - Do they cover any Practical issues at hospitals?
  - What are the potential barriers of integrating patient safety concepts at undergraduate level?
  - What are the benefits of teaching patient safety concepts at undergraduate level?

**Perceived attributes on new patient safety concepts- innovation (PA)**

CONCEPTS: Adverse events in healthcare, Adverse events reporting, Human factors in patient safety, System factors in patient safety, Root cause analysis

1. **Relative advantage –regarding responding to error/adverse events**
2. According to your understanding what is more effective to ensure patient safety; “naming, shaming, blaming” or learning from error?

1. **Compatibility**
2. Please explain whether the concepts related to learning from error (human factors, error reporting etc) compatible with

- your personal beliefs and values?
- values of the Sri Lankan society?
- Sri Lankan health care context?

1. **Complexity**
2. Are the concepts related to learning from error clear to you? Please describe any unclear areas?
3. Do you think the concepts related to learning from error are easy to teach or complicated? Please explain your opinion.
4. **Trialability**
5. Do you think that the concepts related to learning from error can be taught without requiring an extensive cultural adaptation? or does it need to be trialed to check suitability in Sri Lankan context? If so, how do you think that can be done?
6. **Observability**
7. Have you found any evidence from literature regarding the impact of learning from error on clinical practice? Have you found any best practices?

**Part II-Innovativeness**

**General innovativeness -individual**

1. Could you please explain how much you consider yourself to be open to change or willing to try new things?
2. How do you explain your risk taking behavior? E.g Taking Financial risk, changing career

**Individual innovativeness- nursing education -individual**

1. Please explain your willingness to adopt new concepts regarding teaching. Or do you like to hold to old methods, in terms of nursing curricula?
2. What factors do you think that made you to take that stance? Past experience? Education? Or any other reason?
3. Explain How do you keep your knowledge up-to-date on nursing curricular content?
4. Describe the nature support you get from hospitals to improve your teaching or to update your knowledge.
5. What is your opinion on the tendency and opportunities available for knowledge sharing between nurses in hospital and nursing educators? Prompt- are there established communication links? (Jang 2017)

**Institutional support- Environmental context**

1. Please explain if the nature of your work and structure of your work place facilitate you to be innovative? Or to try new things?
2. Can you please elaborate whether you get enough opportunities and resources to try new things at work? Or do you have to strictly repeat the established patterns of things?
3. Explain how you get information about new developments in Sri Lankan healthcare system. Are there established functioning channels of communication with MOH/ hospitals/ medical faculty?

**Socio- economic factors –Environmental context**

1. What socio-economical and individual factors that affect a person’s willingness to change or adopt a change in Sri Lanka?
2. What is your opinion on personal responsibilities affecting a person’s willingness to adopt changes, renew or improve things at work? Or innovativeness (skills and imagination to create new things) in general? Personal responsibilities such as:
   - Elderly parents/grandparents above 65years (with/without any mental or physical illness)
   - Children aged 10yrs and below
   - Family members who needs special attention (debilitating mental or physical illness/differently abled)
