# Supplementary material for: A critical realist analysis of nursing educators’ willingness to learn and teach patient safety in Sri Lanka: Study protocol
Source: PLoS One. 2025 May 19;20(5):e0323561. doi: 10.1371/journal.pone.0323561 (PMC12088512; doi:10.1371/journal.pone.0323561)
Supplement: S2 File — (DOCX) [file pone.0323561.s002.docx]

**Data extraction form- curricular content search**

University-

Faculty-

Department-

|  | **AER-PS topics stated in WHO-MPSC** | **Curricular components which state the concept** | **Subject/ module/ unit** | **Credits/ hrs** | **Teaching method** | **Inclusion of WHO-MPSC topics** | | |
| --- | --- | --- | --- | --- | --- | --- | --- | --- |
|  |  |  |  |  |  | Not mentioned | Partially mentioned | Explicitly mentioned |
|  | **1.What are adverse events in healthcare?** |  |  |  |  |  |  |  |
| 1 | the terms error, slip, lapse, mistake, violation, near miss and hindsight bias |  |  |  |  |  |  |  |
| 2 | the difference between system failures, violations and errors; |  |  |  |  |  |  |  |
| 3 | lessons about error and system failure from other industries; |  |  |  |  |  |  |  |
|  |  |  |  |  |  |  |  |  |
|  | **2.Adverse events reporting** |  |  |  |  |  |  |  |
| 4 | incident reporting |  |  |  |  |  |  |  |
| 5 | **additional:** introduction to incident reporting form (H 1259) in Sri Lanka |  |  |  |  |  |  |  |
|  |  |  |  |  |  |  |  |  |
|  | **3.Human factors in healthcare safety** |  |  |  |  |  |  |  |
| 6 | the meaning of the term human factors |  |  |  |  |  |  |  |
| 7 | the relationship between human factors and patient safety. |  |  |  |  |  |  |  |
|  |  |  |  |  |  |  |  |  |
|  |  |  |  |  |  |  |  |  |
|  | **4.System factors in healthcare safety** |  |  |  |  |  |  |  |
| 8 | the terms system and complex system as they relate to health care |  |  |  |  |  |  |  |
| 9 | why a systems approach to patient safety is superior to the traditional approach |  |  |  |  |  |  |  |
|  |  |  |  |  |  |  |  |  |
|  |  |  |  |  |  |  |  |  |
|  | **5.Root cause analysis** |  |  |  |  |  |  |  |
| 10 | how students/ nurses can learn from errors. |  |  |  |  |  |  |  |
| 11 | Conducting RCA |  |  |  |  |  |  |  |
| 12 | incident monitoring |  |  |  |  |  |  |  |
|  |  |  |  |  |  |  |  |  |
|  | Total |  |  |  |  | T1 | T2 | T3 |
|  |  |  |  |  |  |  |  |  |
|  | Percentages= T1 or T2 or T3/12 x100 |  |  |  |  | % | % | % |
