# Supplementary material for: A critical realist analysis of nursing educators’ willingness to learn and teach patient safety in Sri Lanka: Study protocol
Source: PLoS One. 2025 May 19;20(5):e0323561. doi: 10.1371/journal.pone.0323561 (PMC12088512; doi:10.1371/journal.pone.0323561)
Supplement: S3 File — (DOCX) [file pone.0323561.s003.docx]

**Individual Innovativeness Scale Score calculation**

Step 1: Add the scores for items 4, 6, 7, 10, 13, 15, 17, and 20.

Step 2: Add the scores for items 1, 2, 3, 5, 8, 9, 11, 12, 14, 16, 18, and 19.

Step 3: Complete the following formula:

Individual Innovativeness = 42 + total score for Step 2 - total score for Step 1.

**Interpretation of scores**

Scores above 80 are classified as Innovators.

Scores between 69 and 80 are classified as Early Adopters.

Scores between 57 and 68 are classified as Early Majority.

Scores between 46 and 56 are classified as Late Majority.

Scores below 46 are classified as Laggards/Traditionalists.

In general people who score above 68 and considered highly innovative, and

people who score below 64 are considered low in innovativeness
