# Supplementary figures and images for: A critical realist analysis of nursing educators’ willingness to learn and teach patient safety in Sri Lanka: Study protocol
Source: PLoS One. 2025 May 19;20(5):e0323561. doi: 10.1371/journal.pone.0323561 (PMC12088512; doi:10.1371/journal.pone.0323561)

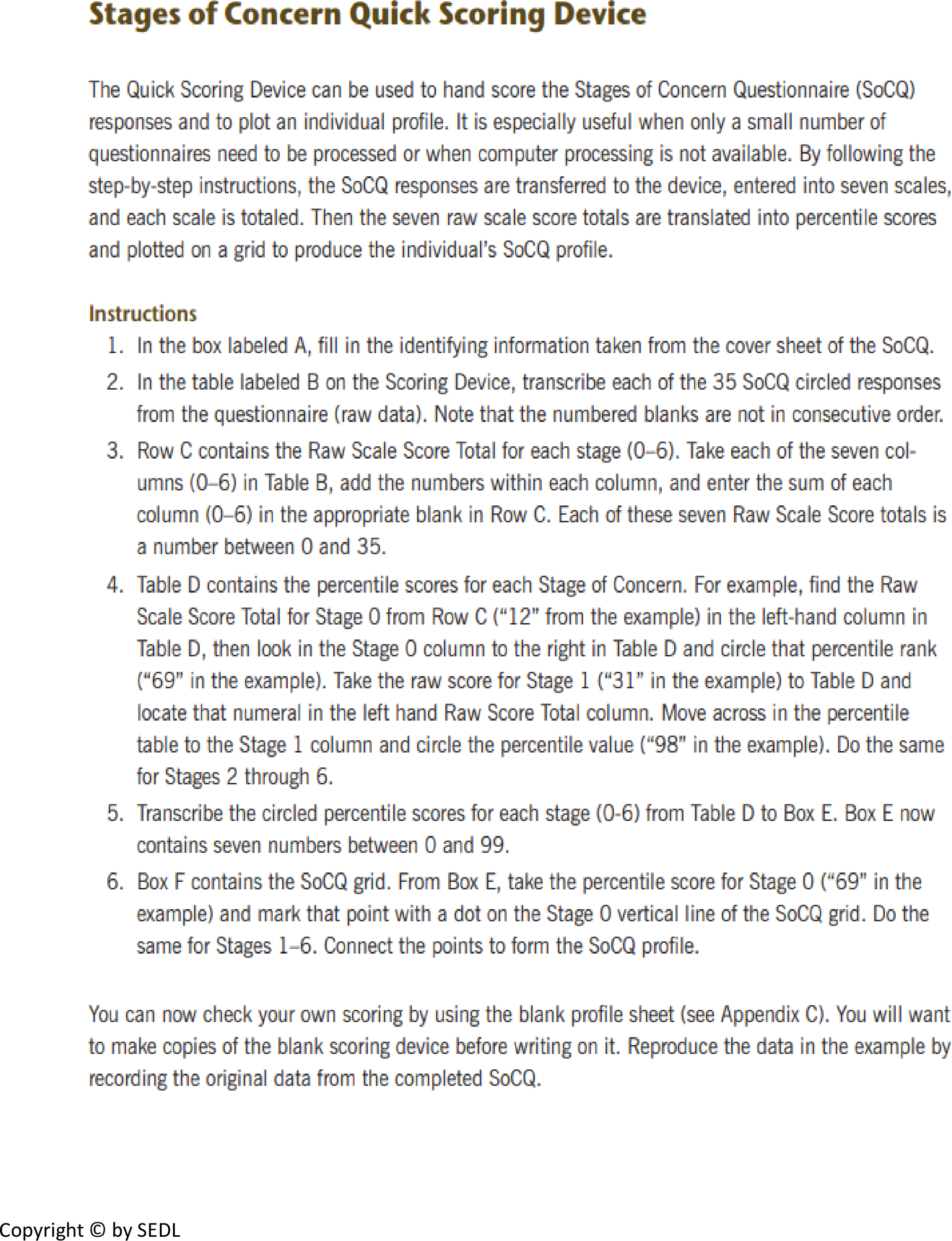

Supplement: S1 Fig — (TIF) [file pone.0323561.s004.tif]
