# Supplementary material for: A critical realist analysis of nursing educators’ willingness to learn and teach patient safety in Sri Lanka: Study protocol
Source: PLoS One. 2025 May 19;20(5):e0323561. doi: 10.1371/journal.pone.0323561 (PMC12088512; doi:10.1371/journal.pone.0323561)
Supplement: Attachment — Submitted filename: PONE Review LG 07_03_2025.pdf [file pone.0323561.s010.pdf]

PLOS ONE

A critical realist analysis of nursing educators’ willingness to learn and teach patient safety in Sri Lanka: Study protocol

--Manuscript Draft--

**Manuscript Number:**	PONE-D-23-13398R3
**Article Type:**	Study Protocol
**Full Title:**	A critical realist analysis of nursing educators’ willingness to learn and teach patient safety in Sri Lanka: Study protocol
**Short Title:**	Analysis of nursing educators’ willingness to learn and teach patient safety
**Corresponding Author:**	Ashoka Dissanayake<br>University of Sri Jayewardenepura<br>Nugegoda, Sri Lanka SRI LANKA
**Keywords:**	Curriculum; Nursing Education; Patient safety; qualitative research; Teaching; Validation Study
**Abstract:**	Introduction:<br>Nursing educators' willingness to learn and teach patient safety is identified as a challenge in the process of implementing patient safety education in nursing programmes. However, the literature lacks an in-depth analysis of the factors affecting their willingness. This study aims to identify factors affecting willingness of nursing educators to learn and teach new patient safety concepts.<br>Methods:<br>The study setting will be the Diploma and B.Sc. Nursing programs conducted in eight state universities, two private universities approved by the University Grants Commission, and 16 Nurses Training Schools (NTS) affiliated with general and teaching hospitals under the Ministry of Health, Sri Lanka. The sample will consist of permanent nursing educators who are fluent in Sinhala language.<br>The study design adopts the Critical Realist Mixed Method Sequential Explanatory Design (CRMMSED), based on the critical realistic staged model for explanatory social sciences and Mixed Method Sequential Explanatory design (MMSE). Post conceptualization, the execution of the study will take place in four phases; 1. Extensive data collection and analysis, 2. Linking Phase, 3. Intensive data collection and analysis and 4. Interpretation phase. In Phase 1 , curriculum content verification , cross-cultural adaptation and validation of the Sinhala version of the Individual Innovativeness scale, data collection using the questionnaire, identification of educators' stages of concern and Individual innovativeness will occur. During the linking phase interviewees will be selected based on quantitative data analysis. In phase 3, qualitative data will be collected through semi-structured interviews with nursing educators and data will be analysed using deductive thematic analysis. In the interpretation phase, quantitative and qualitative data analyses will be triangulated to understand structures and mechanisms contributing to educators' willingness.<br><br>Data analysis:<br>SPSS and AMOS will be used to conduct exploratory and confirmatory factor analysis of the questionnaire. Furthermore, associations and correlations of quantitative data will be identified. NVivo will be utilized for processing and thematic analysis of qualitative data. Retroductive inference and causative analysis will identify underlying events, structures, and generative mechanisms.
**Order of Authors:**	Dissanayake Mudiyanselage Ashoka Priyadarshani Dissanayake
	Sudath Shirley Pathmasiri Warnakulasuriya, Ph.D.
**Response to Reviewers:**	Reviewer #4:<br>Thanks for your valuable article. it seems that the needs for conducting this research should be more explained in the introduction.Thanks for your encouraging feedback. We have expanded “significance of the study”. L298-309<br><br>Reviewer #5:<br>Abstract - It is clearer if authors mention "Methods" after introduction instead of setting

and design. Under methods, you could describe all the components (setting, study population, inclusion and exclusion criteria, sample size, sampling method, study instruments....). Thank you for your suggestion. Changed the subtopics. We did not elaborate on the sampling method, and sampling size in the abstract due to the word limit. But these have been explicitly mentioned in the body of the article.

Under data analysis, what are the statistical tests utilized for analysis?Factor analysis- EFA and CFA, associations and correlations of questionnaire data were included in the abstract. L42-44

The study protocol is a lengthy one. contain a validation, audit and descriptive studies which involve quantitative and qualitative parts. Better if authors can clearly mention as stages or phases under the whole study.This information is provided in Table 2. Comparison of study designs and in Abstract. L30-32
L363

There are some repetitions which could be avoided and leads to a concise protocol.We could avoid some repetitions if we rearrange the manuscript according to the Phases (collating sampling, instruments, data collection and analysis together), But then it will deviate from the manuscript guideline of the journal. Nevertheless, minor changes were done to address this.

As this is not an analytical study, how the authors assess the hypothesis mentioned?Thank you for highlighting this. We agree to your opinion that this study does not necessarily need hypotheses to execute it or for its' analysis. removed hypotheses.

In hypothesis 1 - line 341 - repetition of " nursing educators"removed.

Specific objectives 6 and 7 are more or less same.
6. To examine the perceived attributes and environmental factors that facilitate or hinder the adoption of adverse events related patient safety concepts by nursing educators.
7. To identify the events that contribute to the facilitation or hindrance of the adoption of adverse events related patient safety concepts.

Perceived attributes and environmental factors are in the empirical domain.
Phenomena in empirical domain are observed and experienced.
Perceived attributes on an innovative concept cannot be equated to actual events.
We accept that certain "opportunities" participants mention have the possibility of being identified as actual events. To overcome this: if a participant mentions certain opportunity (eg. training) was helpful in accepting new PS concepts, we will use this finding in empirical domain. We will further inquire to find out what directives/policies or recommendations were there enabling these opportunities. Latter findings will be identified as Actual Events.

7. To identify the events that contribute to the facilitation or hindrance of the adoption of adverse events related patient safety concepts.
8. To analyse how variations in willingness to learn and teach PS emerge from the underlying generative mechanisms and structures

To improve clarity we amalgamated the objective on "actual domain" with "real domain" as both objectives are operationalized using causal analysis, retroduction and "synthesis".
7. To synthesis the actual events, generative mechanisms and structures that contribute to the facilitation or hindrance of the adoption of adverse events related patient safety concepts.

Specific objective 8 - Is this realistic?In Critical realism, retroduction is the central mode of inference. Retroduction is the way to dive beyond the measurable elements of observations through events to identify the structures, context, and mechanisms that explain the observation.
Our belief in the existence of a mechanism can be based either on our ability to directly observe it (perceptual criteria), with or without tools to do so, or on our ability to

observe its effects (causal criteria) (Bhaskar 1975, p. 179).
Retroduction allows researchers to identify a set of plausible candidate causal mechanisms. This enables a researcher to investigate the potential causal mechanisms and the conditions under which certain outcomes will or will not be realised.
Empirical corroboration allows to selection of the mechanism(s) that offers the best explanation (Mukumbang, 2021).These mechanisms belong to different strata of reality: (i) physical, (ii) biological, (iii) psychological, (iv) psychosocial, socio-economic,(v) cultural, and (vii) normative mechanisms (Danermark, 2019).
Some examples of such analysis: Saxena, D. (2019), Wynn Jr, D. E., & Williams, C. K. (2008)8. To analyse how variations in willingness to learn and teach PS emerge from the underlying generative mechanisms and structures.

new 7. To synthesis the actual events, generative mechanisms and structures that contribute to the adoption of adverse events related patient safety concepts.

There is a possibility that those who are not willing to accept a new idea or new PS concepts in particular will not even be bothered to answer the questionnaire or participate in the study at all. Therefore, we will not be able to identify/synthesis what causes them to deny this new concept. This will be a limitation of this study and will be acknowledged in limitations. L682-684

Inclusion and exclusion criteria - Please mention clearly.Added in Study Design.L385-394

Qualitative component - How do you know sample size of 12 - 14 will come to the saturation point?We agree that it has to be changed to "semi-structured interviews will be conducted with nursing educators until data saturation is reached".L443-444

The sampling method should be more precise and practical for each study component. Eg. How you are going to recruit 105 from each institution mentioned/ how many from each/ sub sampling mechanisms.....Included subsampling methods in sample calculation L410L 388- all available curricula in selected institutions will be included in content verification using a purposive sampling technique
L 396-The participants will be selected from three types of institutions (state universities, private universities, and training schools) using stratified random sampling.
L400- participants who were selected using stratified random sampling.
L431- Participants will be selected purposive sampling method.

Validation is planned to be done on Hurt-Joseph-Cook individual innovativeness questionnaire. Pls clearly mention what is the validating procedure? Is it construct validity? If so is it factor analysis? EFA? CFA? Both? Then what are the steps? Is it PCA? ....
EFA and CFA. The steps of validating procedure is mentioned in "Data analysis" Phase 1. L552- L556. Extraction Method: Principal Component Analysis.
Rotation Method: Varimax with Kaiser Normalization.

But SoC questionnaire is already validated in Sri Lanka with good psychometric properties. Then why you are going to perform CFA on it?It is an attempt follow a good practice since wording of the questionnaire was changed to suit patient safety education context. "Subsequent validation" is suggested by Tsang et al. (2017)

How to ensure the validity and reliability of self-administered questionnaire which will be used in study in addition to two standard questionnaires?The self-administered questionnaire has 3 parts.
Part1- Demographics- standard questions are being used.
Part2-SoC questionnaire- cronbatch alpha, subsequent validation CFA
Part3-II questionnaire- cronbatch alpha, validation using EFA and CFA

As mentioned in the supporting materials, scoring of the Individual Innovation scale, > 68 = high; < 64 = low. Then what about the score between 64 - 68? Thank you for pointing this out. We accept that this information is incomplete. Unfortunately, there is no description for the score between 68-64 in the scale. We would like to remove this

Powered by Editorial Manager® and ProduXion Manager® from Aries Systems Corporation

	part as the scoring above this description provides more clarity on innovativeness categories.<br><br>Anonymity (which is fundamental in obtaining views clearly and successful manner) could be secured if you are going to use a coding system.Thank you for your valuble suggestion. It is mentioned in the protocol in "Data Collection"L481-482 Data collection is not anonymised. In order to protect privacy and confidentiality; names and university affiliations will be replaced with codes during data processing.<br><br>References<br>Danermark B, Ekström M, Karlsson JC. Explaining society: Critical realism in the social sciences. Routledge; 2019 Mar 20.<br>Saxena D. Guidelines for conducting a critical realist case study. International Journal of Adult Education and Technology (IJAET). 2021 Apr 1;12(2):18-30.<br>Tsang S, Royse CF, Terkawi AS. Guidelines for developing, translating, and validating a questionnaire in perioperative and pain medicine. Saudi journal of anaesthesia. 2017 May 1;11(Suppl 1):S80-9.<br>Wynn Jr DE, Williams CK. Critical realm-based explanatory case study research in information systems.
**Additional Information:**	
**Question**	**Response**
**Financial Disclosure**<br><br>Enter a financial disclosure statement that describes the sources of funding for the work included in this submission. Review the submission guidelines for detailed requirements. View published research articles from *PLOS ONE* for specific examples.<br><br>This statement is required for submission and **will appear in the published article** if the submission is accepted. Please make sure it is accurate.	DMAP received a research grant from University Grants Commission- Sri Lanka, UGC/VC/DRIC/PG2018(II)/SJP/01.<br>https://www.ugc.ac.lk/index.php?option=com_content&view=article&id=2348&Itemid=134&lang=en<br><br>The funders did not and will not have a role in study design, data collection and analysis, decision to publish, or preparation of the manuscript.

**Unfunded studies**<br>Enter: *The author(s) received no specific funding for this work.*<br><br>**Funded studies**<br>Enter a statement with the following details:<br>• Initials of the authors who received each award<br>• Grant numbers awarded to each author<br>• The full name of each funder<br>• URL of each funder website<br>• Have or will the sponsors or funders play any role in the study design, data collection (when applicable) and analysis, decision to publish, or preparation of the manuscript?<br>• **NO** - Include this sentence at the end of your statement: *The funders did not and will not have a role in study design, data collection and analysis, decision to publish, or preparation of the manuscript.*<br>• **YES** - Specify the role(s) played.<br><br>* typeset	
**Competing Interests**<br><br>Use the instructions below to enter a competing interest statement for this submission. On behalf of all authors, disclose any competing interests that could be perceived to bias this work—acknowledging all financial support and any other relevant financial or non-financial competing interests.<br><br>This statement is required for submission and **will appear in the published article** if the submission is accepted. Please make sure it is accurate and that any funding sources listed in your Funding Information later in the submission form are also declared in your Financial Disclosure statement.<br><br>View published research articles from *PLOS ONE* for specific examples.	The authors have declared that no competing interests exist.

**NO authors have competing interests**<br><br>Enter: *The authors have declared that no competing interests exist.*<br><br>**Authors with competing interests**<br><br>Enter competing interest details beginning with this statement:<br><br>*I have read the journal's policy and the authors of this manuscript have the following competing interests: [insert competing interests here]*<br><br>* typeset	
**Ethics Statement**<br><br>Enter an ethics statement for this submission. This statement is required if the study involved:<br><br>• Human participants<br>• Human specimens or tissue<br>• Vertebrate animals or cephalopods<br>• Vertebrate embryos or tissues<br>• Field research<br><br>Write "N/A" if the submission does not require an ethics statement.<br><br>General guidance is provided below. Consult the submission guidelines for detailed instructions. **Make sure that all information entered here is included in the Methods section of the manuscript.**	Ethical clearance to conduct this study has been obtained by the Ethical Review Committee at Faculty of Graduate Studies, University of Colombo (FGS/ERC/2022/013).<br>Informed written consent will be obtained from all the participants for the study. All methods will be carried out in accordance with declaration of Helsinki.

Powered by Editorial Manager® and ProduXion Manager® from Aries Systems Corporation

**Format for specific study types**<br><br>**Human Subject Research (involving human participants and/or tissue)**<br>• Give the name of the institutional review board or ethics committee that approved the study<br>• Include the approval number and/or a statement indicating approval of this research<br>• Indicate the form of consent obtained (written/oral) or the reason that consent was not obtained (e.g. the data were analyzed anonymously)<br><br>**Animal Research (involving vertebrate animals, embryos or tissues)**<br>• Provide the name of the Institutional Animal Care and Use Committee (IACUC) or other relevant ethics board that reviewed the study protocol, and indicate whether they approved this research or granted a formal waiver of ethical approval<br>• Include an approval number if one was obtained<br>• If the study involved *non-human primates*, add *additional details* about animal welfare and steps taken to ameliorate suffering<br>• If anesthesia, euthanasia, or any kind of animal sacrifice is part of the study, include briefly which substances and/or methods were applied<br><br>**Field Research**<br><br>Include the following details if this study involves the collection of plant, animal, or other materials from a natural setting:<br>• Field permit number<br>• Name of the institution or relevant body that granted permission	
**Data Availability**<br><br>Authors are required to make the data underlying their research findings fully available, without restriction.<br><br>If the manuscript reports pilot or preliminary results, the data underlying those results must be made available at the time of publication. A Data Availability	N/A - No results are reported

Powered by Editorial Manager® and ProduXion Manager® from Aries Systems Corporation

Statement describing where the data can be found is required at submission. Your answers to this question constitute the Data Availability Statement and will be published in the article, if accepted. When legal or ethical requirements prohibit public data sharing, authors must indicate how researchers may obtain access to the data. See the PLOS Data Policy and FAQ for detailed information.<br><br>**Important:** Stating 'data available on request from the author' is not sufficient. If your data are only available upon request, select 'No' for the first question and explain your situation in the text box.<br><br>For protocols without pilot or preliminary data, authors are strongly encouraged to state how they plan to share research data from their study when it is completed or published.<br><br>If your study includes pilot data, will they be made fully available without restriction?	
**Describe where the data may be found in full sentences. If you are copying our sample text, replace any instances of XXX with the appropriate details.**<br><br>• If **no pilot data are reported** in the manuscript, please enter the following: *No datasets were generated or analysed during the current study. All relevant data from this study will be made available upon study completion.*<br>• If the manuscript reports pilot data **held in a public repository**, include URLs, accession numbers or DOIs. If this information will only be available after acceptance, indicate this by ticking the box below. For example: *All XXX files are available from the XXX database (accession number(s) XXX, XXX.).*<br>• If the manuscript reports pilot data contained **within the manuscript and/or**	Deidentified research data will be made publicly available when the study is completed and published.

**Supporting Information files**, enter the following:<br>*All relevant data are within the manuscript and its Supporting Information files.*<br>• If neither of these applies but you are able to provide **details of access elsewhere**, with or without limitations, please do so. For example:<br><br>*Data cannot be shared publicly because of [XXX]. Data are available from the XXX Institutional Data Access / Ethics Committee (contact via XXX) for researchers who meet the criteria for access to confidential data.*<br><br>*The data underlying the results presented in the study are available from (include the name of the third party and contact information or URL).*<br>• This text is appropriate if the data are owned by a third party and authors do not have permission to share the data.<br><br>For study protocols with data management or sharing plans, authors are encouraged to briefly describe in their Data Availability Statement how data they generate will be made accessible when the study is completed. For example:<br>• Deidentified research data will be made publicly available when the study is completed and published.<br><br>* typeset	
Additional data availability information:	Tick here if the URLs/accession numbers/DOIs will be available only after acceptance of the manuscript for publication so that we can ensure their inclusion before publication.

Manuscript

Click here to access/download;Manuscript;Revised manuscript R3.docx

A critical realist analysis of nursing educators' willingness to learn and teach patient safety in Sri Lanka: study protocol

Dissanayake, DMAP [1, 2] , Warnakulasuriya, SSP [3]

1. Dept. of Nursing and Midwifery, Faculty of Allied Health Sciences, University of Sri Jayewardenepura, Sri Lanka
2. Faculty of Graduate studies, University of Colombo, Sri Lanka.
3. Faculty of Nursing, University of Colombo, Sri Lanka

Competing interests- The authors declare that there are no competing interests.

Availability of data and materials –Data will be available upon request when the study is completed and published.

Abstrac[illegible]

Introduction:

Nursing educators' willingness to learn and teach patient safety is identified as a challenge in the process of implementing patient safety education in nursing programmes. However, the literature lacks an in-depth analysis of the factors affecting their willingness. This study aims to identify factors affecting willingness of nursing educators to learn and teach new patient safety concepts.

Methods:

The study setting will be the Diploma and B.Sc. Nursing programs conducted in eight state universities, two private universities approved by the University Grants Commission, and 16 Nurses Training Schools (NTS) affiliated with general and teaching hospitals under the Ministry of Health, Sri Lanka. The sample will consist of permanent nursing educators who are fluent in Sinhala language.

The study design adopts the Critical Realist Mixed Method Sequential Explanatory Design (CRMMSED), based on the critical realistic staged model for explanatory social sciences and Mixed Method Sequential Explanatory design (MMSE). Post conceptualization, the execution of the study will take place in four phases; 1. Extensive data collection and analysis, 2. Linking Phase, 3. Intensive data collection and analysis and 4. Interpretation phase. In Phase 1 , curriculum content verification , cross-cultural adaptation and validation of the Sinhala version of the Individual Innovativeness scale, data collection using the questionnaire, identification of educators' stages of concern and Individual innovativeness will occur. During the linking phase interviewees will be selected based on quantitative data analysis. In phase 3, qualitative data will be collected through semi-structured interviews with nursing educators and data will be analysed using deductive thematic analysis. In the interpretation phase, quantitative and qualitative data analyses will be triangulated to understand structures and mechanisms contributing to educators' willingness.

Data analysis:

SPSS and AMOS will be used to conduct exploratory and confirmatory factor analysis of the questionnaire. Furthermore, associations and correlations of quantitative data will be identified. NVivo will be utilized for processing and thematic analysis of qualitative data. Retroductive inference and causative analysis will identify underlying events, structures, and generative mechanisms.

What is already known about the topic?

- Nurse educators' willingness to learn and teach patient safety has been identified as a contributing factor to the success of integrating patient safety concepts into existing curricula.
- According to the Concerns Based Adoption Model (CBAM), educators' concerns about education innovation comprises seven categories. Stages are ranging from "unconcerned" to "informational", "personal", "management", "consequence" "collaboration" and "refocusing".
- Individual innovativeness is defined as the "willingness of an individual to change". Previous studies have found a positive relationship between individual innovativeness and education technology adoption behaviour.

What does this paper add?

- With this protocol, attempts will be made to identify individual, environmental, and innovation-related factors which contribute to the emergence of differentiations in willingness to learn and teach patient safety concepts among nursing educators.

- Using this protocol, it will be proposed to explore the possibility of synergising mixed methods research and critical realism, which enables the researcher to identify varied categories of the study population through quantitative data and to identify the causative mechanisms of those categories through qualitative data.

Key words: Curriculum; Nursing Education; Patient Safety; Qualitative Research; Teaching; Validation study

Introductio[illegible]

Patient safety (PS) has been identified as an emerging trend in health services, and the World Health Organization (WHO) advocates for its inclusion in education accreditation (1). According to the WHO, PS refers to a framework of organized activities that promote cultures, processes, procedures, behaviours, technologies, and environments in healthcare that consistently and sustainably lower risks, reduce the occurrence of avoidable harm, make errors less likely, and reduce their impact when they do occur (2). In the context of ensuring patient safety, the emphasis has been shifted from assigning individual blame to recognizing and addressing the impact of human factors and fallibility. It is essential that these new concepts are effectively transmitted to the next generation of healthcare professionals.

Education on PS has been identified as a proactive step towards safer care and there is a global agreement on PS in undergraduate healthcare education (3,4). Numerous PS frameworks have been developed and integrated into nursing programmes worldwide, such as the Quality and

Safety Education for Nurses (QSEN) in the USA (5), Australian National Patient Safety Education Framework (6), South Korean Patient Safety Competency Framework (PSCF) (7) and Learning Outcomes for Patient Safety in Undergraduate Nursing Curricula in Canada (8). WHO-Multi Professional Patient Safety Curriculum is also based on the Australian framework.

However, curriculum implementers have reported the challenging nature of integrating PS into curricula. Limited understanding of the PS concepts (9,10) and reducing PS practices to just one aspect, such as focusing solely on reducing medication errors (11), can hinder its overall impact. Furthermore, accommodating PS into an already saturated nursing curriculum (12,13) ,and not recognizing its importance in clinical practice (4,11,14) pose additional challenges.

One of the prominent challenges in integrating PS into nursing curricula is the willingness of faculty to learn and teach these concepts (4,15,16). Several other issues related to faculty, such as a lack of recognition of the importance of PS by the faculty and a lack of confidence in teaching these concepts, have also been identified as challenging (4,17,18) . Nursing educators must be willing to integrate and teach new PS concepts to produce nurses who can champion PS. Therefore, further research is needed to evaluate both the curricula and nurse educators' willingness to learn and teach these concepts.

Patient safety education in the South Asian region and Sri Lanka

A few studies have been conducted in the Southeast Asian region to assess the perceptions of PS, barriers to implementing PS and strategies to overcome these barriers. Pelzang et al.,(9) have

emphasized the need to integrate and provide PS training and education to all categories of healthcare professionals. The lack of resources is the main barrier to promoting patient safety and quality practices in India (19,20).

In 2016 adverse events reporting forms were introduced to state-sector hospitals in Sri Lanka. In 2019 out of 10324 adverse events reported 30.46% are falls (21). Even though non-clinical events are reported more, it can only be predicted that actual numbers are higher (21,22). A study done in a teaching hospital in Sri Lanka revealed that nursing officers who are competent in skills regard to adverse events and, near misses were 6.35% (23). Nearly half (46.4%) of student nurses have agreed that making errors is inevitable while a considerable proportion (38%) agreed on blaming and punishing people who commit errors (24). These findings emphasise the dire need for improving PS education for nurses in Sri Lanka, particularly in adverse event reporting.

The "WHO South East Asia Regional Strategy for patient safety (2016-2025)" has proposed for integrating PS principles and practices into all professional healthcare courses, including nursing (25). This implies that Sri Lanka has strategically agreed to incorporate PS education in undergraduate nursing education. These new perspectives and obligations make PS education an innovative and emerging necessity, particularly for a developing country like Sri Lanka.

World Health Organizations' Multi-Professional Patient Safety Curriculum (WHO-MPSC) (26) provides a common foundation for PS education. The curriculum contains a wide array of topics on patient safety, human factors, complex systems, teamwork, learning from error, clinical risk

management, quality improvements, engaging patients and carers, infection control, surgical safety and medication safety. "It contains information for all levels of faculty and staff, and it lays the foundation for capacity building in essential patient safety principles and concepts" (27). The guide has been written to be applicable to different cultures and contexts using easily-understood language (27). Ginsberg et al (12) have found that low and middle-income countries are slow to progress towards integrating the aforesaid curriculum. They have examined the potential barriers pertaining to; a) curriculum content, b) context and c) implementation process. Insufficient training to enable faculty, lack of faculty enthusiasm/meaningful participation and lack of faculty cooperation to address implementation challenges were among the top three potential barriers to the implementation process. Therefore, the concerns of nursing faculty should be identified and addressed to ensure seamless integration of PS concepts into existing curricula.

Factors that affect willingness to learn and teach a new concept

Individual factors

Stages of Concern

The Concerns-Based Adoption Model (CBAM) (28) describes how teachers' concerns over the acceptance and adoption of educational change vary. Seven Stages of Concern (SoC) about an education innovation have been identified in which individuals progressed from implementing innovation to becoming competent in using it (Table 1).

Table 1: Typical expressions of concern about an innovation (28)

**Stages of Concern**			**Expressions of Concern**
"Impact"	6	Refocusing	I have some ideas about something that would work even better.
	5	Collaboration	I would like to coordinate my efforts with others, to maximize the innovation's effect.
	4	Consequence	How is my use affecting my students?
"Task"	3	Personal	I seem to be spending all my time getting my materials ready.
"Self"	2	Management	How will using it affect me?
	1	Informational	I would like to know more about it.
"Unconcerned"	0	Unconcerned	I am not concerned about it.

A 35-item Stages of Concern questionnaire is available in order to determine where someone is in the Stages of Concern range (28). Although it may appear that progression through the stages of concern is a linear process, it is important to note that acceptance and adoption of an innovation are highly influenced by the intrinsic factors of an individual. Even as individuals gain more knowledge and experience with innovation, there is no guarantee that their earlier concerns will be resolved (28).

The authors of "Measuring implementation in schools: the stages of concern questionnaire", George et al (28) noted that the development of higher-level concerns depends on the **individual's perceptions**, **the environmental context** and **the innovation** itself. Buabeng-Andoh

(29) identified personal, institutional, and technological factors that encourage the use of computer technology in teaching and learning, such as ICT competence, computer self-efficacy, professional development, and technical support. Lai and Chen (30) also suggested similar factors, including individual characteristics such as perceived usefulness and ease of use, school characteristics such as incentives and support, and environmental characteristics such as supervisor and peer influence.

Individual innovativeness

When introducing an innovative idea, the process of adapting that idea is rarely linear (31). Roger's diffusion of innovation theory explains that an individual's adoption of an innovation progresses through five stages: knowledge, persuasion, decision, implementation, and confirmation (31). Additionally, he suggests that individuals will adopt innovations at different time frames depending on the adopter category to which they belong (Innovators, Early Adopters, Early Majority, Late Majority, and Laggards) (31).

The general innovativeness of an individual is typically viewed as a psychological construct or individual characteristic. It shapes an individual’s disposition toward newness regardless of the kind of innovation (32). Hurt et al., (33) define Individual Innovativeness as the “willingness of an individual to change”. Previous studies have found a positive relationship between individual innovativeness and education technology adoption behaviour among academicians and education administrators (34,35).

Apart from Individual innovativeness, there are studies done to identify how personal characteristics such as age, gender, and education affect innovation adoption (36–38).

Environmental factors

Nursing educators working environment includes the teaching facility and the hospitals where clinical teaching and practical exams are carried out. Previous studies in nursing have frequently utilized (39–41) Kanter's 'Theory of Structural Power in Organizations' (42) to examine structural empowerment. Structural empowerment refers to workplace conditions and policies that facilitate four dimensions. These dimensions are access to information, support from colleagues, resources such as time and technology, and opportunities for professional development and growth. These dimensions influence the nursing educators' work environment and their ability to provide high-quality education to students. Research done in the management and IT industries has found that positive working environments such as strong managerial support, and allocation of sufficient resources and incentives become crucial catalysts in embracing innovation (38,43). As such, it could be predicted that nursing educators' environment could affect education innovation adoptions.

Factors related to the innovation

Perceived attributes (PA) are important factors to consider when studying the adoption of an innovation. Relative advantage, compatibility, complexity, observability, and trialability are the five main perceived attributes (31). Relative advantage means how much better an innovation is perceived to be, compared to the idea it replaces. Compatibility refers to how well the innovation fits with the values, experiences, and needs of potential adopters. Complexity explains how difficult the innovation is to understand and use. Observability refers to how visible the results

of the innovation are to others. Trialability refers to the ability of individuals to experiment with the innovation before fully adopting it. These perceived attributes have been used in previous research to investigate their effect on the concern of users when adopting an innovation (29,30).

The methodological approach

The methodology is concerned with the theoretical and philosophical assumptions about methods (44). It provides the frame of reference for the research which is influenced by the paradigm in which our theoretical perspective is placed or developed (45). Another definition is “the specific theoretical level at which research methods and approaches are conceived, chosen and justified” (46). Choosing a set of methods as opposed to others can be called methodological justification of the choice of methods (46). Methodology can be objectivist, subjectivist or critical realist.

Objectivist methodology

Objectivist methodology is based on positivism. It claims that reality exists independent of the human mind, and it can be understood only through objective methods of inquiry (47). Objective research methods require quantifiable data about the world to represent reality. When used in social research, objectivist methodology faces debilitating limitations. It may oversimplify complex social phenomena by reducing them to quantifiable variables, potentially overlooking important nuances and contextual factors. This approach may not capture the full richness and complexity of human experiences and social interactions (46).

Subjectivist methodology

Subjectivist methodology is based on interpretivism claims that the social and human world is a human creation based on their subjective encounters with them. There is no objective reality. It emphasizes that research should aim at understanding how people make sense of their own world (46).The limitations of this methodology include relying heavily on individual interpretations, which are influenced by personal biases or social contexts (47). The subjectivist methodology may not provide a comprehensive understanding of complex phenomena as it primarily focuses on individual perspectives and experiences, potentially overlooking broader structural or systemic factors (46).

Critical realistic methodology

Critical realistic methodology tries to transcend the subjectivist and objectivist dichotomy in epistemology and methodology. The triumvirate of critical realism is ontological realism, epistemic relativism and judgemental rationality (48).

Ontological realism

CR posits that reality is intransitive. There is an objective reality in the social world outside the human mind regardless of our ability to understand or decode it. Social reality includes languages, social processes, classes, economies, religion, whereas natural reality includes plants, trees, gravity etc. (48,49).

Epistemic relativism

Human knowledge gets limited by the methods we would use to uncover reality. Therefore, human knowledge is transitive. Our knowledge is finite, contextual and fallible (48,50,51).

Judgemental rationality

Critical realists should evaluate diverse and competing claims about the world before making a judgement. Human knowledge is not just facts or opinions but essentially a rational judgement (48).

Critical realism posits that the world is a stratified open system where structures and mechanisms with causative powers (real domain) generate events, which occur regardless of whether we experience them or not. It suggests that what happens in the world (actual events/Actual domain) is not the same as what is observed/experienced (empirical phenomena/empirical domain). Therefore, critical realism recognizes that we cannot reduce actual events to empirical phenomena or causative mechanisms to empirical phenomena. Critical realism is a meta-theory. As a meta-theory, critical realism is more an ontology than methodology. It focuses primarily on the nature of reality and does not firmly prescribe how to capture or know that reality (51).

Critical realism is not limited to a specific research method or approach, but instead provides a framework for understanding the underlying reality of the phenomena being studied. This allows researchers to use a variety of methods to study different aspects of the same phenomenon and to integrate findings from different methods to gain a more comprehensive understanding. The focus is on understanding the mechanisms and structures that generate the observed phenomena, rather than on a particular methodology or technique. This flexibility in approach

makes critical realism a valuable tool for researchers in a variety of fields who seek to gain a deeper understanding of complex phenomena (49).

In this study, researchers will utilize critical realism's depth reality (52) to overcome the limitations of the empirical nature of the approaches used to examine the stages of concern in earlier studies (53,54). In this study empirical evidence will be obtained on the stages of concerns, in individual, environmental and innovation-related areas, and critical realism's depth reality will be used to understand complexities that are not obvious and visible (**Figure 1**).

Figure 1. The stratified ontology of critical realism

Critical realists use double hermeneutics, abduction and retroduction to conceptualize the actual events and generative mechanisms. Studying objects of nature involves simple hermeneutics while studying social objects involves 'double hermeneutics' as the social scientists are 'interpreting other people's interpretations' (49). It is essential to distinguish what needs to be explained and explanations given by others. Charles S. Peirce was the first to report abductive inference in 1932 (55). It is the logical method of discovering or drawing conclusions from, circumstances and structures that are not given in individual empirical data. Retroduction is the mode of inference in which events are explained by postulating (and identifying) mechanisms which are capable of producing them (52,56). This allows the researcher to go beyond empirical findings and critically examine the in-depth reality of a phenomenon.

Aim of the study

To conduct a critical realist analysis of factors influencing willingness to learn and teach adverse events related PS concepts by nursing educators.

Significance of the study

There is very limited understanding on the current level of patient safety education in Sri Lanka. The results of this study will generate new knowledge on how PS has been perceived by Sri Lankan nursing educators. It will also provide an understanding of the possibilities of integrating internationally recognized PS competencies into local nursing curricula in Sri Lanka. Critically analysed willingness of nursing educators will contribute to equip education institutions with information on individual, environmental, and PS content-related changes and improvements. In addition, this study will enable curriculum developers to proactively address the concerns of nursing educators, leading to better outcomes. Improved education on PS can minimize the effects of adverse events in healthcare and improve the public image of healthcare institutions. Even though the study will not generate a generalizable theory, the critical realist methodology used in this study will be useful for the future researchers, when conducting similar studies in any part of the world.

The conceptual framework

As a summation of Phase 1, a conceptual framework for this study (**Figure 2**) was developed to graphically present the theoretical and methodological underpinnings of the study. Anticipated

events, structures and mechanisms were included acknowledging the inevitable alterations, post-data analysis.

Figure 2. The conceptual framework

Objectives

General objectives

1. To identify and examine the factors that influence nursing educators' willingness to learn and teach adverse events related patient safety concepts.
2. To analyse the underlying structures and generative mechanisms that contribute to the development of variations in willingness to learn and teach patient safety, among nursing educators.

Specific objectives

1. To assess the extent of inclusion of adverse events related patient safety concepts in the curricula of selected nursing programmes.
2. To translate and validate the Sinhala version of the Hurt-Joseph-Cook individual innovativeness questionnaire for use with nursing educators.
3. To evaluate the level of individual innovativeness among nursing educators.
4. To determine the specific stages of concern exhibited by nursing educators regarding the integration of adverse events related patient safety concepts.

5. To explore the correlations between Individual Innovativeness and the stages of concern among nursing educators.
6. To examine the perceived attributes and environmental factors that facilitate or hinder the adoption of adverse events related patient safety concepts by nursing educators.
7. To synthesis the actual events, generative mechanisms and structures that contribute to the facilitation or hindrance of the adoption of adverse events related patient safety concepts.

Materials and methods

In Critical realistic methods, quantitative and qualitative data collection is frequently termed as extensive methods and intensive methods, respectively (49,56). In this study, empirical data collected through extensive methods will be used to explain the phenomenon, while abductive inferencing will be used to identify actual events which are not explicitly linked to the phenomenon. Retroduction will be used to identify structures and generative mechanisms in the real domain.

Study design

Mixed Method Sequential Explanatory (MMSE) (57) design and the staged model for explanatory social sciences (DAARCC) proposed by Danermark (2019) (49), were adopted when designing the research study. DAARCC stands for D- description, A -analytical resolution, A- abduction/theoretical redescription, R- retroduction, C-comparison between different theories

and abstractions, C-concretization and contextualization. Baisley (58), has proposed a Critical Realist Mixed-Methods Sequential Explanatory Design (CRMMSED), a research design combining critical realistic concepts, the staged model for explanatory social sciences and the original mixed-method sequential explanatory design. This research design follows the same design as CRMMSED: extensive data collection and analysis, linking phase, intensive data collection and analysis phase and interpretation phase. Whereas description (D), analytical resolution (A), and abduction/theoretical redescription (A) stages of the DAARCC model recur during conceptualization and data collection (**Table 2**).

Table 2. Comparison of study designs

Sequence in this study		Corresponding CRMMSED phases	Corresponding MMSE phases	Corresponding DAARCC stages
Conceptualization				D- Description,<br>A -Analytical resolution,<br>A- Abduction/theoretical redescription
Data collection and analysis	**Phase 1-** extensive data collection and analysis	1. Extensive data collection and analysis	• Quantitative data collection<br>• Quantitative data analysis	
Data collection and analysis	**Phase 2-** Linking phase	2. Linking phase	• Connecting quantitative and qualitative phases	D- Description,<br>A -Analytical resolution,<br>A- Abduction/theoretical redescription
Data collection and analysis	**Phase 3-** Intensive data collection and analysis	3. Intensive data collection and analysis	• Qualitative data collection<br>• Qualitative data analysis	
**Phase 4-** Interpretation		4. Interpretation phase	• Triangulation of the qualitative and quantitative results	R- Retroduction,<br>C-Comparison between different theories and abstractions,<br>C-Concretization and contextualization

Study setting

The study will be carried out in Nursing Diploma and B.Sc. Nursing programmes that are conducted in state universities (n=8), private universities approved by the University Grants Commission, Sri Lanka (n=2) and Nurses Training Schools (NTS) attached to general and teaching hospitals (n=16) under the Ministry of Health, Sri Lanka.

Study population

Permanent nursing educators (N_{total}=413) who are capable of reading, speaking and comprehending the Sinhala language (N_S≈378).

Inclusion and exclusion criteria

Inclusion criteria:

Permanent academic staff members whose basic qualification is Nursing will be included in the study. Participant should teach at least one core nursing subject (ex. Fundamentals of Nursing, Clinical Nursing subjects, Management in Nursing, etc.) and capable of reading, speaking and comprehending Sinhala language.

Exclusion criteria:

Temporary academic staff will be excluded from the study. Academics who are teaching non-nursing subjects (ex. Anatomy, Physiology, Biochemistry, English, etc.) and those who does not have nursing as their basic qualification will also be excluded.

Sampling technique

The sampling technique for this study involves purposive sampling and stratified random sampling. In **phase 1**, all available curricula in selected institutions will be included in content verification using a purposive sampling technique.

For the self-administered questionnaire, two sets (sample 1 and sample 2) of nursing academics will be recruited. The first set (sample 1) will be used to perform exploratory factor analysis (EFA) of II Scale and will be selected using stratified random sampling. Data from sample 2 will be used to questionnaire survey analysis.

The sample size will be determined based on the rule of thumb of 5-10 subjects per item in instrument validation studies (59) resulting in a sample size of 105 (20 items in II scale, 5:1 subject to variable ratio and adding 5% non-response rate). The participants will be selected from three types of institutions; state universities (SU), private universities (PU), and training schools (NTS) using stratified random sampling.

The second set (sample 2) will be used for the confirmatory factor analysis (CFA). This sample will consist of 185 participants who were selected using stratified random sampling and not included in sample 1.

Sample calculation:

Total population of nursing academics are 413 (SU=99, PU=17, NTS=297). To leverage exclusions due to Sinhala language proficiency 35 (SU=10, PU= 5, NTS=20) were exempted. Approximately 378 were left as the population of nursing educators fluent in Sinhala.

Sample 1 (n_1) for EFA of II Scale (20 items; 1 item: 5 educators)

n_1= 100+non respondents 5% = 105

Subsampling of n_1 using stratifies random method:

n_1 (SU) = 89/378 X 105 =25

n_1 (PU) = 12/378 X 105 =3

n_1 (NTS) = 277/378 X 105 =77

Remaining population for sample 2 (n_2) calculation = 378-105 = 273

Sample 2 for CFA and questionnaire survey

Calculation 1: Using Yamane's formula (60,61) n = N / (1 + N(e)*2), for a population of 273 with a margin of error of 5%, the sample is calculated to be 162. A sample size of 170 results after adding 5% non-respondents.

Calculation 2: Considering factor analysis for the SoC questionnaire, as subsequent validation is recommended by Tsang et al (62), 175 (35 items; 1 item: 5 participants) are required to calculate CFA. A sample size will be 184 results after adding 5% as non-respondents for CFA of SoC questionnaire.

Since calculation 2 results a higher number 184 (n_2) was selected as the sample size.

Subsampling of n_2 using stratifies random method:

n_2 (SU) = (89-25)/273 X 184 =43

n_2 (PU) = (12-3)/273 X 184 =6

n_2 (NTS) = (277-77)/273 X 184 =135

In **phase 2**, semi-structured interviews will be conducted with nursing educators until data saturation is reached. Participants will be selected purposive sampling method.

Study instruments

Self-administered questionnaire

There will be an online questionnaire consisting of three (3) parts; personal characteristics, Stages of Concern Questionnaire (SoC) questionnaire and Individual Innovativeness (II) scale.

Personal characteristics

Details on gender, age, religion, ethnicity, personal income, marital status, education and employment (university, academic/ admin grade, years of teaching experience, years of clinical experience) will be ascertained in this part.

Stages of Concern Questionnaire (SoCQ)

The original SoCQ had been tested for reliability (test/retest reliability ranges from 0.65– 0.68) and validity (alpha-coefficients range from 0.64–0.83) (28,63). SoCQ's Sinhala and Tamil translations (35 items) have been validated in Sri Lanka, and Cronbach alpha of the Sinhala version (mean) was reported to be 0.78 indicating good internal consistency. Content validity has been evaluated by ten (10) language and content experts (64). Psychometric properties of a Turkish translation has been shown to have weak internal consistency and model fit (65). The reliability for four of the seven stages was low (<0.70), and the inter-correlational matrix did not provide support for the hypothesized relationships among the stages. A shortened (16-item)

version has been suggested to increase reliability (0.66- 0.82) and validity. A validation study in Hong Kong (66), has found that all the fit indices were not satisfactory and the correlations among some factors were too high. They have suggested a 5-stage model with 22 items. The item-total correlations of the 22 items ranged from 0.46 to 0.70 and the alphas of the five scales varied from 0.75 to 0.84. During this study construct validity of all 3 models will be evaluated via Confirmatory Factor Analysis (CFA) to identify the best model fit in Sri Lankan context.

The validated SoC questionnaire will be used along with the change of “name of the innovation” from “5E instructional method” to “adverse events related patient safety concepts (AER-PS)”.

Individual Innovativeness (II) scale

This scale has been developed to measure an individual’s orientation towards innovation (33). This has shown strong psychometric characteristics; Cronbach α above 0.80 and it has been used as a valid measure of general innovativeness in earlier studies (67–69). A study done in Pakistan found reliability to be above 0.75 and values of model fit were close to 0.90 (70).

The scale has 20 items and it ranges from options Strongly Disagree = 1; Disagree = 2; Neutral = 3; Agree = 4; Strongly Agree = 5 respectively to rate each item.

Scores above 80 are classified as Innovators.

Scores between 69 and 80 are classified as Early Adopters.

Scores between 57 and 68 are classified as Early Majority.

Scores between 46 and 56 are classified as Late Majority.

Scores below 46 are classified as Laggards/Traditionalists.

Translation of II scale to Sinhala language, cross-cultural adaptation and psychometric analysis will be done following the established guidelines on cross-cultural adaptation (71) and psychometric analysis (72,73).

Data collection

Data collection is not anonymised. In order to protect privacy and confidentiality; names and university affiliations will be replaced with codes during data processing. Any data requested during publications will also be anonymised. Data will be stored in password-protected documents on a secure Google Drive linked to the researcher's faculty email address and later data will be transferred to an external storage device. After 10 years from the submission of the thesis, the data will be deleted. Only the investigator and supervisor will have access to non-anonymised data.

Phase 1: Extensive/ quantitative procedures

I. Curriculum content verification.

This will be used as a way of fact-checking/validating the adverse events related (AER) content of PS is being taught. Soft copies of the curricula will be requested from deans of the faculties and the Director-Nursing Education, Ministry of Health, Sri Lanka. Upon receiving, a word search of

the relevant PS content will be carried out. A Microsoft Excel matrix will be used to document which AER-PS concepts are mentioned in curricula.

II. Self-administered questionnaire

Authorization will be obtained from the relevant deans of faculties and training schools' principles to collect data from their academic staff members and to verify the curricula content. A random sample of nursing educators from each institution category (state universities, private universities and training schools) will be selected. An online questionnaire link will be emailed or texted to selected nursing educators. The selection of participants will not be based on ethnicity. An information sheet will be provided to participants and will contain a brief description of the study, ethical approval, details of investigators, the procedure for asking questions, and participants' rights. Prior to accessing the questionnaire, a consent form will be provided for participants to acknowledge their voluntary participation and the option to withdraw their consent at any time. Non-respondents will receive a reminder on the following week and a phone reminder on the 2nd week.

Phase 3: Intensive/ qualitative procedures

Semi-structured interviews will be conducted to identify perceived attributes of selected PS concepts and environmental attributes which facilitate or inhibit the adoption of selected concepts. One-to-one interviewing of the selected participants will be carried out in person or virtually according to the participant's preference. If done in person, it will be at the participants' academic institution. An interview guide developed by the principal investigator will be used (**S1**

File). Participants will be requested to go through the information sheet and provide consent form prior the interview. Interview will be conducted in participants preferred language (Sinhala, Tamil or English). A Tamil-Sinhala-English translator will be available during the interview for assistance upon permission from the participant. For audio recordings, participants will be advised not to identify themselves or third parties. Digital recordings of the interviews and transcripts will be password protected and saved on a secure Google Drive linked to the researcher's faculty email address. The data will later be transferred to an external storage device once the analysis is completed. Transcribing will be done by the researcher. All transcripts will identify interviewees by codes. Participants will be allowed to review the transcription and provide written consent to use the recording.

Secondary data (policies, news reports, press releases) will be analysed to identify any significant events. Online resources will be retrieved using the keywords "Sri Lanka", "Patient safety", "policies", "news reports", and "press releases" in internet search engines.

Data analysis

Phase 1: Extensive data

Curriculum content verification

percentages will be calculated on the inclusion of five selected concepts in each curriculum. The selected adverse events related to patient safety concepts are, 1) the definition of adverse events

(AE) in healthcare, 2) Adverse event reporting, 3) human factors in healthcare safety, 4) system factors in healthcare safety and 5) root cause analysis (RCA).

Students' knowledge outcomes mentioned in WHO-MPSC relevant to the above concepts (total 11) and introduction to incident reporting form provided by the Directorate of Healthcare Quality and Safety, Sri Lanka will be checked against the local curricula of each participating institution (**S2 File**). Observations will be marked under 3 categories "Not mentioned”, "Partially mentioned" and "Explicitly mentioned". Percentages for each category will be calculated in all participating institutes.

*Self- administered questionnaire***-** *personal characteristics, SoCQ and II scale.*

Psychometric properties of II scales’ Sinhala version will be assessed. Five (5) experts will be consulted to comment on the content validity of the scale. Content Validity Index (CVI) will be calculated to assess the relevancy and clarity of items. Statistical analysis will be conducted using IBM SPSS Statistics version 20.0 for Windows. Reliability will be assessed using measurements of internal consistency (Cronbach’s alpha coefficient and Composite reliability). Exploratory factor analysis (EFA) will be conducted to identify the constructs of the instrument using Principal Component Analysis (PCA), Varimax rotation and Kaiser Normalization. Construct validity will be evaluated using convergent and divergent validity and confirmatory factor analysis (CFA). Thereafter, Individual innovativeness categories of nursing educators will be identified using the II scoring matrix (**S3. File**).

The validity and reliability of the adapted SoCQ Sinhala version will be assessed. SoC quick scoring device (**S4. Fig.**) will be used to calculate the scores of individual nursing educators and determine stages of concern in each. Frequency, mean, SD and Percentiles will also be calculated.

Phase 3: Linking phase

Correlations of curricula content, personal characteristics, II scale and SoCQ will be assessed using SPSS and AMOS. The results will be used to determine how Individual innovativeness and personal characteristics affect SoC. An attempt will be made to determine if the SoC of nursing educators affect the integration of AER- PS content in nursing curricula. Actual events will be identified and re-described using data collected in Phase 1 and the conceptual framework.

Phase 4: Intensive data

Thematic analysis will be used to analyse transcriptions of semi-structured interviews. Themes will be identified using NVivo. Deductive thematic analysis will be conducted, based on the conceptual framework presented. Yet, the researchers will not get limited by the framework and will also include inductive analysis, given that new constructs could appear during the analysis phase.

Content analysis will be used to analyse secondary data (policies, news reports, and press releases). Characteristics of the document's content will be described by examining who says what, to whom, and with what effect (74).

Interpretation of data

To comprehend the factors that impact nursing educators' willingness to learn and teach new PS concepts, both quantitative and qualitative analyses will be triangulated (75). An attempt will be made at identifying how quantitative correlations (personal characteristics, SoC score, II score, curriculum content) and qualitative themes generated (on perceived attributes of patient safety education, resources, opportunities, information and support) would integrate to provide an informative, layered explanation of why nursing educators' perceptions have become what they are. Retroductive approach and causal analysis (56) will be used to identify individual, environmental and curricula-related causes which could have contributed to the willingness to learn and teach.

Ethical Considerations and Declarations

Ethical clearance to conduct this study has been obtained by the Ethical Review Committee at the Faculty of Graduate Studies, University of Colombo (FGS/ERC/2022/013).

The researchers have obtained necessary permissions from the copyright owners to conduct a translation and validation of the Individual Innovativeness scale, as well as to reuse the Stages of Concern questionnaire and its corresponding Sinhala translation.

Risk-Benefit Assessment

There are no interventions that are used during the study which requires special precautions. Therefore, there are no potential risks to the participants. There are no immediate benefits to the participants either. Social and scientific value of the study are mentioned in significance of

the study. Participants will be directed to WHO-Multi Professional Patient Safety Curriculum and other patient safety reading materials at the end of the questionnaire.

Participants rights and consent

An email and/or message will be sent to all nursing educators who have met inclusion criteria requesting to participate in the study. Participants will be recruited through volunteer participation. A brief description of the study, ethical approval, details of investigator and supervisor will be provided in the information sheet hyperlinked to the questionnaire. In 2nd section in google form, there will be five questions including, "have you read the information sheet? Do you agree to take part in this study?" If they select YES for all 5 questions and proceed to the questionnaire, it will be considered as consent given. If they select NO for at least one question, they will exit from the google form without proceeding to the questionnaire. In the information sheet, it will be stated that the participants can withdraw their consent to participate in the study at any point, even after submitting the questionnaire or after participating in interviews. Their data will be duly removed from the study, if they opt out. Interview participants had the right to ask questions, to withdraw from the study at any time, to withdraw their data, to decline to answer any question, to turn off the recorder. The information sheet will provide contact details of the investigator and the ethical approval committee to register any complaints on the study. Interview participants will have the right to check transcription summaries for accuracy and to receive a summary of the findings of the completed research.

Confidentiality and Privacy

Demographic data such as age, designation, university will be requested to be used in finding out associations and correlation. Names of the participants will be requested in order to contact participants for semi-structured interviews. Name of the participant and university will be replaced with codes when processing data to ensure privacy of the participant. If data are requested during publications, anonymity of the participants will be ensured. Security of data will be ensured. Spreadsheets and recordings will be saved in the google drive attached to the researcher's faculty e-mail address provided by University of Sri Jayewardenepura with password protected access only permitted to the researcher. Data will be transported from google drive to an external data storage device post thesis submission in order to free cloud storage space. Data will be saved in password protected documents and will be deleted 10 years after submission of thesis. Only the principal investigator and supervisor will have the access to the data.

Fair participant selection and vulnerability

Nursing academics who fulfil the inclusion criteria are selected regardless of ethnicity for the online questionnaire. However, due to the lack of Tamil language proficiency of the researcher, if there will be any interviews with Tamil speaking participants, a Tamil-Sinhala-English translator will be available during the interview for assistance upon permission from the participant. If the Tamil speaking participant is fluent in Sinhala and or English, interview will be conducted in participants preferred language (Sinhala/English).

Discussion

This study aims to identify the concerns of nursing educators in adopting adverse events related to PS concepts, as well as the factors contributing to these concerns. The study will also explore the impact of individual innovativeness on these concerns. The findings of this study will provide valuable insight into the factors that influence nursing educators' concerns, which can be used to propose changes and improvements to individual, environmental, and PS content. Ultimately, improved education on PS will benefit patients by improving their experiences and outcomes at healthcare facilities.

Previous studies have identified the impact of nursing educators’ concerns or perspectives in PS education (76–79), but few have delved into the specific reasons behind these concerns. It is important to address any underlying causes of these concerns to ensure full participation and commitment from nursing educators. However, in a complex society where causation is often multifaceted and debatable, it can be challenging to identify the exact causes of a phenomenon. Critical realism acknowledges this complexity and proposes the emergence of actual events from generative mechanisms and structures.

While the primary focus is on Sri Lanka, the study's insights and methodological approach have broader applicability to similar studies worldwide. By understanding the underlying mechanisms shaping nursing education in Sri Lanka, the research aims to provide context-specific suggestions, while also contributing to the broader knowledge base in education research.

The researchers recognize that critical realism is a topic of ongoing debate and that some may have reservations about its practical applications, particularly in the three domains of reality (real, actual and empirical) (80). Additionally, there is limited research on healthcare education

utilizing the staged model for explanatory social sciences or proposed Critical Realist Mixed-Methods Sequential Explanatory Design. Conducting a study that combines healthcare education, critical realism, innovativeness, and stages of concern is a challenging and interdisciplinary undertaking. The researchers are aware of these challenges and will strive to navigate them using the best available scientific reasoning.

The study results will only be generalizable among Sri Lankan nursing educators who are fluent in Sinhala, due to the use of Sinhala translations of study instruments. When a study is conducted in the English language in a country where the population is composed of non-native English speakers, it could significantly alter the outcome (62,71,81,82). There is a possibility that the level of English proficiency of the participants affects the quality, nuances, and richness of the responses they provide. In a self-administered questionnaire, it could influence the way they perceive questions and their intentions. Therefore, validating the Individual Innovativeness scale in local languages was a necessity before using it. However, due to the lack of Tamil-fluent educators, validating the Tamil translation was not feasible. Hence, only the Sinhala translation will be validated and used in the study.

There is a possibility that those who are not willing to accept a new idea or new PS concepts in particular, will refuse to participate in this study. If so, it won’t be possible to identify/synthesis what causes them to deny this new concept.

The study's findings will be disseminated through several channels. Firstly, they will be presented at research conferences and submitted for publication in peer-reviewed scientific journals. Additionally, a summary of the results will be provided to nursing department heads and participating educators. A report of the study's findings will also be shared with the Director of

Nursing Education and the Directorate of Healthcare Quality and PS in Sri Lanka. This will ensure that the results of this study are made available to relevant stakeholders and contribute to the wider conversation around PS education in nursing.

Authors' contributions

DMAP contributed to the conceptualization of the study, developing methodology, and manuscript writing and SSP contributed to developing methodology, manuscript reviewing and finalising.

The authors collectively provide permission to publish this manuscript in PLOS One journal.

Acknowledgements

Not applicable

References

1. WHO. Global strategic directions for NURSING AND MIDWIFERY [Internet]. 2021 [cited 2023 Jan 10]. Available from: https://apps.who.int/iris/bitstream/handle/10665/344562/9789240033863-eng.pdf

2. WHO. Patient safety [Internet]. 2022 [cited 2022 Oct 3]. Available from:

https://www.who.int/health-topics/patient-safety#tab=tab_1

3. Walton M, Jeffery H, Van Staalduinen S, Klein L, Rothnie I. When should students learn about ethics, professionalism and patient safety? Clin Teach. 2013 Aug;10(4):224–9.

4. Jang H, Lee NJ. Patient safety competency and educational needs of nursing educators in South Korea. PLoS One. 2017 Sep 1;12(9).

5. QSEN [Internet]. [cited 2022 Sep 29]. Available from: https://qsen.org/competencies/

6. The Australian Council for Safety and Quality in Health Care. National Patient Safety Education Framework [Internet]. 2005 [cited 2022 Sep 28]. Available from: http://www.safetyandquality.gov.au/

7. Lee NJ, An JY, Song TM, Jang H, Park SY. Psychometric Evaluation of a Patient Safety Competency Self-Evaluation Tool for Nursing Students. J Nurs Educ. 2014;53(10):550–62.

8. Canadian Patient Safety Institute. The Safety Competencies: Enhancing Patient Safety Across the Health Professions. [Internet]. 2nd ed. Edmonton, Alberta; 2020 [cited 2022 Sep 28]. Available from: www.patientsafetyinstitute.ca

9. Pelzang R, Hutchinson AM. How Is Patient Safety Understood by Healthcare Professionals? The Case of Bhutan. J Patient Saf [Internet]. 2017;16(1):106–9. Available from: https://www.ncbi.nlm.nih.gov/pmc/articles/PMC7046141/

10. Usher K, Woods C, Conway J, Lea J, Parker V, Barrett F, et al. Patient safety content and delivery in pre-registration nursing curricula: A national cross-sectional survey study. Nurse Educ Today [Internet]. 2018;66(March):82–9. Available from: https://doi.org/10.1016/j.nedt.2018.04.013

11. Vaismoradi M, Salsali M, Marck P. Patient safety: Nursing students' perspectives and the

role of nursing education to provide safe care. Int Nurs Rev. 2011;58(4):434–42.
12. Ginsburg LR, Dhingra-Kumar N, Donaldson LJ. What stage are low-income and middle-income countries (LMICs) at with patient safety curriculum implementation and what are the barriers to implementation? A two-stage cross-sectional study. BMJ Open. 2017;7(6):1–10.
13. Walton MM, Elliott SL. Improving safety and quality: How can education help? Med J Aust [Internet]. 2006;184(10 SUPPL.):S60–4. Available from: http://www.embase.com/search/results?subaction=viewrecord&from=export&id=L43732927
14. Mansour M. Examining patient safety education in pre-registration nursing curriculum: Qualitative study. J Nurs Educ Pract [Internet]. 2013;3:12. Available from: https://doi.org/10.5430/jnep.v3n12p157
15. Sherwood G, Drenkard K. Quality and safety curricula in nursing education: Matching practice realities. Nurs Outlook [Internet]. 2007;55(3):151–5. Available from: https://www.nursingoutlook.org/article/S0029-6554(07)00061-9/fulltext
16. Wong BM, Etchells EE, Kuper A, Levinson W, Shojania KG. Teaching quality improvement and patient safety to trainees: A systematic review. Acad Med [Internet]. 2010;85(9):1425–39. Available from: https://doi.org/10.1097/ACM.0b013e3181e2d0c6
17. Armitage G, Cracknell A, Forrest K, Sandars J. Twelve tips for implementing a patient safety curriculum in an undergraduate programme in medicine. Med Teach [Internet]. 2011;33(7):535–40. Available from: https://doi.org/10.3109/0142159X.2010.546449
18. Walton M, Woodward H, van Staalduinen S, Lemer C, Greaves F, Noble D, et al.

Republished paper: The WHO patient safety curriculum guide for medical schools. Postgrad Med J [Internet]. 2011;87(1026):317–21. Available from: https://doi.org/10.1136/pgmj.2009.036970rep

19. Landefeld J, Sivaraman R, Arora N. Barriers to Improving Patient Safety in India: Focus Groups with Providers in the Southern State of Kerala. Indian J Community Med [Internet]. 2015;40(2):116. Available from: https://www.ncbi.nlm.nih.gov/pmc/articles/PMC4389498/

20. Madhok R, Vaid S, Carson-Stevens A, Panesar S, Mathew J, Roy N, et al. Promoting patient safety in India: Situational analysis and the way forward. Natl Med J India [Internet]. 2014;27(4):217–23. Available from: http://www.nmji.in/archives/Volume-27/Issue-4/Medicine-and-Socity.pdf%0Ahttp://ovidsp.ovid.com/ovidweb.cgi?T=JS&PAGE=reference&D=emed15&NEWS=N&AN=602220479

21. Mallawarachchi SMNSM, Dharmarathna GS. A systematic assessment of adverse event reporting in selected state hospitals in Sri Lanka. J Surg Med [Internet]. 2022 Apr 1 [cited 2022 Sep 28];6(4):494–7. Available from: https://ojs.selsistem.com.tr/doi/10.28982/josam.897752

22. Mendis S. Medical error and negligence. Isl [Internet]. 2016 Apr;12. Available from: http://island.lk/index.php?page_cat=article-details&page=article-details&code_title=143690%3E

23. Wijemanne WMUS, Sridharan S, Gamini LPS. Strengthening of Knowledge and Skills on Patient Safety of Nursing Officers' in a Teaching Hospital in Sri-Lanka. 2019 Dec 1 [cited

2022 Sep 28];1(2):75. Available from: https://sljms.sljol.info/articles/abstract/10.4038/sljms.v1i2.50/

24. Piyarathna JG, Seneviratne SMKS. Knowledge and attitudes towards patient safety among nursing students at three state universities in Sri Lanka [Abstract]. In: Peradeniya University International Research Sessions, Sri Lanka [Internet]. 2016. p. 2016. Available from: http://dr.lib.sjp.ac.lk/handle/123456789/6265

25. WHO SEARO. Regional strategy for patient safety in the WHO South-East Asia Region. 2014.

26. WHO. WHO Patient safety curriculum guide: multi-professional edition [Internet]. World Health Organization, editor. malta: WHO; 2011. 272 p. Available from: https://www.who.int/publications/i/item/9789241501958

27. Farley D, Zheng H, Rousi E, Leotsakos A. Evaluation of the WHO Multi - Professional Patient Safety Curriculum Guide. 2013; Available from: http://www.who.int/patientsafety/education/curriculum/EN_PSP_Curriculum_Evaluation/en/

28. George AA, Hall GE, Stiegelbauer SM, Southwest Educational Development Laboratory. Measuring implementation in schools : the stages of concern questionnaire [Internet]. Austin, TX: SEDL. Available from; 2006. 98 p. Available from: http://www.sedl.org/pubs/catalog/items/cbam17.html

29. Buabeng-Andoh C. Factors influencing teachers ’ adoption and integration of information and communication technology into teaching: A review of the literature. Int J Educ Dev Using Inf Commun Technol [Internet]. 2012;8(1):136–55. Available from:

https://files.eric.ed.gov/fulltext/EJ1084227.pdf

30. Lai HM, Chen CP. Factors influencing secondary school teachers' adoption of teaching blogs. Comput Educ [Internet]. 2011;56(4):948–60. Available from: https://www.sciencedirect.com/science/article/abs/pii/S0360131510003313

31. Rogers EM. Diffusion of Innovations, 5th Edition [Internet]. Mexico: Simon and Schuster; 2003 [cited 2022 Sep 30]. 576 p. Available from: https://books.google.com/books/about/Diffusion_of_Innovations_5th_Edition.html?id=9U1K5LjUOwEC

32. Aldahdouh TZ, Nokelainen P, Korhonen V. Innovativeness of staff in higher education do implicit theories and goal orientations matter? Int J High Educ [Internet]. 2018 [cited 2022 Sep 30];7(2):43–57. Available from: https://files.eric.ed.gov/fulltext/EJ1172498.pdf

33. Hurt HT, Joseph K, Cook CD. Scales for the Measurement of Innovativeness. Hum Commun Res [Internet]. 1977;4(1):58–65. Available from: https://academic.oup.com/hcr/article-abstract/4/1/58/4626783?redirectedFrom=fulltext

34. Coklar AN. Individual Innovativeness Levels of Educational Administrators Individual Innovativeness Levels of Educational Administrators Individual Innovativeness Levels of Educational Administrators. Digit Educ Rev [Internet]. 2012 [cited 2023 Mar 20]; Available from: http://greav.ub.edu/der/

35. Akgün F. Investigation of Instructional Technology Acceptance and Individual Innovativeness of Academicians. Turkish Online J Qual Inq [Internet]. 2017 Jul 30 [cited 2023 Mar 20];8(3):291–322. Available from:

https://dergipark.org.tr/en/pub/tojqi/issue/30703/292135

36. Arts JWC, Frambach RT, Bijmolt THA. Generalizations on consumer innovation adoption: A meta-analysis on drivers of intention and behavior. Int J Res Mark [Internet]. 2011;28(2):134–44. Available from: http://dx.doi.org/10.1016/j.ijresmar.2010.11.002

37. Bartels J, Reinders MJ. Consumer innovativeness and its correlates: A propositional inventory for future research. J Bus Res [Internet]. 2011 [cited 2022 Sep 30];64:601–9. Available from: https://www.sciencedirect.com/science/article/abs/pii/S0148296310001049

38. Talukder M. Factors affecting the adoption of technological innovation by individual employees: An Australian study. Procedia-Social Behav Sci [Internet]. 2012 [cited 2023 Mar 20];40:52–7. Available from: https://www.sciencedirect.com/science/article/pii/S1877042812006271

39. Laschinger HKS. A theoretical approach to studying work empowerment in nursing: a review of studies testing Kanter's theory of structural power in organizations. Nurs Adm Q. 1996 Dec;20(2):25–41.

40. Manojlovich M. Promoting nurses' self-efficacy: A leadership strategy to improve practice. J Nurs Adm [Internet]. 2005;35(5):271–8. Available from: https://journals.lww.com/jonajournal/Abstract/2005/05000/Promoting_Nurses__Self_efficacy__A_Leadership.11.aspx

41. Knol J, Van Linge R, Tergooiziekenhuizen JK, Van R. Innovative behaviour: The effect of structural and psychological empowerment on nurses. J Adv Nurs [Internet]. 2009 [cited 2022 Sep 30];65(2):359–70. Available from:

https://onlinelibrary.wiley.com/doi/10.1111/j.1365-2648.2008.04876.x

42. Kanter RM. Men and women of the corporation: New edition. [Internet]. basic books; 2008 [cited 2022 Sep 30]. 409 p. Available from: https://books.google.com/books/about/Men_and_Women_of_the_Corporation.html?id=B-5GztW4IZQC

43. Frambach R, Schillewaert N. Organizational innovation adoption: A multi-level framework of determinants and opportunities for future research. J Bus Res [Internet]. 2002 [cited 2023 Jul 12]; Available from: https://www.sciencedirect.com/science/article/pii/S0148296300001521

44. Uyangoda J. Writing research proposals in the sosial sciences and humanities : a theoritical and practical guide [Internet]. Social Scientists' Association; 2010 [cited 2022 Sep 30]. 135 p. Available from: https://books.google.com/books/about/Writing_Research_Proposals_in_the_Sosial.html?id=ko7cjwEACAAJ

45. Walter M. Social research methods [Internet]. 2nd ed. Oxford University Press; 2010 [cited 2023 Jul 27]. 510 p. Available from: https://books.google.com/books/about/Social_Research_Methods.html?id=bdchPwAACAAJ

46. Uyangoda J. Social Research: Philosophical and Methodological Foundations [Internet]. Social Scientists' Association; 2015 [cited 2022 Sep 30]. 657 p. Available from: https://books.google.lk/books/about/Social_Research.html?id=cZfUnQAACAAJ&redir_esc=y

47. Sale JEM, Lohfeld LH, Joseph' S, Brazil K. Revisiting the Quantitative-Qualitative Debate: Implications for Mixed-Methods Research. [cited 2024 Apr 20]; Available from: https://link.springer.com/article/10.1023/A:1014301607592

48. Bhaskar R. Theorising ontology. In: Lawson C, Latsis JS, Martins N, editors. Contributions to Social Ontology: Routledge Studies in Critical Realism [Internet]. Routledge; 2007. p. 192–204. Available from: https://books.google.lk/books?hl=en&lr=&id=ZV8LigP2_xYC&oi=fnd&pg=PA192&ots=LDjgD0jQba&sig=QJKTtV1zeT6UxlEPXomx_xLWQbI&redir_esc=y#v=onepage&q&f=false

49. Danermark B, Ekström M, Karlsson JC. Explaining society: Critical realism in the social sciences [Internet]. 2nd ed. Taylor and Francis; 2019 [cited 2022 Sep 30]. 1–228 p. Available from: https://www.taylorfrancis.com/books/mono/10.4324/9781351017831/explaining-society-berth-danermark-mats-ekström-jan-ch-karlsson

50. Al-Amoudi. Where constructionism and critical realism converge: interrogating the domain of epistemological relativism. Organ Stud [Internet]. 2011 Jan [cited 2022 Sep 30];32(1):27–46. Available from: https://centaur.reading.ac.uk/24956/2/Al-Amoudi_and_Willmott_2011_centaur.pdf

51. Allana S, Clark A. Applying Meta-Theory to Qualitative and Mixed-Methods Research: A Discussion of Critical Realism and Heart Failure Disease Management Interventions Research. Int J Qual Methods [Internet]. 2018 [cited 2022 Sep 30];17:1–9. Available from: https://us.sagepub.com/en-us/nam/open-access-at-sage

52. Bhaskar R. A Realist Theory of Science (1975). 2nd ed. London and New York: Taylor &

Francis e-Library; 2008.

53. Al-Furaih SAA, Al-Awidi HM. Teachers’ Change Readiness for the Adoption of Smartphone Technology: Personal Concerns and Technological Competency. Technol Knowl Learn. 2020 Jun 1;25(2):409–32.

54. Geng J, Jong MSY, Chai CS. Hong Kong Teachers’ Self-efficacy and Concerns About STEM Education. Asia-Pacific Educ Res [Internet]. 2019 Feb 15 [cited 2023 Jul 17];28(1):35–45. Available from: https://link.springer.com/article/10.1007/s40299-018-0414-1

55. Peirce CS. Collected Papers of Charles Sanders Peirce: Volume II Elements of Logic [Internet]. 1932 [cited 2022 Sep 30]. 962 p. Available from: https://www.hup.harvard.edu/catalog.php?isbn=9780674138001

56. Sayer A. Method in Social Science, A realist approach [Internet]. 2nd ed. London and New York: Routledge; 2010. 336 p. Available from: https://www.taylorfrancis.com/books/mono/10.4324/9780203850374/method-social-science-andrew-sayer

57. Ivankova N V., Creswell JW, Stick SL. Using Mixed-Methods Sequential Explanatory Design: From Theory to Practice. Field methods [Internet]. 2006 [cited 2022 Oct 1];18(1):3–20. Available from: https://journals.sagepub.com/doi/abs/10.1177/1525822x05282260

58. Gail Baisley H. Pedagogies of presence ; contemplative education across the disciplines in Aotearoa New Zealand [Internet]. Massey University; 2020 [cited 2022 Oct 1]. Available from: https://mro.massey.ac.nz/handle/10179/15782

59. Tabachnick BG, Fidell LS. Using Multivariate Statistics [Internet]. 7th ed. Pearson

Education; 2019 [cited 2022 Oct 1]. 815 p. Available from: https://www.pearson.com/en-us/subject-catalog/p/using-multivariate-statistics/P200000003097/9780137526543

60. Yamane T. Statistics: an introductory analysis. 3rd ed. New York: Harper and Row; 1973. 1130 p.

61. Israel GD. Determining sample size [Internet]. 1992 [cited 2023 Jul 18]. Available from: https://www.researchgate.net/profile/Subhash-Basu-3/post/how_could_i_determine_sample_size_for_my_study/attachment/5ebaa4924f9a520001e613b6/AS:890361492811785@1589290130539/download/samplesize1.pdf

62. Tsang S, Royse CF, Terkawi AS. Guidelines for developing, translating, and validating a questionnaire in perioperative and pain medicine [Internet]. Vol. 11, Saudi Journal of Anaesthesia. 2017 [cited 2022 Oct 1]. p. S80–9. Available from: https://www.saudija.org/article.asp?issn=1658-354X;year=2017;volume=11;issue=5;spage=80;epage=89;aulast=Tsang

63. Hall GE, George AA. Stages of concern about innovation: The concept, initial verification and some implications [Internet]. 1979 [cited 2022 Sep 29]. 38 p. Available from: https://files.eric.ed.gov/fulltext/ED188716.pdf

64. nor Puteh SN, Nawastheen FM. An evaluation on the implementation of 5E instructional model in teaching geography in Sri Lanka. Middle East J Sci Res [Internet]. 2013 [cited 2022 Oct 1];16(5):721–8. Available from: https://www.idosi.org/mejsr/mejsr16(5)13/20.pdf

65. Goktalay SB, Cangur S. Assessing the reliability and validity of the Turkish version of the stages of concern questionnaire. Egit Arastirmalari - Eurasian J Educ Res. 2008;(33):55–

72.

66. Cheung D, Hattie J, Ng D. Reexamining the stages of concern questionnaire: A test of alternative models. J Educ Res. 2001;94(4):226–36.

67. Goldsmith RE. The validity of a scale to measure global innovativeness. J Appl Bus Res [Internet]. 1991 [cited 2022 Oct 1];7(2):89–97. Available from: https://clutejournals.com/index.php/JABR/article/view/6249

68. Pallister JG, Foxall GR. Psychometric properties of the Hurt-Joseph-Cook scales for the measurement of innovativeness. Technovation. 1998;18(11):663–75.

69. Aldahdouh TZ, Korhonen V, Nokelainen P. What contributes to individual innovativeness? A multilevel perspective. Int J Innov Stud. 2019 Jun 1;3(2):23–39.

70. Ali I. Personality traits, individual innovativeness and satisfaction with life. J Innov Knowl [Internet]. 2019;4(1):38–46. Available from: https://doi.org/10.1016/j.jik.2017.11.002

71. Beaton DE, Bombardier C, Guillemin F, Ferraz MB. Guidelines for the process of cross-cultural adaptation of self-report measures. Spine (Phila Pa 1976) [Internet]. 2000 [cited 2022 Oct 1];25(24):3186–91. Available from: https://journals.lww.com/spinejournal/Fulltext/2000/12150/Guidelines_for_the_Process_of_Cross_Cultural.14.aspx

72. American Educational Research Association, American Psyhological Association, National Council on Measurement in, Education, Fernández-Ballesteros R. Standard for Educational and Psychological Testing [Internet]. 2008 [cited 2023 Jan 9]. Available from: https://www.apa.org/science/programs/testing/standards

73. David, Streiner, Norman GR, Cairney J. Health Measurement Scales_ a Practical Guide to

Their Development and Use-Oxford University Press. Pap Knowl Towar a Media Hist Doc. 2014;

74. Vaismoradi M, Turunen H, Bondas T. Content analysis and thematic analysis: Implications for conducting a qualitative descriptive study [Internet]. Vol. 15, Nursing and Health Sciences. 2013 [cited 2022 Oct 1]. p. 398–405. Available from: https://onlinelibrary.wiley.com/doi/abs/10.1111/nhs.12048

75. Creswell JW, Clark VLP, Gutmann ML, Hanson WE. Advanced Mixed Methods Research Designs. In: Tashakkori A, Teddlie C, editors. Handbook of mixed methods in social & behavioral research. CA: Thousand Oaks,CA: Sage; 2003. p. pp209-240.

76. Ginsburg LR, Dhingra-Kumar N, Donaldson LJ. What stage are low-income and middle-income countries (LMICs) at with patient safety curriculum implementation and what are the barriers to implementation? A two-stage cross-sectional study [Internet]. 2017;7(6):1–10. Available from: https://doi.org/10.1136/bmjopen-2017-016110

77. Mbuthia NN, Moleki MM. Perspectives on patient safety education in the Kenyan preregistration nursing curriculum. Afr J Nurs Midwifery [Internet]. 2018;20(2). Available from: https://journals.co.za/doi/pdf/10.25159/2520-5293/3866https://unisapressjournals.co.za/index.php/AJNM/article/download/3866/2882 NS -

78. Wu AW, Busch IM. Patient safety: A new basic science for professional education. GMS J Med Educ. 2019;36(2).

79. Lee SE, Dathinten VS, Do H. Patient safety education in pre-registration nursing programmes in South Korea. Int Nurs Rev [Internet]. 2020;67(4):512–8. Available from:

https://onlinelibrary.wiley.com/doi/abs/10.1111/inr.12630 NS -
80. Groff R. critical realism network. 2022 [cited 2023 Jan 19]. There Aren't Really Three Domains: or, Metaphor Is Great, Except When It's Not – Critical Realism Network. Available from: https://criticalrealismnetwork.org/2022/07/21/there-arent-really-three-domains/
81. Guillemin F, Bombardier C, Beaton D. Cross-cultural adaptation of health-related quality of life measures: Literature review and proposed guidelines. J Clin Epidemiol [Internet]. 1993 Dec 1 [cited 2023 Jul 17];46(12):1417–32. Available from: http://www.jclinepi.com/article/089543569390142N/fulltext
82. Gjersing L, Caplehorn JR, Clausen T. Cross-cultural adaptation of research instruments: Language, setting, time and statistical considerations. BMC Med Res Methodol. 2010;10.

Supporting documents

S1. File. Interview Protocol

S2. File. Curricula data extraction sheet

S3. File. Individual Innovativeness Scale score calculation

S4. Fig. SoC quick scoring device

 Click here to access/download;Figure;Figure 1.tif

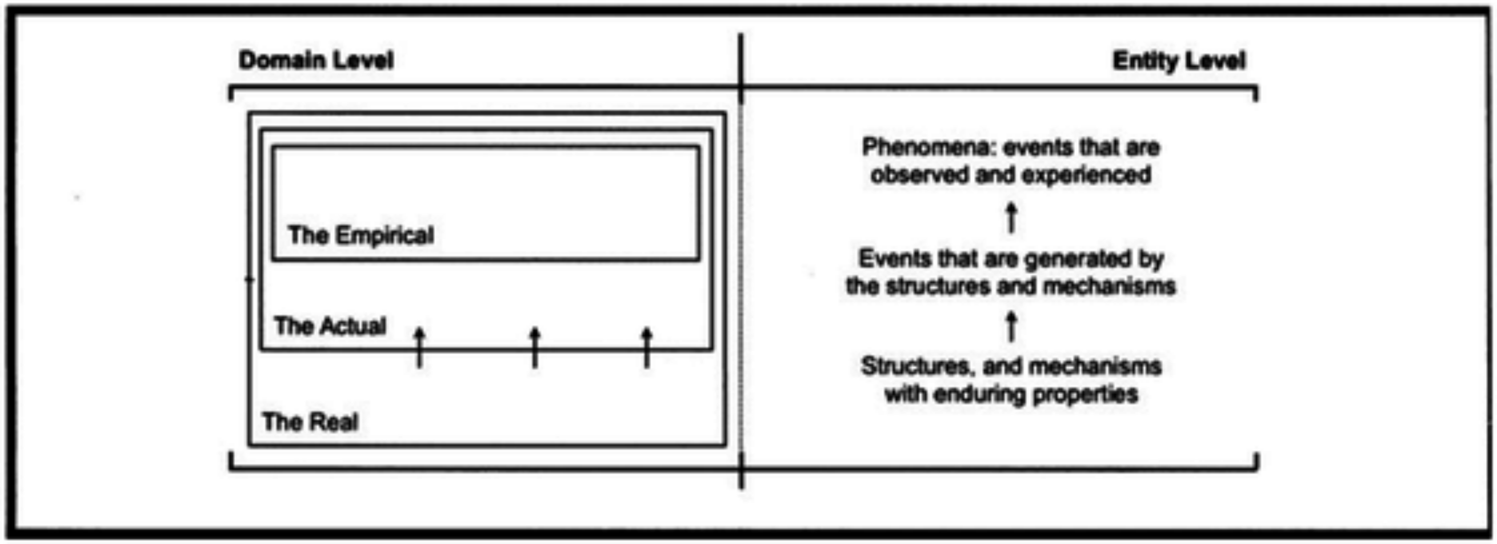

Figure 1: The stratified ontology of critical realism as presented by Zachariadis M, Scott S, Barrett M. Methodological Implications of Critical Realism for Mixed-Methods Research. MIS Q [Internet]. 2013;37(September):855–79. Available from: www.jstor.org/stable/43826004

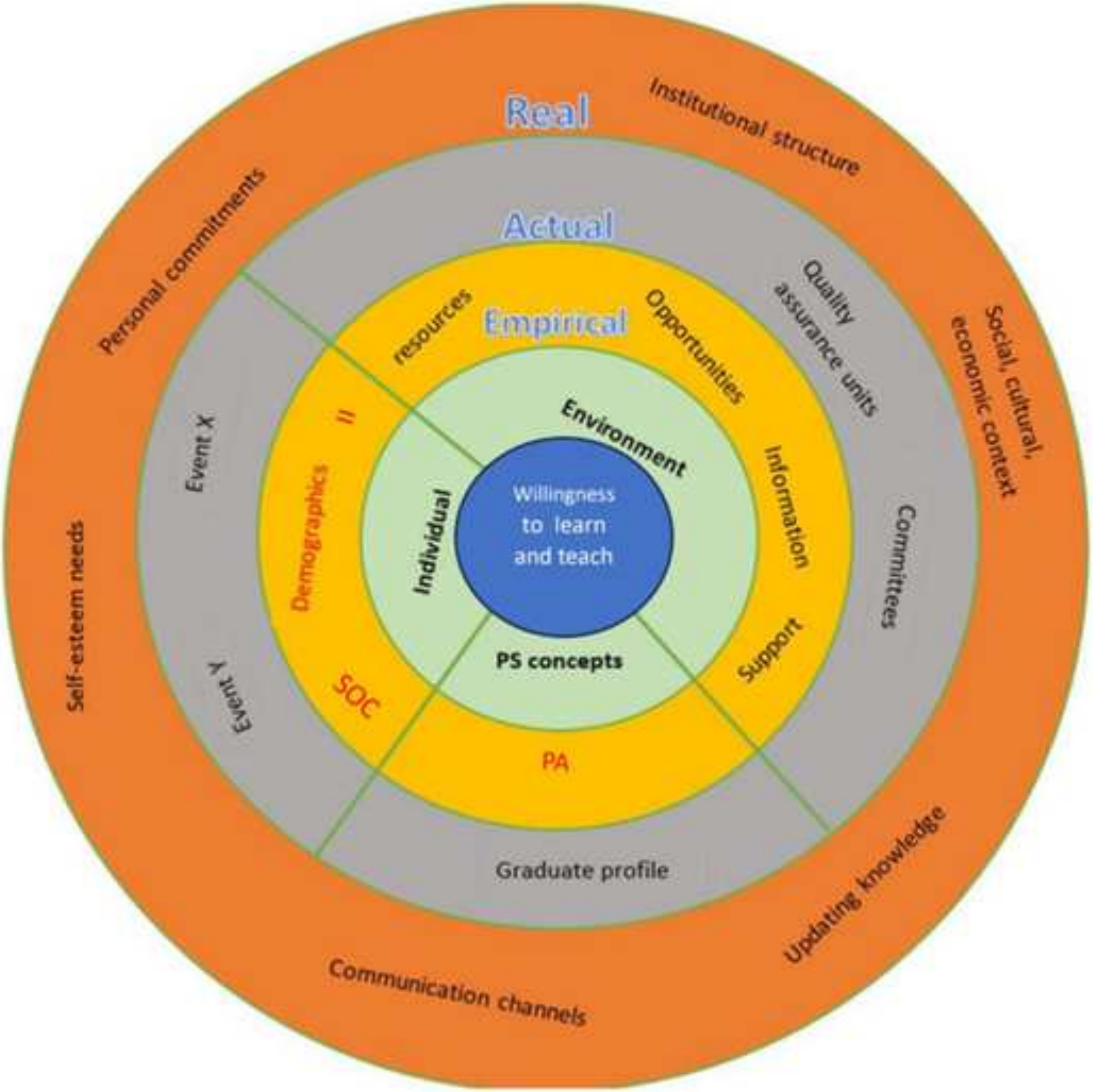

Figure 2. Conceptual framework[1]

PS- Patient safety PA-Perceived Attributes II- Individual Innovativeness

[1] In the empirical domain, extensive methods are in red font, and intensive methods are in black. The conceptual framework will only represent the anticipated events and mechanisms as examples.

S2 File. Curricula data extraction sheet

Click here to access/download

Supporting Information

S2 File. Curricula data extraction sheet.doc

S4. Figure SoC quick scoring device

Click here to access/download
Supporting Information
S4. Figure SoC quick scoring device.doc.tif

S4. Figure SoC quick scoring device

Click here to access/download
Supporting Information
S4. Figure SoC quick scoring device.doc (1).tif

S1 File. Interview protocol

Click here to access/download
Supporting Information
S1 File. Interview protocol.doc

Click here to access/download
Supporting Information
S3 File. Individual Innovativeness Scale score calculation.docx

A critical realist analysis of nursing educators' willingness to learn and teach ~~concerns on~~ patient safety ~~education~~ in Sri Lanka: study protocol

Dissanayake, DMAP [1, 2], Warnakulasuriya, SSP [3]

1. Dept. of Nursing and Midwifery, Faculty of Allied Health Sciences, University of Sri Jayewardenepura, Sri Lanka
2. Faculty of Graduate studies, University of Colombo, Sri Lanka.
3. Faculty of Nursing, University of Colombo, Sri Lanka

Competing interests- The authors declare that there are no competing interests.

Availability of data and materials –Data will be available upon request when the study is completed and published.

Abstract

Introduction:

Nursing educators' willingness to learn and teach patient safety is identified as a challenge in the process of implementing patient safety education in nursing programmes. ~~concerns regarding patient safety education pose challenges in nursing programs' implementation.~~ However, the literature lacks an in-depth ~~analysis~~ analysis of the factors affecting their willingness. ~~of these concerns' causes.~~ This study aims to identify factors affecting willingness of nursing educators to learn and teach new patient safety concepts. ~~and the structures, generative mechanisms, and~~

~~events leading to differentiations in willingness. different stages of concern among nursing educators regarding patient safety education.~~

~~Study setting:~~ Methods:

~~Study setting:~~The study setting will be the ~~include~~ Diploma and B.Sc. Nursing programs conducted in eight state universities, two private universities approved by the University Grants Commission, and 16 Nurses Training Schools (NTS) affiliated with general and teaching hospitals under the Ministry of Health, Sri Lanka. The sample will consist of permanent nursing educators who are fluent in Sinhala language.~~.~~

~~Study design:~~

The study design ~~will~~ adopts the Critical Realist Mixed Method Sequential Explanatory Design (CRMMSED), based on the critical realistic staged model for explanatory social sciences and Mixed Method Sequential Explanatory design (MMSE). Post conceptualization, the execution of the study will take place in ~~Data collection, analysis, and interpretation will occur in~~ four~~ive~~ ~~phases;~~ phases; 1. Extensive data collection and analysis, 2. Linking Phase, 3. Intensive data collection and analysis and 4. Interpretation phase. ~~Phase 1, Linking Phase, Phase 2, Interpretation phase.~~ ~~:~~ In Phase 1~~1~~ , curriculum content verification , cross-cultural adaptation and validation of the Sinhala version of the Individual Innovativeness scale, ~~s~~ ~~in phase 1, quantitative~~ data collection using ~~through~~ the~~a~~ questionnaire, ~~and curriculum content verification, and~~ identification of educators' stages of concern and Individual innovativeness will

Formatted: Font color: Auto

occur. During t~~T~~he linking phase ~~will involve selecting~~ interviewees will be ~~selected based~~selected based on quantitative data analysis. In phase 3~~2~~, qualitative data will be collected through semi-structured interviews with nursing educators and ~~them~~data will be ~~thematically analyzed~~analysed using deductive thematic analysis. In the interpretation phase, quantitative and qualitative data analyses will be triangulated to understand structures and mechanisms contributing to educators' willingness. ~~The interpretation phase will triangulate quantitative and qualitative data analyses to understand structures and mechanisms contributing to educators' willingness . stages of concern.~~

Data analysis:

~~Validation of the questionnaire~~ SPSS and AMOS will be ~~used to~~used to conduct exploratory and confirmatory factor analysis of the ~~and~~questionnaire. ~~quantitative data analysis~~ Furthermore, associations and correlations of quantitative ~~will~~data will be identified. ~~conducted using SPSS and AMOS, while~~ NVivo will be utilized for processing and thematic analysis of qualitative data. Retroductive inference and causative analysis will identify underlying events, structures, and generative mechanisms.

What is already known about the topic?

- Nurse educators' willingness to learn and teach ~~concern for~~ patient safety has been identified as a contributing factor to the success of integrating patient safety concepts into existing curricula.
- According to the Concerns Based Adoption Model (CBAM), educators' concerns about education innovation comprises ~~fall into~~ seven categories. Stages are ranging from "unconcerned" to "informational", "personal", "management", "consequence" "collaboration" and "refocusing".
- Individual innovativeness is defined as the "willingness of an individual to change". Previous studies have found a positive relationship between individual innovativeness and education technology adoption behaviour.

What does this paper add?

- With this protocol, attempts will be made to identify individual, environmental, and innovation-related factors which contribute to the emergence of differentiations in willingness ~~in different concerns of nursing educators, with regard~~ to learni~~ng~~ and teach~~ing~~ ~~the integration of~~ patient safety concepts among nursing educators.
- Using this protocol, it will be proposed to explore the possibility of synergising mixed methods research and critical realism, which enables the researcher to identify varied categories of the study population through quantitative data and to identify the causative mechanisms of those categories through qualitative data.

Key words: Curriculum; Nursing Education; Patient Safety; Qualitative Research; Teaching; Validation study

Introduction

Patient safety (PS) has been identified as an emerging trend in health services, and the World Health Organization (WHO) advocates for its inclusion in education accreditation (1). According to the WHO, PS refers to a framework of organized activities that promote cultures, processes, procedures, behaviours, technologies, and environments in healthcare that consistently and sustainably lower risks, reduce the occurrence of avoidable harm, make errors less likely, and reduce their impact when they do occur (2). In the context of ensuring patient safety, the emphasis has been shifted from assigning individual blame to recognizing and addressing the impact of human factors and fallibility. It is essential that these new concepts are effectively transmitted to the next generation of healthcare professionals.

Education on PS has been identified as a proactive step towards safer care and there is a global agreement on PS in undergraduate healthcare education (3,4)~~(3,4)~~. Numerous PS frameworks have been developed and integrated into nursing programmes worldwide, such as the Quality and Safety Education for Nurses (QSEN) in the USA (5), Australian National Patient Safety Education Framework (6), South Korean Patient Safety Competency Framework (PSCF) (7) and Learning Outcomes for Patient Safety in Undergraduate Nursing Curricula in Canada (8). WHO-Multi Professional Patient Safety Curriculum is also based on the Australian framework.

However, curriculum implementers have reported the challenging nature of integrating PS into curricula. Limited understanding of the PS concepts (9,10) and reducing PS practices to just one aspect, such as focusing solely on reducing medication errors (11), can hinder its overall impact. Furthermore, accommodating PS into an already saturated nursing curriculum (12,13) ,and not recognizing its importance in clinical practice (4,11,14) pose additional challenges.

One of the prominent challenges in integrating PS into nursing curricula is the willingness of faculty to learn and teach these concepts (4,15,16). Several other issues related to faculty, such as a lack of recognition of the importance of PS by the faculty and a lack of confidence in teaching these concepts, have also been identified as challenging (4,17,18) . Nursing educators must be willing to integrate and teach new PS concepts to produce nurses who can champion PS. Therefore, further research is needed to evaluate both the curricula and nurse educators' concerns about teaching willingness to learn and teach these concepts.

There has been very limited understanding on the current level of patient safety education in Sri Lanka. Results of this study will generate new knowledge on how patient safety has been perceived by Sri Lankan nursing education system. It will also provide an understanding of possibilities of integrating internationally recognized patient safety competencies into local nursing curricula.

Formatted: Font: 12 pt

Formatted: Justified

Patient safety education in the South Asian region and Sri Lanka

A few studies have been conducted in the Southeast Asian region to assess the perceptions of PS, barriers to implementing PS and strategies to overcome these barriers. Pelzang et al.,(9) have emphasized the need to integrate and provide PS training and education to all categories of healthcare professionals. The lack of resources is the main barrier to promoting patient safety and quality practices in India (19,20).

In 2016 adverse events reporting forms were introduced to state-sector hospitals in Sri Lanka. In 2019 out of 10324 adverse events reported 30.46% are falls (21). Even though non-clinical events are reported more, it can only be predicted that actual numbers are higher (21,22). A study done in a teaching hospital in Sri Lanka revealed that nursing officers who are competent in skills regard to adverse events and, near misses were 6.35% (23). Nearly half (46.4%) of student nurses have agreed that making errors is inevitable while a considerable proportion (38%) agreed on blaming and punishing people who commit errors (24). These findings emphasise the dire need for improving PS education for nurses in Sri Lanka, particularly in adverse event reporting.

The "WHO South East Asia Regional Strategy for patient safety (2016-2025)" has proposed for integrating PS principles and practices into all professional healthcare courses, including nursing (25). This implies that Sri Lanka has strategically agreed to incorporate PS education in undergraduate nursing education. These new perspectives and obligations make PS education an innovative and emerging necessity, particularly for a developing country like Sri Lanka.

World Health Organizations’ Multi-Professional Patient Safety Curriculum (WHO-MPSC) (26) provides a common foundation for PS education. The curriculum contains a wide array of topics on patient safety, human factors, complex systems, teamwork, learning from error, clinical risk management, quality improvements, engaging patients and carers, infection control, surgical safety and medication safety. "It contains information for all levels of faculty and staff, and it lays the foundation for capacity building in essential patient safety principles and concepts” (27). The guide has been written to be applicable to different cultures and contexts using easily-understood language (27). Ginsberg et al (12) have found that low and middle-income countries are slow to progress towards integrating the aforesaid curriculum. They have examined the potential barriers pertaining to; a) curriculum content, b) context and c) implementation process. Insufficient training to enable faculty, lack of faculty enthusiasm/meaningful participation and lack of faculty cooperation to address implementation challenges were among the top three potential barriers to the implementation process. Therefore, the concerns of nursing faculty should be identified and addressed to ensure seamless integration of PS concepts into existing curricula.

Factors that affect willingness to learn and teach a new concept

~~Concerns-Based Adoption Model (CBAM)~~

Individual factors

Formatted: Font: 14 pt

Formatted: Heading 3, Line spacing: Double

Stages of Concern

Formatted: Heading 7, Left

The Concerns-Based Adoption Model (CBAM) (28) describes how teachers' concerns over the acceptance and adoption of educational change vary. Seven Stages of Concern (SoC) about an education innovation have been identified in which individuals progressed from implementing innovation to becoming competent in using it (Table 1).

Table 1: Typical expressions of concern about an innovation (28)

Stages of Concern			Expressions of Concern
"Impact"	6	Refocusing	I have some ideas about something that would work even better.
	5	Collaboration	I would like to coordinate my efforts with others, to maximize the innovation's effect.
	4	Consequence	How is my use affecting my students?
"Task"	3	Personal	I seem to be spending all my time getting my materials ready.
"Self"	2	Management	How will using it affect me?
	1	Informational	I would like to know more about it.
"Unconcerned"	0	Unconcerned	I am not concerned about it.

A 35-item Stages of Concern questionnaire is available in order to determine where someone is in the Stages of Concern range (28). Although it may appear that progression through the stages of concern is a linear process, it is important to note that acceptance and adoption of an innovation are highly influenced by the intrinsic factors of an individual. Even as individuals gain more knowledge and experience with innovation, there is no guarantee that their earlier concerns will be resolved (28).

The authors of “Measuring implementation in schools: the stages of concern questionnaire”, George et al (28) noted that the development of higher-level concerns depends on the **individual's perceptions**, **the environmental context** and **the innovation** itself. Buabeng-Andoh (29) identified personal, institutional, and technological factors that encourage the use of computer technology in teaching and learning, such as ICT competence, computer self-efficacy, professional development, and technical support. Lai and Chen (30) also suggested similar factors, including individual characteristics such as perceived usefulness and ease of use, school characteristics such as incentives and support, and environmental characteristics such as supervisor and peer influence.

Individual innovativeness~~factors~~

Formatted: Font: 14 pt

Formatted: Heading 7

When introducing an innovative idea, the process of adapting that idea is rarely linear (31). Roger's diffusion of innovation theory explains that an individual's adoption of an innovation progresses through five stages: knowledge, persuasion, decision, implementation, and confirmation (31). Additionally, he suggests that individuals will adopt innovations at different

time frames depending on the adopter category to which they belong (Innovators, Early Adopters, Early Majority, Late Majority, and Laggards) (31).

The general innovativeness of an individual is typically viewed as a psychological construct or individual characteristic. It shapes an individual’s disposition toward newness regardless of the kind of innovation (32). Hurt et al., (33) define Individual Innovativeness as the “willingness of an individual to change”. Previous studies have found a positive relationship between individual innovativeness and education technology adoption behaviour among academicians and education administrators (34,35).

Apart from Individual innovativeness, there are studies done to identify how personal characteristics such as age, gender, and education affect innovation adoption (36–38).

Environmental factors

Nursing educators working environment includes the teaching facility and the hospitals where clinical teaching and practical exams are carried out. Previous studies in nursing have frequently utilized (39–41) Kanter’s ‘Theory of Structural Power in Organizations’ (42) to examine structural empowerment. Structural empowerment refers to workplace conditions and policies that facilitate four dimensions. These dimensions are access to information, support from colleagues, resources such as time and technology, and opportunities for professional development and growth. These dimensions influence the nursing educators' work environment and their ability to provide high-quality education to students. Research done in the management and IT industries has found that positive working environments such as strong managerial support, and allocation of sufficient resources and incentives become crucial catalysts in embracing innovation

(38,43). As such, it could be predicted that nursing educators' environment could affect education innovation adoptions.

Factors related to the innovation

Perceived attributes (PA) are important factors to consider when studying the adoption of an innovation. Relative advantage, compatibility, complexity, observability, and trialability are the five main perceived attributes (31). Relative advantage means how much better an innovation is perceived to be, compared to the idea it replaces. Compatibility refers to how well the innovation fits with the values, experiences, and needs of potential adopters. Complexity explains how difficult the innovation is to understand and use. Observability refers to how visible the results of the innovation are to others. Trialability refers to the ability of individuals to experiment with the innovation before fully adopting it. These perceived attributes have been used in previous research to investigate their effect on the concern of users when adopting an innovation (29,30).

The methodological approach~~y~~

The methodology is concerned with the theoretical and philosophical assumptions about methods (44). It provides the frame of reference for the research which is influenced by the paradigm in which our theoretical perspective is placed or developed (45). Another definition is “the specific theoretical level at which research methods and approaches are conceived, chosen and justified” (46). Choosing a set of methods as opposed to others can be called methodological

justification of the choice of methods (46). Methodology can be objectivist, subjectivist or critical realist.

Objectivist methodology

Objectivist methodology is based on positivism. It claims that reality exists independent of the human mind, and it can be understood only through objective methods of inquiry (47). Objective research methods require quantifiable data about the world to represent reality. When used in social research, objectivist methodology faces debilitating limitations. It may oversimplify complex social phenomena by reducing them to quantifiable variables, potentially overlooking important nuances and contextual factors. This approach may not capture the full richness and complexity of human experiences and social interactions (46).

Subjectivist methodology

Subjectivist methodology is based on interpretivism claims that the social and human world is a human creation based on their subjective encounters with them. There is no objective reality. It emphasizes that research should aim at understanding how people make sense of their own world (46).The limitations of this methodology include relying heavily on individual interpretations, which are influenced by personal biases or social contexts (47). The subjectivist methodology may not provide a comprehensive understanding of complex phenomena as it primarily focuses on individual perspectives and experiences, potentially overlooking broader structural or systemic factors (46).

Critical realistic methodology

Critical realistic methodology tries to transcend the subjectivist and objectivist dichotomy in epistemology and methodology. The triumvirate of critical realism is ontological realism, epistemic relativism and judgemental rationality (48).

Ontological realism

CR posits that reality is intransitive. There is an objective reality in the social world outside the human mind regardless of our ability to understand or decode it. Social reality includes languages, social processes, classes, economies, religion, whereas natural reality includes plants, trees, gravity etc. (48,49).

Epistemic relativism

Human knowledge gets limited by the methods we would use to uncover reality. Therefore, human knowledge is transitive. Our knowledge is finite, contextual and fallible (48,50,51).

Judgemental rationality

Critical realists should evaluate diverse and competing claims about the world before making a judgement. Human knowledge is not just facts or opinions but essentially a rational judgement (48).

Critical realism posits that the world is a stratified open system where structures and mechanisms with causative powers (real domain) generate events, which occur regardless of whether we experience them or not. It suggests that what happens in the world (actual events/Actual domain) is not the same as what is observed/experienced (empirical phenomena/empirical domain). Therefore, critical realism recognizes that we cannot reduce actual events to empirical

phenomena or causative mechanisms to empirical phenomena. Critical realism is a meta-theory. As a meta-theory, critical realism is more an ontology than methodology. It focuses primarily on the nature of reality and does not firmly prescribe how to capture or know that reality (51).

Critical realism is not limited to a specific research method or approach, but instead provides a framework for understanding the underlying reality of the phenomena being studied. This allows researchers to use a variety of methods to study different aspects of the same phenomenon and to integrate findings from different methods to gain a more comprehensive understanding. The focus is on understanding the mechanisms and structures that generate the observed phenomena, rather than on a particular methodology or technique. This flexibility in approach makes critical realism a valuable tool for researchers in a variety of fields who seek to gain a deeper understanding of complex phenomena (49).

In this study, researchers will utilize critical realism's depth reality (52) to overcome the limitations of the empirical nature of the approaches used to examine the stages of concern in earlier studies (53,54). In this study empirical evidence will be obtained on the stages of concerns, in individual, environmental and innovation-related areas, and critical realism’s depth reality will be used to understand complexities that are not obvious and visible (**Figure 1**).

Figure 1. The stratified ontology of critical realism

Critical realists use double hermeneutics, abduction and retroduction to conceptualize the actual events and generative mechanisms. Studying objects of nature involves simple hermeneutics

while studying social objects involves 'double hermeneutics' as the social scientists are 'interpreting other people's interpretations' (49). It is essential to distinguish what needs to be explained and explanations given by others. Charles S. Peirce was the first to report abductive inference in 1932 (55). It is the logical method of discovering or drawing conclusions from, circumstances and structures that are not given in individual empirical data. Retroduction is the mode of inference in which events are explained by postulating (and identifying) mechanisms which are capable of producing them (52,56). This allows the researcher to go beyond empirical findings and critically examine the in-depth reality of a phenomenon.

Aim of the study

To ~~In the present study, the aim is to~~ conduct a critical realist analysis of factors influencing willingness to learn and teach ~~the stages of concern among~~ adverse events related PS concepts by nursing educators. ~~regarding education on adverse events related to PS concepts.~~

~~Hypotheses of Phase 1~~

~~**Hypothesis 1**: The stages of concern (SoC) of nursing educators regarding patient safety education in nursing educators will increase when the individual innovativeness of the educators is high.~~

~~**Hypothesis 2**: The integration of adverse events related to patient safety content in the curricula will increase when the stages of concern in nursing educators are high.~~

Significance of the study

There is very limited understanding on the current level of patient safety education in Sri Lanka. The results of this study will generate new knowledge on how PS has been perceived by Sri Lankan nursing educators. It will also provide an understanding of the possibilities of integrating internationally recognized PS competencies into local nursing curricula in Sri Lanka. Critically analysed willingness~~concerns~~ of nursing educators will contribute to equip education institutions with information on individual, environmental, and PS content-related changes and improvements. In addition, this study will enable curriculum developers to proactively address the concerns of nursing educators, leading to better outcomes. Improved education on PS can minimize the effects of adverse events in healthcare and improve the public image of healthcare institutions. Even though the study will not generate a generalizable theory, the critical realist methodology used in this study will be useful for the future researchers, -when conducting similar studies in any part of the world.

The conceptual framework

As a summation of Phase 1, a -conceptual framework for this study (**Figure 2**) was developed to graphically present the theoretical and methodological underpinnings of the study. Anticipated events, structures and mechanisms were included acknowledging the inevitable alterations, post-data analysis.

Figure 2. The conceptual framework

Objectives

General objectives

1. To identify and examine the factors that influence nursing educators' ~~stages of concern regarding the integration of~~ willingness to learn and teach adverse events related patient safety concepts ~~in their teaching~~.
2. To analyse the underlying structures and generative mechanisms that contribute to the development of variations in willingness to learn and teach patient safety, ~~different stages of concern~~ among nursing educators.

Specific objectives

1. To assess the extent of inclusion of adverse events related patient safety concepts in the curricula of selected nursing programmes.
2. To translate and validate the Sinhala version of the Hurt-Joseph-Cook individual innovativeness questionnaire for use with nursing educators.
3. To evaluate the level of individual innovativeness among nursing educators.
4. To determine the specific stages of concern exhibited by nursing educators regarding the integration of adverse events related patient safety concepts.
5. To explore the correlations between Individual Innovativeness and the stages of concern among nursing educators.
6. To examine the perceived attributes and environmental factors that facilitate or hinder the adoption of adverse events related patient safety concepts by nursing educators.

7. To synthesis~~identify~~ the actual events, generative mechanisms and structures that contribute to the facilitation or hindrance of the adoption of adverse events related patient safety concepts.

~~8. To analyse how variations in willingness to learn and teach PS different stages of concern emerge from the underlying generative mechanisms and structures~~

Materials and methods

In Critical realistic methods, quantitative and qualitative data collection is frequently termed as extensive methods and intensive methods, respectively (49,56). In this study, empirical data collected through extensive methods will be used to explain the phenomenon, while abductive inferencing will be used to identify actual events which are not explicitly linked to the phenomenon. Retroduction will be used to identify structures and generative mechanisms in the real domain.

Study design

Mixed Method Sequential Explanatory (MMSE) (57) design and the staged model for explanatory social sciences (DAARCC) proposed by Danermark (2019) (49), were adopted when designing the research study. DAARCC stands for D- description, A -analytical resolution, A-abduction/theoretical redescription, R- retroduction, C-comparison between different theories and abstractions, C-concretization and contextualization. Baisley (58), has proposed a Critical Realist Mixed-Methods Sequential Explanatory Design (CRMMSED), a research design combining

critical realistic concepts, the staged model for explanatory social sciences and the original mixed-method sequential explanatory design. This research design follows the same design as CRMMSED: extensive data collection and analysis, linking phase, intensive data collection and analysis phase and interpretation phase. Whereas description (D), analytical resolution (A), and abduction/theoretical redescription (A) stages of the DAARCC model recur during conceptualization and data collection (**Table 2**).

Table 2. Comparison of study designs

Sequence in this study	Corresponding CRMMSED phases	Corresponding MMSE phases	Corresponding DAARCC stages
~~1.~~ Conceptualization			D- Description,<br>A -Analytical resolution,<br>A- Abduction/theoretical redescription
~~2.~~ Data collection and analysis — **Phase 1-** extensive data collection and analysis	1. Extensive data collection and analysis	• Quantitative data collection<br>• Quantitative data analysis	D- Description,<br>A -Analytical resolution,<br>A- Abduction/theoretical redescription
**Phase 2-** Linking phase ~~Phase 2~~	2. Linking phase	• Connecting quantitative and qualitative phases	
**Phase 3-**~~3~~ Intensive data collection and analysis	3. Intensive data collection and analysis	• Qualitative data collection<br>• Qualitative data analysis	
**Phase 4-** ~~3.~~ Interpretation ~~Phase 4~~	4. Interpretation phase	• Triangulation~~Integration~~ of the qualitative and quantitative results	R- Retroduction,<br>C-Comparison between different theories and abstractions,<br>C-Concretization and contextualization

Formatted: Font color: Accent 5
Formatted Table
Formatted: No bullets or numbering
Formatted: Font: Bold
Formatted: Indent: Left: 0"
Formatted: No bullets or numbering
Formatted: Indent: Left: 0.24", No bullets or numbering
Formatted: Font: Bold
Formatted: No bullets or numbering
Formatted: Indent: Left: 0.5", No bullets or
Formatted: Indent: Left: 0"
Formatted: Font: Bold
Formatted: Indent: Left: 0"
Formatted: No bullets or numbering
Formatted: Font color: Accent 5
Formatted: Font: Bold
Formatted: No bullets or numbering
Formatted: Font: Bold, Font color: Accent 5
Formatted: No bullets or numbering

~~Sampling technique~~

Study setting

Formatted: Heading 2, Left

The study will be carried out in Nursing Diploma and B.Sc. Nursing programmes that are conducted in state universities (n=8), private universities approved by the University Grants Commission, Sri Lanka (n=2) and Nurses Training Schools (NTS) attached to general and teaching hospitals (n=16) under the Ministry of Health, Sri Lanka.

Study population

Formatted: Heading 2, Left

Permanent nursing educators (N_{total}=413) who are capable of reading, speaking and comprehending the Sinhala language (N_S≈378). ~~and who teach at least one nursing subject (clinical nursing subjects, fundamentals of nursing, management in nursing, trends and issues in nursing etc), whom will have the potential to provide feedback on nursing curriculum, will be selected for the sample.~~

Inclusion and exclusion criteria

Formatted: Space Before: 0 pt

Inclusion criteria:

Formatted: Font: 12 pt

Formatted: Line spacing: Double

Permanent academic staff members whose basic qualification is Nursing will be included in the study. Participant should teach at least one core nursing subject (ex. Fundamentals of Nursing, Clinical Nursing subjects, Management in Nursing, etc.) and capable of reading, speaking and comprehending Sinhala language.

Formatted: Font: 12 pt

Exclusion criteria:

Temporary academic staff will be excluded from the study. Academics who are teaching non-nursing subjects (ex. Anatomy, Physiology, Biochemistry, English, etc.) and those who does not have nursing as their basic qualification will also be excluded.

Sampling technique

The sampling technique for this study involves purposive sampling and stratified random sampling. In **phase 1**, all available curricula in selected institutions will be included in content verification using a purposive sampling technique.

For the self-administered questionnaire, two sets (sample 1 and sample 2) of nursing academics will be recruited. The first set (sample 1) will be used to perform exploratory factor analysis (EFA) of II Scale and will be selected using stratified random sampling. Data from sample 2 will be used to questionnaire survey analysis.

The sample size will be determined based on the rule of thumb of 5-10 subjects per item in instrument validation studies (59) resulting in a sample size of 105 (20 items in II scale, 5:1 subject to variable ratio and adding 5% non-response rate). The participantse educators will be selected from three types of institutions; s (state universities (SU), pprivate universities (PU), and training schools (NTS)) using stratifiedusing stratified random random sampling.

The second set (sample 2) will be used for the confirmatory factor analysis (CFA). This sample and that will consist of 185 participantseducators who were selected using stratified random sampling and not included in sample 1.

Sample calculation:

Formatted: Font: 12 pt

Formatted: Heading 2, Left

Formatted: Font: Bold

TOut of a total population of of 413 nursing academics are 413 (SU=99, PU=17, NTS=297). , To leverage exclusions due to Sinhala language proficiency 35 (SU=10, PU= 5, NTS=20) were exempted. leveraging exclusions due to Sinhala language proficiency, leaving Aapproximately 378 were left as the population of nursing educators fluent in Sinhala.

Sample 1 (n_1) for EFA of II Scale (20 items; 1 item: 5 educators)

n_1= 100+non respondents 5% = 105

Subsampling of n_1 using stratifies random method:

n_1 (SU) = 89/378 X 105 =25

n_1 (PU) = 12/378 X 105 =3

n_1 (NTS) = 277/378 X 105 =77

Remaining population for sample 2 (n_2) calculation = 378-105 = 273

Sample 2 for CFA and questionnaire survey

Calculation 1: Using Yamane's formula (60,61) n = N / (1 + N(e)*2), for a population of 273 with a margin of error of 5%, the sample is calculated to be 162. A sample size of =162+ non respondents 5%= 170 results after adding 5% non-respondents.

Calculation 2: Considering factor analysis for the SoC questionnaire, as subsequent validation is recommended by Tsang et al (62) (35 items; 1 item: 5 educators), 175 (35 items; 1 item: 5 participants) educators are required to calculate CFA. A sample size will be 18445 results after adding 5% as non-respondents for CFA of SoC questionnaire.

Since calculation 2 results a higher number 184 (n_2) was selected as the sample size.

Formatted: Subscript

Formatted: Subscript

Formatted: Subscript

Formatted: Subscript

Formatted: Subscript

Formatted: Subscript

Formatted: Subscript

Formatted: Subscript

Subsampling of n_2 using stratifies random method:

n_2 (SU) = (89-25)/273 X 184 =43

n_2 (PU) = (12-3)/273 X 184 =6

n_2 (NTS) = (277-77)/273 X 184 =135

In **phase 2**, semi-structured interviews will be conducted with ~~12-14~~ nursing educators until data saturation is reached.~~.~~ ~~The actual number will depend on the data saturation.~~ Participants ~~-~~will be selected purposive sampling method~~ly~~.

Formatted: Subscript

Formatted: Font: Bold

Study instruments

Self-administered questionnaire

There will be an online questionnaire consisting of three (3) parts; personal characteristics, Stages of Concern Questionnaire (SoC) questionnaire and Individual Innovativeness (II) scale.

Personal characteristics

Details on gender, age, religion, ethnicity, personal income, marital status, education and employment (university, academic/ admin grade, years of teaching experience, years of clinical experience) will be ~~ascertained in~~ascertained in this part.

Stages of Concern Questionnaire (SoCQ)

The original SoCQ had been tested for reliability (test/retest reliability ranges from 0.65– 0.68) and validity (alpha-coefficients range from 0.64–0.83) (28,63). SoCQ's Sinhala and Tamil

translations (35 items) have been validated in Sri Lanka, and Cronbach alpha of the Sinhala version (mean) was reported to be 0.78 indicating good internal consistency. Content validity has been evaluated by ten (10) language and content experts (64). Psychometric properties of a Turkish translation has been shown to have weak internal consistency and model fit (65). The reliability for four of the seven stages was low (<0.70), and the inter-correlational matrix did not provide support for the hypothesized relationships among the stages. A shortened (16-item) version has been suggested to increase reliability (0.66- 0.82) and validity. A validation study in Hong Kong (66), has found that all the fit indices were not satisfactory and the correlations among some factors were too high. They have suggested a 5-stage model with 22 items. The item-total correlations of the 22 items ranged from 0.46 to 0.70 and the alphas of the five scales varied from 0.75 to 0.84. During this study construct validity of all 3 models will be evaluated via Confirmatory Factor Analysis (CFA) to identify the best model fit in Sri Lankan context.

The validated SoC questionnaire will be used along with the change of "name of the innovation" from "5E instructional method" to "adverse events related patient safety concepts (AER-PS)".

Individual Innovativeness (II) scale

This scale has been developed to measure an individual's orientation towards innovation (33). This has shown strong psychometric characteristics; Cronbach α above 0.80 and it has been used as a valid measure of general innovativeness in earlier studies (67–69). A study done in Pakistan found reliability to be above 0.75 and values of model fit were close to 0.90 (70).

The scale has 20 items and it ranges from options Strongly Disagree = 1; Disagree = 2; Neutral = 3; Agree = 4; Strongly Agree = 5 respectively to rate each item.

Scores above 80 are classified as Innovators.

Scores between 69 and 80 are classified as Early Adopters.

Scores between 57 and 68 are classified as Early Majority.

Scores between 46 and 56 are classified as Late Majority.

Scores below 46 are classified as Laggards/Traditionalists.

Translation of II scale to Sinhala language, cross-cultural adaptation and psychometric analysis will be done following the established guidelines on cross-cultural adaptation (71) and psychometric analysis (72,73).

Data collection

Data collection is not anonymised. In order to protect privacy and confidentiality; names and university affiliations will be replaced with codes during data processing. Any data requested during publications will also be anonymised. Data will be stored in password-protected documents on a secure Google Drive linked to the researcher's faculty email address and later data will be transferred to an external storage device. After 10 years from the submission of the thesis, the data will be deleted. Only the investigator and supervisor will have access to non-anonymised data.

Phase 11: Extensive/ quantitative procedures

I. Curriculum content verification.

This will be used as a way of fact-checking/validating the adverse events related (AER) content of PS is being taught. Soft copies of the curricula will be requested from deans of the faculties and the Director-Nursing Education, Ministry of Health, Sri Lanka. Upon receiving, a word search of the relevant PS content will be carried out. A Microsoft Excel matrix will be used to document which AER-PS concepts are mentioned in curricula.

II. Self-administered questionnaire

Authorization will be obtained from the relevant deans of faculties and training schools' principles to collect data from their academic staff members and to verify the curricula content. A random sample of nursing educators from each institution category (state universities, private universities and training schools) will be selected. An online questionnaire link will be emailed or texted to selected nursing educators. The selection of participants will not be based on ethnicity. An information sheet will be provided to participants and will contain a brief description of the study, ethical approval, details of investigators, the procedure for asking questions, and participants' rights. Prior to accessing the questionnaire, a consent form will be provided for participants to acknowledge their voluntary participation and the option to withdraw their consent at any time. Non-respondents will receive a reminder on the following week and a phone reminder on the 2nd week.

Formatted: Indent: Left: 0.06"

Phase 33: Intensive/ qualitative procedures

Semi-structured interviews will be conducted to identify perceived attributes of selected PS concepts and environmental attributes which facilitate or inhibit the adoption of selected concepts. One-to-one interviewing of the selected participants will be carried out in person or virtually according to the participant's preference. If done in person, it will be at the participants' academic institution. An interview guide developed by the principal investigator will be used (**S1 File**). Participants will be requested to go through the information sheet and provide consent form prior the interview. Interview will be conducted in participants preferred language (Sinhala, Tamil or English). A Tamil-Sinhala-English translator will be available during the interview for assistance upon permission from the participant. For audio recordings, participants will be advised not to identify themselves or third parties. Digital recordings of the interviews and transcripts will be password protected and saved on a secure Google Drive linked to the researcher's faculty email address. The data will later be transferred to an external storage device once the analysis is completed. Transcribing will be done by the researcher. All transcripts will identify interviewees by codes. Participants will be allowed to review the transcription and provide written consent to use the recording.

Secondary data (policies, news reports, press releases) will be analysed to identify any significant events. Online resources will be retrieved using the keywords "Sri Lanka", "Patient safety", "policies", "news reports", and "press releases" in internet search engines.

Data analysis

Phase 1~~1~~: Extensive data~~methods~~

Curriculum content verification

percentages will be calculated on the inclusion of five selected concepts in each curriculum. The selected adverse events related to patient safety concepts are, 1) the definition of adverse events (AE) in healthcare, 2) Adverse event reporting, 3) human factors in healthcare safety, 4) system factors in healthcare safety and 5) root cause analysis (RCA).

Students' knowledge outcomes mentioned in WHO-MPSC relevant to the above concepts (total 11) and introduction to incident reporting form provided by the Directorate of Healthcare Quality and Safety, Sri Lanka will be checked against the local curricula of each participating institution (**S2 File**). Observations will be marked under 3 categories "Not mentioned", "Partially mentioned" and "Explicitly mentioned". Percentages for each category will be calculated in all participating institutes.

Self- administered questionnaire- personal characteristics, SoCQ and II scale.

Psychometric properties of II scales' Sinhala version will be assessed. Five (5) experts will be consulted to comment on the content validity of the scale. Content Validity Index (CVI) will be calculated to assess the relevancy and clarity of items. Statistical analysis will be conducted using

IBM SPSS Statistics version 20.0 for Windows. Reliability will be assessed using measurements of internal consistency (Cronbach's alpha coefficient and Composite reliability). Exploratory factor analysis (EFA) will be conducted to identify the constructs of the instrument using Principal Component Analysis (PCA), Varimax rotation and Kaiser .Normalization. Construct validity will be evaluated using convergent and divergent validity and confirmatory factor analysis (CFA). Thereafter, -Iindividual innovativeness categories of nursing educators will be identified using the II scoring matrix (**S3. File**).

The validity and reliability of the adapted SoCQ Sinhala version will be assessed. SoC quick scoring device (**S4. Fig.**) will be used to calculate the scores of individual nursing educators and determine stages of concern in each. Frequency, mean, SD and Percentiles will also be calculated.

Phase 32: Linking phase

Correlations of curricula content, personal characteristics, II scale and SoCQ will be assessed using SPSS and AMOS. The results will be used to determine how Individual innovativeness and personal characteristics affect SoC. An attempt will be made to determine if the SoC of nursing educators affect the integration of AER- PS content in nursing curricula. Actual events will be identified and re-described using data collected in Phase 1 and the conceptual framework.

Phase 43: Intensive datamethods

Thematic analysis will be used to analyse transcriptions of semi-structured interviews. Themes will be identified using NVivo. Deductive thematic analysis will be conducted, based on the

conceptual framework presented. Yet, the researchers will not get limited by the framework and will also include inductive analysis, given that new constructs could appear during the analysis phase.

Content analysis will be used to analyse secondary data (policies, news reports, and press releases). Characteristics of the document's content will be described by examining who says what, to whom, and with what effect (74).

~~Phase 4: ~~Interpretation of data ~~phase~~

To comprehend the factors that impact nursing educators' willingness to learn and teach new PS concepts~~stages of concern~~, both quantitative and qualitative analyses will be triangulated (75). An attempt will be made at identifying how quantitative correlations (personal characteristics, SoC score, II score, curriculum content) and qualitative themes generated (on perceived attributes of patient safety education, resources, opportunities, information and support) would integrate to provide an informative, layered explanation of why nursing educators' concerns have become what they are. Retroductive approach causal analysis (56) will be used to identify individual, environmental and curricula-related causes which could have contributed to~~-~~ the willingness to learn and teach~~each stage of concern~~.

Ethical Considerations and Declarations

Ethical clearance to conduct this study has been obtained by the Ethical Review Committee at the Faculty of Graduate Studies, University of Colombo (FGS/ERC/2022/013).

Formatted: Font: (Default) Calibri, 16 pt

Formatted: Heading 2

Formatted: Font: 16 pt

Formatted: Font: (Default) Calibri, 16 pt

The researchers have obtained necessary permissions from the copyright owners to conduct a translation and validation of the Individual Innovativeness scale, as well as to reuse the Stages of Concern questionnaire and its corresponding Sinhala translation.

Risk-Benefit Assessment

There are no interventions that are used during the study which requires special precautions. Therefore, there are no potential risks to the participants. There are no immediate benefits to the participants either. Social and scientific value of the study are mentioned in significance of the study. Participants will be directed to WHO-Multi Professional Patient Safety Curriculum and other patient safety reading materials at the end of the questionnaire.

Participants rights and consent

An email and/or message will be sent to all nursing educators who have met inclusion criteria requesting to participate in the study. Participants will be recruited through volunteer participation. A brief description of the study, ethical approval, details of investigator and supervisor will be provided in the information sheet hyperlinked to the questionnaire. In 2nd section in google form, there will be five questions including, "have you read the information sheet? Do you agree to take part in this study?" If they select YES for all 5 questions and proceed to the questionnaire, it will be considered as consent given. If they select NO for at least one question, they will exit from the google form without proceeding to the questionnaire. In the information sheet, it will be stated that the participants can withdraw their consent to participate in the study at any point, even after submitting the questionnaire or after participating in

interviews. Their data will be duly removed from the study, if they opt out. Interview participants had the right to ask questions, to withdraw from the study at any time, to withdraw their data, to decline to answer any question, to turn off the recorder. The information sheet will provide contact details of the investigator and the ethical approval committee to register any complaints on the study. Interview participants will have the right to check transcription summaries for accuracy and to receive a summary of the findings of the completed research.

Confidentiality and Privacy

Demographic data such as age, designation, university will be requested to be used in finding out associations and correlation. Names of the participants will be requested in order to contact participants for semi-structured interviews. Name of the participant and university will be replaced with codes when processing data to ensure privacy of the participant. If data are requested during publications, anonymity of the participants will be ensured. Security of data will be ensured. Spreadsheets and recordings will be saved in the google drive attached to the researcher's faculty e-mail address provided by University of Sri Jayewardenepura with password protected access only permitted to the researcher. Data will be transported from google drive to an external data storage device post thesis submission in order to free cloud storage space. Data will be saved in password protected documents and will be deleted 10 years after submission of thesis. Only the principal investigator and supervisor will have the access to the data.

Fair participant selection and vulnerability

Nursing academics who fulfil the inclusion criteria are selected regardless of ethnicity for the online questionnaire. However, due to the lack of Tamil language proficiency of the researcher, if there will be any interviews with Tamil speaking participants, a Tamil-Sinhala-English translator will be available during the interview for assistance upon permission from the participant. If the Tamil speaking participant is fluent in Sinhala and or English, interview will be conducted in participants preferred language (Sinhala/English).

Discussion

This study aims to identify the concerns of nursing educators in adopting adverse events related to PS concepts, as well as the factors contributing to these concerns. The study will also explore the impact of individual innovativeness on these concerns. The findings of this study will provide valuable insight into the factors that influence nursing educators' concerns, which can be used to propose changes and improvements to individual, environmental, and PS content. Ultimately, improved education on PS will benefit patients by improving their experiences and outcomes at healthcare facilities.

Previous studies have identified the impact of nursing educators' concerns or perspectives in PS education (76–79), but few have delved into the specific reasons behind these concerns. It is important to address any underlying causes of these concerns to ensure full participation and commitment from nursing educators. However, in a complex society where causation is often multifaceted and debatable, it can be challenging to identify the exact causes of a phenomenon.

Critical realism acknowledges this complexity and proposes the emergence of actual events from generative mechanisms and structures.

While the primary focus is on Sri Lanka, the study's insights and methodological approach have broader applicability to similar studies worldwide. By understanding the underlying mechanisms shaping nursing education in Sri Lanka, the research aims to provide context-specific suggestions, while also contributing to the broader knowledge base in education research.

The researchers recognize that critical realism is a topic of ongoing debate and that some may have reservations about its practical applications, particularly in the three domains of reality (real, actual and empirical) (80). Additionally, there is limited research on healthcare education utilizing the staged model for explanatory social sciences or proposed Critical Realist Mixed-Methods Sequential Explanatory Design. Conducting a study that combines healthcare education, critical realism, innovativeness, and stages of concern is a challenging and interdisciplinary undertaking. The researchers are aware of these challenges and will strive to navigate them using the best available scientific reasoning.

The study results will only be generalizable among Sri Lankan nursing educators who are fluent in Sinhala, due to the use of Sinhala translations of study instruments. When a study is conducted in the English language in a country where the population is composed of non-native English speakers, it could significantly alter the outcome (62,71,81,82). There is a possibility that the level of English proficiency of the participants affects the quality, nuances, and richness of the responses they provide. In a self-administered questionnaire, it could influence the way they perceive questions and their intentions. Therefore, validating the Individual Innovativeness scale in local languages was a necessity before using it. However, due to the lack of Tamil-fluent

educators, validating the Tamil translation was not feasible. Hence, only the Sinhala translation will be validated and used in the study.

There is a possibility that those who are not willing to accept a new idea or new PS concepts in particular, will refuse to participate in this study. If so, it won't be possible to identify/synthesis what causes them to deny this new concept.

The study's findings will be disseminated through several channels. Firstly, they will be presented at research conferences and submitted for publication in peer-reviewed scientific journals. Additionally, a summary of the results will be provided to nursing department heads and participating educators. A report of the study's findings will also be shared with the Director of Nursing Education and the Directorate of Healthcare Quality and PS in Sri Lanka. This will ensure that the results of this study are made available to relevant stakeholders and contribute to the wider conversation around PS education in nursing.

Authors' contributions

DMAP contributed to the conceptualization of the study, developing methodology, and manuscript writing and SSP contributed to developing methodology, manuscript reviewing and finalising.

The authors collectively provide permission to publish this manuscript in PLOS One journal.

Acknowledgements

Not applicable

References

1. WHO. Global strategic directions for NURSING AND MIDWIFERY [Internet]. 2021 [cited 2023 Jan 10]. Available from: https://apps.who.int/iris/bitstream/handle/10665/344562/9789240033863-eng.pdf
2. WHO. Patient safety [Internet]. 2022 [cited 2022 Oct 3]. Available from: https://www.who.int/health-topics/patient-safety#tab=tab_1
3. Walton M, Jeffery H, Van Staalduinen S, Klein L, Rothnie I. When should students learn about ethics, professionalism and patient safety? Clin Teach. 2013 Aug;10(4):224–9.
4. Jang H, Lee NJ. Patient safety competency and educational needs of nursing educators in South Korea. PLoS One. 2017 Sep 1;12(9).
5. QSEN [Internet]. [cited 2022 Sep 29]. Available from: https://qsen.org/competencies/
6. The Australian Council for Safety and Quality in Health Care. National Patient Safety Education Framework [Internet]. 2005 [cited 2022 Sep 28]. Available from: http://www.safetyandquality.gov.au/
7. Lee NJ, An JY, Song TM, Jang H, Park SY. Psychometric Evaluation of a Patient Safety Competency Self-Evaluation Tool for Nursing Students. J Nurs Educ. 2014;53(10):550–62.
8. Canadian Patient Safety Institute. The Safety Competencies: Enhancing Patient Safety

Across the Health Professions. [Internet]. 2nd ed. Edmonton, Alberta; 2020 [cited 2022 Sep 28]. Available from: www.patientsafetyinstitute.ca

9. Pelzang R, Hutchinson AM. How Is Patient Safety Understood by Healthcare Professionals? The Case of Bhutan. J Patient Saf [Internet]. 2017;16(1):106–9. Available from: https://www.ncbi.nlm.nih.gov/pmc/articles/PMC7046141/

10. Usher K, Woods C, Conway J, Lea J, Parker V, Barrett F, et al. Patient safety content and delivery in pre-registration nursing curricula: A national cross-sectional survey study. Nurse Educ Today [Internet]. 2018;66(March):82–9. Available from: https://doi.org/10.1016/j.nedt.2018.04.013

11. Vaismoradi M, Salsali M, Marck P. Patient safety: Nursing students' perspectives and the role of nursing education to provide safe care. Int Nurs Rev. 2011;58(4):434–42.

12. Ginsburg LR, Dhingra-Kumar N, Donaldson LJ. What stage are low-income and middle-income countries (LMICs) at with patient safety curriculum implementation and what are the barriers to implementation? A two-stage cross-sectional study. BMJ Open. 2017;7(6):1–10.

13. Walton MM, Elliott SL. Improving safety and quality: How can education help? Med J Aust [Internet]. 2006;184(10 SUPPL.):S60–4. Available from: http://www.embase.com/search/results?subaction=viewrecord&from=export&id=L43732927

14. Mansour M. Examining patient safety education in pre-registration nursing curriculum: Qualitative study. J Nurs Educ Pract [Internet]. 2013;3:12. Available from: https://doi.org/10.5430/jnep.v3n12p157

15. Sherwood G, Drenkard K. Quality and safety curricula in nursing education: Matching practice realities. Nurs Outlook [Internet]. 2007;55(3):151–5. Available from: https://www.nursingoutlook.org/article/S0029-6554(07)00061-9/fulltext
16. Wong BM, Etchells EE, Kuper A, Levinson W, Shojania KG. Teaching quality improvement and patient safety to trainees: A systematic review. Acad Med [Internet]. 2010;85(9):1425–39. Available from: https://doi.org/10.1097/ACM.0b013e3181e2d0c6
17. Armitage G, Cracknell A, Forrest K, Sandars J. Twelve tips for implementing a patient safety curriculum in an undergraduate programme in medicine. Med Teach [Internet]. 2011;33(7):535–40. Available from: https://doi.org/10.3109/0142159X.2010.546449
18. Walton M, Woodward H, van Staalduinen S, Lemer C, Greaves F, Noble D, et al. Republished paper: The WHO patient safety curriculum guide for medical schools. Postgrad Med J [Internet]. 2011;87(1026):317–21. Available from: https://doi.org/10.1136/pgmj.2009.036970rep
19. Landefeld J, Sivaraman R, Arora N. Barriers to Improving Patient Safety in India: Focus Groups with Providers in the Southern State of Kerala. Indian J Community Med [Internet]. 2015;40(2):116. Available from: https://www.ncbi.nlm.nih.gov/pmc/articles/PMC4389498/
20. Madhok R, Vaid S, Carson-Stevens A, Panesar S, Mathew J, Roy N, et al. Promoting patient safety in India: Situational analysis and the way forward. Natl Med J India [Internet]. 2014;27(4):217–23. Available from: http://www.nmji.in/archives/Volume-27/Issue-4/Medicine-and-Socity.pdf%0Ahttp://ovidsp.ovid.com/ovidweb.cgi?T=JS&PAGE=reference&D=emed15&

NEWS=N&AN=602220479

21. Mallawarachchi SMNSM, Dharmarathna GS. A systematic assessment of adverse event reporting in selected state hospitals in Sri Lanka. J Surg Med [Internet]. 2022 Apr 1 [cited 2022 Sep 28];6(4):494–7. Available from: https://ojs.selsistem.com.tr/doi/10.28982/josam.897752

22. Mendis S. Medical error and negligence. Isl [Internet]. 2016 Apr;12. Available from: http://island.lk/index.php?page_cat=article-details&page=article-details&code_title=143690%3E

23. Wijemanne WMUS, Sridharan S, Gamini LPS. Strengthening of Knowledge and Skills on Patient Safety of Nursing Officers' in a Teaching Hospital in Sri-Lanka. 2019 Dec 1 [cited 2022 Sep 28];1(2):75. Available from: https://sljms.sljol.info/articles/abstract/10.4038/sljms.v1i2.50/

24. Piyarathna JG, Seneviratne SMKS. Knowledge and attitudes towards patient safety among nursing students at three state universities in Sri Lanka [Abstract]. In: Peradeniya University International Research Sessions, Sri Lanka [Internet]. 2016. p. 2016. Available from: http://dr.lib.sjp.ac.lk/handle/123456789/6265

25. WHO SEARO. Regional strategy for patient safety in the WHO South-East Asia Region. 2014.

26. WHO. WHO Patient safety curriculum guide: multi-professional edition [Internet]. World Health Organization, editor. malta: WHO; 2011. 272 p. Available from: https://www.who.int/publications/i/item/9789241501958

27. Farley D, Zheng H, Rousi E, Leotsakos A. Evaluation of the WHO Multi - Professional

Patient Safety Curriculum Guide. 2013; Available from: http://www.who.int/patientsafety/education/curriculum/EN_PSP_Curriculum_Evaluation/en/

28. George AA, Hall GE, Stiegelbauer SM, Southwest Educational Development Laboratory. Measuring implementation in schools : the stages of concern questionnaire [Internet]. Austin, TX: SEDL. Available from; 2006. 98 p. Available from: http://www.sedl.org/pubs/catalog/items/cbam17.html

29. Buabeng-Andoh C. Factors influencing teachers ’ adoption and integration of information and communication technology into teaching: A review of the literature. Int J Educ Dev Using Inf Commun Technol [Internet]. 2012;8(1):136–55. Available from: https://files.eric.ed.gov/fulltext/EJ1084227.pdf

30. Lai HM, Chen CP. Factors influencing secondary school teachers’ adoption of teaching blogs. Comput Educ [Internet]. 2011;56(4):948–60. Available from: https://www.sciencedirect.com/science/article/abs/pii/S0360131510003313

31. Rogers EM. Diffusion of Innovations, 5th Edition [Internet]. Mexico: Simon and Schuster; 2003 [cited 2022 Sep 30]. 576 p. Available from: https://books.google.com/books/about/Diffusion_of_Innovations_5th_Edition.html?id=9U1K5LjUOwEC

32. Aldahdouh TZ, Nokelainen P, Korhonen V. Innovativeness of staff in higher education do implicit theories and goal orientations matter? Int J High Educ [Internet]. 2018 [cited 2022 Sep 30];7(2):43–57. Available from: https://files.eric.ed.gov/fulltext/EJ1172498.pdf

33. Hurt HT, Joseph K, Cook CD. Scales for the Measurement of Innovativeness. Hum

Commun Res [Internet]. 1977;4(1):58–65. Available from: https://academic.oup.com/hcr/article-abstract/4/1/58/4626783?redirectedFrom=fulltext

34. Coklar AN. Individual Innovativeness Levels of Educational Administrators Individual Innovativeness Levels of Educational Administrators Individual Innovativeness Levels of Educational Administrators. Digit Educ Rev [Internet]. 2012 [cited 2023 Mar 20]; Available from: http://greav.ub.edu/der/

35. Akgün F. Investigation of Instructional Technology Acceptance and Individual Innovativeness of Academicians. Turkish Online J Qual Inq [Internet]. 2017 Jul 30 [cited 2023 Mar 20];8(3):291–322. Available from: https://dergipark.org.tr/en/pub/tojqi/issue/30703/292135

36. Arts JWC, Frambach RT, Bijmolt THA. Generalizations on consumer innovation adoption: A meta-analysis on drivers of intention and behavior. Int J Res Mark [Internet]. 2011;28(2):134–44. Available from: http://dx.doi.org/10.1016/j.ijresmar.2010.11.002

37. Bartels J, Reinders MJ. Consumer innovativeness and its correlates: A propositional inventory for future research. J Bus Res [Internet]. 2011 [cited 2022 Sep 30];64:601–9. Available from: https://www.sciencedirect.com/science/article/abs/pii/S0148296310001049

38. Talukder M. Factors affecting the adoption of technological innovation by individual employees: An Australian study. Procedia-Social Behav Sci [Internet]. 2012 [cited 2023 Mar 20];40:52–7. Available from: https://www.sciencedirect.com/science/article/pii/S1877042812006271

39. Laschinger HKS. A theoretical approach to studying work empowerment in nursing: a review of studies testing Kanter's theory of structural power in organizations. Nurs Adm Q. 1996 Dec;20(2):25–41.

40. Manojlovich M. Promoting nurses' self-efficacy: A leadership strategy to improve practice. J Nurs Adm [Internet]. 2005;35(5):271–8. Available from: https://journals.lww.com/jonajournal/Abstract/2005/05000/Promoting_Nurses__Self_efficacy__A_Leadership.11.aspx

41. Knol J, Van Linge R, Tergooiziekenhuizen JK, Van R. Innovative behaviour: The effect of structural and psychological empowerment on nurses. J Adv Nurs [Internet]. 2009 [cited 2022 Sep 30];65(2):359–70. Available from: https://onlinelibrary.wiley.com/doi/10.1111/j.1365-2648.2008.04876.x

42. Kanter RM. Men and women of the corporation: New edition. [Internet]. basic books; 2008 [cited 2022 Sep 30]. 409 p. Available from: https://books.google.com/books/about/Men_and_Women_of_the_Corporation.html?id=B-5GztW4IZQC

43. Frambach R, Schillewaert N. Organizational innovation adoption: A multi-level framework of determinants and opportunities for future research. J Bus Res [Internet]. 2002 [cited 2023 Jul 12]; Available from: https://www.sciencedirect.com/science/article/pii/S0148296300001521

44. Uyangoda J. Writing research proposals in the sosial sciences and humanities : a theoritical and practical guide [Internet]. Social Scientists' Association; 2010 [cited 2022 Sep 30]. 135 p. Available from:

https://books.google.com/books/about/Writing_Research_Proposals_in_the_Sosial.html?id=ko7cjwEACAAJ

45. Walter M. Social research methods [Internet]. 2nd ed. Oxford University Press; 2010 [cited 2023 Jul 27]. 510 p. Available from: https://books.google.com/books/about/Social_Research_Methods.html?id=bdchPwAACAAJ

46. Uyangoda J. Social Research: Philosophical and Methodological Foundations [Internet]. Social Scientists' Association; 2015 [cited 2022 Sep 30]. 657 p. Available from: https://books.google.lk/books/about/Social_Research.html?id=cZfUnQAACAAJ&redir_esc=y

47. Sale JEM, Lohfeld LH, Joseph' S, Brazil K. Revisiting the Quantitative-Qualitative Debate: Implications for Mixed-Methods Research. [cited 2024 Apr 20]; Available from: https://link.springer.com/article/10.1023/A:1014301607592

48. Bhaskar R. Theorising ontology. In: Lawson C, Latsis JS, Martins N, editors. Contributions to Social Ontology: Routledge Studies in Critical Realism [Internet]. Routledge; 2007. p. 192–204. Available from: https://books.google.lk/books?hl=en&lr=&id=ZV8LigP2_xYC&oi=fnd&pg=PA192&ots=LDjgD0jQba&sig=QJKTtV1zeT6UxlEPXomx_xLWQbI&redir_esc=y#v=onepage&q&f=false

49. Danermark B, Ekström M, Karlsson JC. Explaining society: Critical realism in the social sciences [Internet]. 2nd ed. Taylor and Francis; 2019 [cited 2022 Sep 30]. 1–228 p. Available from: https://www.taylorfrancis.com/books/mono/10.4324/9781351017831/explaining-

society-berth-danermark-mats-ekström-jan-ch-karlsson

50. Al-Amoudi. Where constructionism and critical realism converge: interrogating the domain of epistemological relativism. Organ Stud [Internet]. 2011 Jan [cited 2022 Sep 30];32(1):27–46. Available from: https://centaur.reading.ac.uk/24956/2/Al-Amoudi_and_Willmott_2011_centaur.pdf

51. Allana S, Clark A. Applying Meta-Theory to Qualitative and Mixed-Methods Research: A Discussion of Critical Realism and Heart Failure Disease Management Interventions Research. Int J Qual Methods [Internet]. 2018 [cited 2022 Sep 30];17:1–9. Available from: https://us.sagepub.com/en-us/nam/open-access-at-sage

52. Bhaskar R. A Realist Theory of Science (1975). 2nd ed. London and New York: Taylor & Francis e-Library; 2008.

53. Al-Furaih SAA, Al-Awidi HM. Teachers' Change Readiness for the Adoption of Smartphone Technology: Personal Concerns and Technological Competency. Technol Knowl Learn. 2020 Jun 1;25(2):409–32.

54. Geng J, Jong MSY, Chai CS. Hong Kong Teachers' Self-efficacy and Concerns About STEM Education. Asia-Pacific Educ Res [Internet]. 2019 Feb 15 [cited 2023 Jul 17];28(1):35–45. Available from: https://link.springer.com/article/10.1007/s40299-018-0414-1

55. Peirce CS. Collected Papers of Charles Sanders Peirce: Volume II Elements of Logic [Internet]. 1932 [cited 2022 Sep 30]. 962 p. Available from: https://www.hup.harvard.edu/catalog.php?isbn=9780674138001

56. Sayer A. Method in Social Science, A realist approach [Internet]. 2nd ed. London and New York: Routledge; 2010. 336 p. Available from:

https://www.taylorfrancis.com/books/mono/10.4324/9780203850374/method-social-science-andrew-sayer

57. Ivankova N V., Creswell JW, Stick SL. Using Mixed-Methods Sequential Explanatory Design: From Theory to Practice. Field methods [Internet]. 2006 [cited 2022 Oct 1];18(1):3–20. Available from: https://journals.sagepub.com/doi/abs/10.1177/1525822x05282260

58. Gail Baisley H. Pedagogies of presence ; contemplative education across the disciplines in Aotearoa New Zealand [Internet]. Massey University; 2020 [cited 2022 Oct 1]. Available from: https://mro.massey.ac.nz/handle/10179/15782

59. Tabachnick BG, Fidell LS. Using Multivariate Statistics [Internet]. 7th ed. Pearson Education; 2019 [cited 2022 Oct 1]. 815 p. Available from: https://www.pearson.com/en-us/subject-catalog/p/using-multivariate-statistics/P200000003097/9780137526543

60. Yamane T. Statistics: an introductory analysis. 3rd ed. New York: Harper and Row; 1973. 1130 p.

61. Israel GD. Determining sample size [Internet]. 1992 [cited 2023 Jul 18]. Available from: https://www.researchgate.net/profile/Subhash-Basu-3/post/how_could_i_determine_sample_size_for_my_study/attachment/5ebaa4924f9a520001e613b6/AS:890361492811785@1589290130539/download/samplesize1.pdf

62. Tsang S, Royse CF, Terkawi AS. Guidelines for developing, translating, and validating a questionnaire in perioperative and pain medicine [Internet]. Vol. 11, Saudi Journal of Anaesthesia. 2017 [cited 2022 Oct 1]. p. S80–9. Available from: https://www.saudija.org/article.asp?issn=1658-

354X;year=2017;volume=11;issue=5;spage=80;epage=89;aulast=Tsang

63. Hall GE, George AA. Stages of concern about innovation: The concept, initial verification and some implications [Internet]. 1979 [cited 2022 Sep 29]. 38 p. Available from: https://files.eric.ed.gov/fulltext/ED188716.pdf

64. nor Puteh SN, Nawastheen FM. An evaluation on the implementation of 5E instructional model in teaching geography in Sri Lanka. Middle East J Sci Res [Internet]. 2013 [cited 2022 Oct 1];16(5):721–8. Available from: https://www.idosi.org/mejsr/mejsr16(5)13/20.pdf

65. Goktalay SB, Cangur S. Assessing the reliability and validity of the Turkish version of the stages of concern questionnaire. Egit Arastirmalari - Eurasian J Educ Res. 2008;(33):55–72.

66. Cheung D, Hattie J, Ng D. Reexamining the stages of concern questionnaire: A test of alternative models. J Educ Res. 2001;94(4):226–36.

67. Goldsmith RE. The validity of a scale to measure global innovativeness. J Appl Bus Res [Internet]. 1991 [cited 2022 Oct 1];7(2):89–97. Available from: https://clutejournals.com/index.php/JABR/article/view/6249

68. Pallister JG, Foxall GR. Psychometric properties of the Hurt-Joseph-Cook scales for the measurement of innovativeness. Technovation. 1998;18(11):663–75.

69. Aldahdouh TZ, Korhonen V, Nokelainen P. What contributes to individual innovativeness? A multilevel perspective. Int J Innov Stud. 2019 Jun 1;3(2):23–39.

70. Ali I. Personality traits, individual innovativeness and satisfaction with life. J Innov Knowl [Internet]. 2019;4(1):38–46. Available from: https://doi.org/10.1016/j.jik.2017.11.002

71. Beaton DE, Bombardier C, Guillemin F, Ferraz MB. Guidelines for the process of cross-cultural adaptation of self-report measures. Spine (Phila Pa 1976) [Internet]. 2000 [cited 2022 Oct 1];25(24):3186–91. Available from: https://journals.lww.com/spinejournal/Fulltext/2000/12150/Guidelines_for_the_Process_of_Cross_Cultural.14.aspx
72. American Educational Research Association, American Psyhological Association, National Council on Measurement in, Education, Fernández-Ballesteros R. Standard for Educational and Psychological Testing [Internet]. 2008 [cited 2023 Jan 9]. Available from: https://www.apa.org/science/programs/testing/standards
73. David, Streiner, Norman GR, Cairney J. Health Measurement Scales_ a Practical Guide to Their Development and Use-Oxford University Press. Pap Knowl Towar a Media Hist Doc. 2014;
74. Vaismoradi M, Turunen H, Bondas T. Content analysis and thematic analysis: Implications for conducting a qualitative descriptive study [Internet]. Vol. 15, Nursing and Health Sciences. 2013 [cited 2022 Oct 1]. p. 398–405. Available from: https://onlinelibrary.wiley.com/doi/abs/10.1111/nhs.12048
75. Creswell JW, Clark VLP, Gutmann ML, Hanson WE. Advanced Mixed Methods Research Designs. In: Tashakkori A, Teddlie C, editors. Handbook of mixed methods in social & behavioral research. CA: Thousand Oaks,CA: Sage; 2003. p. pp209-240.
76. Ginsburg LR, Dhingra-Kumar N, Donaldson LJ. What stage are low-income and middle-income countries (LMICs) at with patient safety curriculum implementation and what are the barriers to implementation? A two-stage cross-sectional study [Internet].

2017;7(6):1–10. Available from: https://doi.org/10.1136/bmjopen-2017-016110

77. Mbuthia NN, Moleki MM. Perspectives on patient safety education in the Kenyan preregistration nursing curriculum. Afr J Nurs Midwifery [Internet]. 2018;20(2). Available from: https://journals.co.za/doi/pdf/10.25159/2520-5293/3866https://unisapressjournals.co.za/index.php/AJNM/article/download/3866/2882 NS -

78. Wu AW, Busch IM. Patient safety: A new basic science for professional education. GMS J Med Educ. 2019;36(2).

79. Lee SE, Dathinten VS, Do H. Patient safety education in pre-registration nursing programmes in South Korea. Int Nurs Rev [Internet]. 2020;67(4):512–8. Available from: https://onlinelibrary.wiley.com/doi/abs/10.1111/inr.12630 NS -

80. Groff R. critical realism network. 2022 [cited 2023 Jan 19]. There Aren't Really Three Domains: or, Metaphor Is Great, Except When It's Not – Critical Realism Network. Available from: https://criticalrealismnetwork.org/2022/07/21/there-arent-really-three-domains/

81. Guillemin F, Bombardier C, Beaton D. Cross-cultural adaptation of health-related quality of life measures: Literature review and proposed guidelines. J Clin Epidemiol [Internet]. 1993 Dec 1 [cited 2023 Jul 17];46(12):1417–32. Available from: http://www.jclinepi.com/article/089543569390142N/fulltext

82. Gjersing L, Caplehorn JR, Clausen T. Cross-cultural adaptation of research instruments: Language, setting, time and statistical considerations. BMC Med Res Methodol. 2010;10.

Supporting documents

S1. File. Interview Protocol

S2. File. Curricula data extraction sheet

S3. File. Individual Innovativeness Scale score calculation

S4. Fig. SoC quick scoring device

Responses to reviewers’ comments

Dear Editor and reviewers,

Thank you for reviewing our manuscript yet again. We highly appreciate the time and effort you have made to point out the amendments needed.

We would like to express our sincere gratitude for allocating your time to review our protocol critically and thoroughly. We re-evaluated our conceptual framework, after reviewers’ comments on our methodology and the need hypotheses. We could not help but make an adjustment in the conceptual framework and research title as the protocol demanded these changes. We have attached the amended conceptual framework.

We had to change the title from “A critical realist analysis of nursing educators’ **concerns on patient safety** education in Sri Lanka: study protocol” to “A critical realist analysis of nursing educators’ **willingness to learn and teach patient safety** in Sri Lanka: study protocol. In the conceptual framework, Stages of Concern deemed more apt to be under Individual factors and it was changed accordingly. We have made these changes identifiable in the manuscript with track changes. For your kind consideration please.

We have tried our best to provide sufficient amendments and justifications when addressing each of your comments. Please find below, the responses and details of amendments done to the manuscript.

**Reviewer / editor comments**	**Explanation**	**Amendments done (Location in manuscript- without track changes)**
**Reviewer #4:**		
Thanks for your valuable article. it seems that the needs for conducting this research should be more explained in the introduction.	Thanks for your encouraging feedback. We have expanded "significance of the study".	L298-309
**Reviewer #5:**		
Abstract - It is clearer if authors mention "Methods" after introduction instead of setting and design. Under methods, you could describe all the components (setting, study population, inclusion and exclusion criteria, sample size, sampling method, study instruments....).	Thank you for your suggestion. Changed the subtopics. We did not elaborate on the sampling method, and sampling size in the abstract due to the word limit. But these have been explicitly mentioned in the body of the article.	
Under data analysis, what are the statistical tests utilized for analysis?	Factor analysis- EFA and CFA, associations and correlations of questionnaire data were included in the abstract.	L42-44
The study protocol is a lengthy one. contain a validation, audit and descriptive studies which involve quantitative and	This information is provided in Table 2. Comparison of study designs and in Abstract.	L30-32<br>L363

qualitative parts. Better if authors can **<u>clearly mention as stages or phases</u>** under the whole study.		
There are some repetitions which could be avoided and leads to a concise protocol.	We could avoid some repetitions if we rearrange the manuscript according to the Phases (collating sampling, instruments, data collection and analysis together), But then it will deviate from the manuscript guideline of the journal. Nevertheless, minor changes were done to address this.	
As this is not an analytical study, how the authors assess the hypothesis mentioned?	Thank you for highlighting this. We agree to your opinion that this study does not necessarily need hypotheses to execute it or for its' analysis.	removed hypotheses.
In hypothesis 1 - line 341 - repetition of " nursing educators"	removed.	removed.

Specific objectives 6 and 7 are more or less same.	6. To examine the perceived attributes and environmental factors that facilitate or hinder the adoption of adverse events related patient safety concepts by nursing educators.<br>7. To identify the events that contribute to the facilitation or hindrance of the adoption of adverse events related patient safety concepts.<br><br>Perceived attributes and environmental factors are in the empirical domain. Phenomena in empirical domain are observed and experienced.<br><br>Perceived attributes on an innovative concept cannot be equated to actual events.<br>We accept that certain "opportunities" participants mention have the possibility of being identified as actual events. To overcome this: if a participant mentions certain opportunity (eg. training) was helpful in accepting new PS concepts, we will use this finding in empirical domain. We will further inquire to find out what directives/policies or recommendations were there enabling these opportunities. Latter findings will be identified as Actual Events.	7. To identify the **events** that contribute to the facilitation or hindrance of the adoption of adverse events related patient safety concepts.<br>8. To analyse how variations in willingness to learn and teach PS emerge from the underlying generative mechanisms and structures<br><br>To improve clarity we amalgamated the objective on "actual domain" with "real domain" as both objectives are operationalized using causal analysis, retroduction and "synthesis".<br>**7. To synthesis the actual events, generative mechanisms and structures that contribute to the facilitation or hindrance of the adoption of adverse events related patient safety concepts.**

Specific objective 8 - Is this realistic?	In Critical realism, retroduction is the central mode of inference. Retroduction is the way to dive beyond the measurable elements of observations through events to identify the structures, context, and mechanisms that **explain the observation.**<br>Our belief in the existence of a mechanism can be based either on our ability to directly observe it (perceptual criteria), with or without tools to do so, or on our **ability to observe its effects** (causal criteria) (Bhaskar 1975, p. 179).<br>Retroduction allows researchers to identify a set of plausible candidate causal mechanisms. This enables a researcher to investigate the potential causal mechanisms and the conditions under which certain outcomes will or will not be realised.<br>Empirical corroboration allows to selection of the mechanism(s) that offers the best explanation (Mukumbang, 2021).These mechanisms belong to different strata of reality: (i) physical, (ii) biological, (iii) psychological, (iv) psychosocial, socio-economic,(v) cultural, and (vii) normative mechanisms (Danermark, 2019).<br>Some examples of such analysis: Saxena, D. (2019), Wynn Jr, D. E., & Williams, C. K. (2008)	8. To analyse how variations in willingness to learn and teach PS emerge from the underlying generative mechanisms and structures.<br><br>**new 7. To synthesis the actual events, generative mechanisms and structures that contribute to the adoption of adverse events related patient safety concepts.**<br><br>There is a possibility that those who are not willing to accept a new idea or new PS concepts in particular will not even be bothered to answer the questionnaire or participate in the study at all. Therefore, we will not be able to identify/synthesis what causes them to deny this new concept. This will be a limitation of this study and will be acknowledged in limitations. L682-684<br><br>.
Inclusion and exclusion criteria - Please mention clearly.	Added in Study Design.	L385-394

Qualitative component - How do you know sample size of 12 - 14 will come to the saturation point?	We agree that it has to be changed to "semi-structured interviews will be conducted with nursing educators until data saturation is reached".	L443-444
The sampling method should be more precise and practical for each study component. Eg. How you are going to recruit 105 from each institution mentioned/ how many from each/ sub sampling mechanisms.....	Included subsampling methods in sample calculation L410	L 388- all available curricula in selected institutions will be included in content verification using a purposive sampling technique<br>L 396-The participants will be selected from three types of institutions (state universities, private universities, and training schools) using stratified random sampling.<br>L400- participants who were selected using stratified random sampling.<br>L431- Participants will be selected purposive sampling method.
Validation is planned to be done on Hurt-Joseph-Cook individual innovativeness questionnaire. Pls clearly mention what is the validating procedure? Is it construct validity? If so is it factor analysis? EFA? CFA? Both? Then what are the steps? Is it PCA?	EFA and CFA. The steps of validating procedure is mentioned in "Data analysis" Phase 1. L552-L556.	Extraction Method: Principal Component Analysis.<br>Rotation Method: Varimax with Kaiser Normalization.
But SoC questionnaire is already validated in Sri Lanka with good psychometric properties. Then	It is an attempt follow a good practice since wording of the questionnaire was changed to suit	

why you are going to perform CFA on it?	patient safety education context. "Subsequent validation" is suggested by Tsang et al. (2017)	
How to ensure the validity and reliability of self-administered questionnaire which will be used in study in addition to two standard questionnaires?	The self-administered questionnaire has 3 parts.<br>Part1- Demographics- standard questions are being used.<br>Part2-SoC questionnaire- cronbatch alpha, subsequent validation CFA<br>Part3-II questionnaire- cronbatch alpha, validation using EFA and CFA	
As mentioned in the supporting materials, scoring of the Individual Innovation scale, > 68 = high; < 64 = low. Then what about the score between 64 - 68?	Thank you for pointing this out. We accept that this information is incomplete. Unfortunately, there is no description for the score between 68-64 in the scale. We would like to remove this part as the scoring above this description provides more clarity on innovativeness categories.	
Anonymity (which is fundamental in obtaining views clearly and successful manner) could be secured if you are going to use a coding system.	Thank you for your valuble suggestion. It is mentioned in the protocol in "Data Collection"	L481-482 Data collection is not anonymised. In order to protect privacy and confidentiality; names and university affiliations will be replaced with codes during data processing.

References

Danermark B, Ekström M, Karlsson JC. Explaining society: Critical realism in the social sciences. Routledge; 2019 Mar 20.

Saxena D. Guidelines for conducting a critical realist case study. International Journal of Adult Education and Technology (IJAET). 2021 Apr 1;12(2):18-30.

Tsang S, Royse CF, Terkawi AS. Guidelines for developing, translating, and validating a questionnaire in perioperative and pain medicine. Saudi journal of anaesthesia. 2017 May 1;11(Suppl 1):S80-9.

Wynn Jr DE, Williams CK. Critical realm-based explanatory case study research in information systems.
