# Supplementary material for: A critical realist analysis of nursing educators’ willingness to learn and teach patient safety in Sri Lanka: Study protocol
Source: PLoS One. 2025 May 19;20(5):e0323561. doi: 10.1371/journal.pone.0323561 (PMC12088512; doi:10.1371/journal.pone.0323561)
Supplement: Attachment — Submitted filename: PONE-D-23-13398_Review_PY.docx [file pone.0323561.s005.docx]

P – Page and L- Line

**Reviewer Feedback**

**ABSTRACT**

I. P1 L16. College of Nursing or NTS
II. P1 L21. DAARCC - What is this abbreviation means?
III. What will happen in phase-04? What is the rationale for keeping a phase for data analysis?
IV. P2 L9-10. What method will be used to analyse qualitative data?

**What is already 1 known about the topic?**

V. P3 L4. Briefly mention those seven categories or write it in a different way
VI. P3 L9. factors which affects or factors which from
VII. P3 L11. possibility of synergising

I**ntroduction**

VIII. P4 L21 Define an abbreviation when using the first time
IX. P5 L10-11 Reverse the sentence to avoid confusion – …Patient safety concept must be integrated into the curriculum for producing competent nurses. (This is just an idea)
X. P5 L14 ‘stages of concern’ – Please define the new concept when using the first time in the manuscript.
XI. P6 L1-3 – Reverse the sentence (Look at the comment for P5 L10-11).
XII. P6 L7-9 –Please rewrite. Quite confusing
XIII. P7 L1-7 – Include more information about the patient safety curriculum – Especially curriculum content
XIV. P7 L14 - becoming competent using it – this should correct as ‘becoming competent in using it’ or ‘becoming a competent user’
XV. P7 L16 – Sate reference for your claim
XVI. P8 L5-6 – Rewrite the sentence. Eg. …innovation highly depends on personal choice or innovation is highly influenced by intrinsic factors of a faculty.
XVII. P9 L6 - Sate reference for your claim
XVIII. P9 L8 - Sate reference for your claim
XIX. P9 L11 – follow a referencing style - Hurt et al (1997) or Hurt et al (33)?
XX. P9 L12 – Replace (ii) by as
XXI. P9 L19 – Replace involves by includes or incorporates
XXII. P10 L4 – Relate how the educational environment influences adoption
XXIII. P11 L3 – Critical
XXIV. P11 L4 - Replace – ‘is more an’	as	‘is more towards’

**Aim of the study**

XXV. P12 L14 – Aim should be SMART – don’t include details about methods in the aim
XXVI. P14 L3 - education system or nursing educators?
XXVII. P14 L5 - local nursing curricula in Sri Lanka
XXVIII. P14 L7 – integrators or developers?
XXIX. P14 L14-16 – Repetition | It is not necessary
XXX. P14 L22 - Sate reference – ‘extensive methods and intensive methods’

**Sampling Technique**

XXXI. P16 L13 – Are you excluding Tamil-speaking academics? If so, provide a rationale
XXXII. P16 L14 – Why can’t you include faculty who teach basic sciences to nursing students?
XXXIII. P19 L20-21 – How will you follow up? Also, please mention that this is not an anonymous data collection

**Phase 3- Intensive/ qualitative procedures (P19)**

XXXIV. P20 L1. Semi-structured interviews - **Please attach an interview guide (including a pre-determined set of open questions and interview ground rules)** in this section**.**

- Where will you conduct the interview?
- Which language will be used?
- Recording and transcribing process and its confidentiality

**Data Analysis:**

XXXV. P20 L16. Curriculum content verification- **Elaborate more about how will you calculate curricular contents’ coverage percentages**
XXXVI. P21 L15. **Follow scientific writing style** - Thematic analysis will be used to analyse….
XXXVII. P21 L16. Describe more about **how you will conduct thematic analysis (Inductive or reductive?).**
XXXVIII. P21 L21 ‘Validity and reliability of the adapted SoCQ Sinhala version will be assessed’ **you mentioned that the SoCQ Sinhala version has already been validated in Sri Lanka. Why are you attempting to validate again?**
XXXIX. Also, **please elaborate on the steps and analysis that you intended to use in the psychometric study**

**Phase 4: Interpretation phase:**

XL. P22 L2. ‘quantitative and qualitative data will be collected and triangulated’ **explain more about the triangulation process.**

**Discussion**

XLI. P24 L10-12. **Provide references for your claim**
XLII. P24 L18. Sri Lankan **nursing** education context
XLIII. P24 L23. **State the three domains of reality**
XLIV. P25 L13. **BMC Nursing journal or PLoS One**

**General Comment**

1. Please clarify this,

- As far as I understood, Sri Lankan nursing educators are very competent in English. Also, the medium of instruction is English in most Sri Lankan nursing institutes. In this context, what is necessary to translate the questionnaire into a local language?
- If done so, **how can you generalize your result in Sri Lanka** since there are two official languages (Sinhala and Tamil)?

2. Please emphasise in the manuscript that you will use two sets of samples separately for the Psychometric study and questionnaire survey.
3. Follow the same style of citation throughout the manuscript. Also, use correct form of citation (full stop follows after bracket – See P6 L2 and P6 L9).
