# Supplementary material for: A critical realist analysis of nursing educators’ willingness to learn and teach patient safety in Sri Lanka: Study protocol
Source: PLoS One. 2025 May 19;20(5):e0323561. doi: 10.1371/journal.pone.0323561 (PMC12088512; doi:10.1371/journal.pone.0323561)
Supplement: Attachment — Submitted filename: Response to Reviewers.docx [file pone.0323561.s008.docx]

Responses to Editor’s and reviewers’ comments

Dear Editor and reviewers,

Thank you for reviewing our manuscript. We highly appreciate the time and effort you have made to point out the amendments needed. We have tried our best to provide sufficient rationale to each of our decisions. Please find below, the responses and details of amendments done to the manuscript.

| **Reviewer / editor comments** | **Explanations** | **Amendments done** (Location in manuscript- without tracked changes) |
| --- | --- | --- |
| **Editor** |  |  |
| 1. Please ensure that your manuscript meets PLOS ONE's style requirements, including those for file naming. The PLOS ONE style templates can be found at  https://journals.plos.org/plosone/s/file?id=wjVg/PLOSOne_formatting_sample_main_body.pdf and  https://journals.plos.org/plosone/s/file?id=ba62/PLOSOne_formatting_sample_title_authors_affiliations.pdf |  | Style updated. |
| 2. Your ethics statement should only appear in the Methods section of your manuscript. If your ethics statement is written in any section besides the Methods, please delete it from any other section. |  | Moved to methods section. L 563 |
| 3. Please include a separate caption for each figure in your manuscript. |  | Included. L 275, L 337 |
|  |  |  |
| 4. Have the authors described where all data underlying the findings will be made available when the study is complete? | Data will be made available once the study is complete. At this stage researcher does not have any data to be shared. | Availability of data and materials –Data will be available upon request when the study is completed and published. L9-10 |
|  |  |  |
| Reviewer 1 |  |  |
| The authors have given a thorough description of the study background, the study objectives and purposes, and the intended methodology.  While the protocol is well written, I would like to see **more discussion on the use of critical realism as the methodology.** From what I could gather, the protocol described a mixed methods approach, and it is **unclear about the justification/demonstration of the use of critical realism as the research method.** | Thank you for your positive feedback.  We included the definition of “methodology” in use: “Methodology is concerned with the theoretical and philosophical assumptions about methods.(1) It provides the frame of reference for the research which is influenced by the paradigm in which our theoretical perspective is placed or developed…. Methodology can be objectivist, subjectivist or critical realist.”  Methodology- Critical realism  Data collection methods- mixed method | L 212-L 218 |
|  |  |  |
| Furthermore, it would be useful for the authors to clearly define the limitations of other empirical approaches (p.11, line 18), in order to introduce critical realism. | In this study empirical evidence will be obtained on the stages of concerns, in individual, environmental and innovation related areas, and critical realism’s depth reality will be used to understand complexities that are not obvious and visible. L 272-274 | Limitations of objective and subjective methodologies added. L219-L250 |
|  |  |  |
| It would have been useful for the authors to describe their hypotheses (at least for the quantitative study). |  | Included in L 292-296 |
|  |  |  |
| Have the study instruments been validated for their use in Sri Lanka or South East Asia? It’d be great to include some validation evidence in the study protocol. |  | Included in.  SoCQ validation evidence L425-L433.  II scale L 442-443 |
|  |  |  |
| It would have been critical for the authors to justify the sample size and sampling strategy. Has a power analysis been conducted to ensure the sample is adequate? | Sampling strategy was altered post reviewer comments. New calculation included. Total population (updated) =413 Tamil speaking educators= 35 (approx) Sinhala fluent educators = 378 σ = √(p(1-p)) = 0.5. α = 1 - 0.95 = 0.05. p = 1 - \| α \| = 1 - \| 0.05 \| = 0.975; \| 2 \| \| 2 \| n = \| Z^2^_0.975_ * p(1 - p); \| MOE^2^ n = \| 1.96^2^ * 0.5(1 - 0.5) \| = 384.1459; \| 0.05^2^ \| Rounded up to: 385. Since the population size is finite: N=278, the corrected sample size is: n' = \| n * N; \| n + N - 1 n' = \| 384.1459 * 278 \| = **161.5265**; \| 384.1459 + 278 - 1 \| Rounded up to: **162**. Using Yamane's formula with a population size of 273 and a margin of error of 5%, sample size for 2nd sample can be calculated as follows: n = N / (1 + N(e)*2) n = 273 / (1 + 273(0.05) *2) n = 273 / (1 + 273(0.0025)) n = 273 / (1 + 0.6825) n = 273 / 1.6825 n ≈ 162.29 n=162 + non respondents 5% = 171 | Updated: L 401-405  Using Yamane's formula (2,3) n = N / (1 + N(e)^2), for a population of 273 with margin of error of 5%, sample is calculated to be 162. Considering factor analysis for SoC questionnaire (35 items; 1 item: 5 educators), 175 educators are required to calculate CFA. A total of 185 results after adding 5% as non-respondents. The higher value, 185 will be taken as the appropriate sample size for sample 2 |
|  |  |  |
| In the abstract under study design, it would have been useful to describe the study design in a sequential order (i.e., Phase 1, Phase 2, Phase 3, and Phase 4). | Corrected. Data collection, analysis and interpretation will take place in four consecutive phases; phase 1, linking phase, phase 2 and interpretation phase…. | L 29-38 |
|  |  |  |
| **Reviewer 2** |  |  |
| General Comment  1. Please clarify this, • As far as I understood, Sri Lankan nursing educators are very competent in English. Also, the medium of instruction is English in most Sri Lankan nursing institutes. In this context, what is necessary to translate the questionnaire into a local language? | We opted for a translated instrument due to following reasons:   1. Using a validated tool for a particular population is mandatory an academic research. As Gjersing et al., explain (4)"However, a previously validated instrument does not necessarily mean it is valid in another time, **culture or context**." 2. If a study is conducted in English language, in a country where the population is non-native English speakers, it could hamper the quality of data. There is a possibility that level of English proficiency of the participant affects the quality, nuances and the richness of responses they provide. In a self-administered questionnaire, it could affect the way they perceive questions and their intensions. 3. Researchers have emphasized the necessity of translation to a local language of the intended population and cultural adaptation of research questionnaires/ tools when validating.(5–7) |  |
|  |  |  |
| If done so, how can you generalize your result in Sri Lanka since there are two official languages (Sinhala and Tamil)? | Results will not be able generalize in Sri Lanka. Study results will only be generalized among the Sri Lankan nursing educators who are fluent in Sinhalese Thank you for highlighting this. It will be specified as a limitation. | Updated in Discussion. L 601 |
|  |  |  |
| 2. Please emphasise in the manuscript that you will use two sets of samples separately for the Psychometric study and questionnaire survey. | For the self-administered questionnaire, two sets (sample 1 and sample 2) of nursing academics will be recruited. Data from sample 2 will be used to questionnaire survey analysis | L 379-382 |
|  |  |  |
| 3. Follow the same style of citation throughout the manuscript. Also, use correct form of citation (full stop follows after bracket – See P6 L2 and P6 L9). |  | Corrected. |
|  |  |  |
|  |  |  |
| **ABSTRACT** |  |  |
| I.          P1 L16. College of Nursing or NTS | College of Nursing is also a commonly used term. Nurses Training School was used as it is the term used in official documents by the Ministry of Health (NTS -Nurses Training School) 1. latest available Annual health bulletin produced by Ministry of Health, Sri Lanka- 2020, page XXV.  2.http://www.health.gov.lk/moh_final/english/others.php?pid=99 |  |
| II.          P1 L21. DAARCC - What is this abbreviation means? | D- description  A -analytical resolution  A- abduction/theoretical redescription  R- retroduction  C-comparison between different theories and abstractions  C-concretization and contextualization  Used in CRMMSED.(8) | Removed abbreviation as it deemed inappropriate for the abstract. Changed the text to "The stages in an explanatory research based on critical realism and Mixed method.." |
| III.          What will happen in phase-04? | Wording of phases corrected according to CRMMSED.    It is the interpretation phase where we will compare/triangulate quantitative data analysis and qualitative data analysis.(9)    The aim is to identify how individual, environmental and patient safety education content bring about different stages of concern in educators.  Further, aligning our analysis with the critical realistic depth reality, we will employ critical realist causal analysis techniques (10)to identify events structures and mechanisms that contribute to each stage of concern. |  |
| III. What is the rationale for keeping a phase for data analysis? | Corrected, CRMMSED phases will be used throughout. Initially there was a mixed up with original MMSE design. | In phase 2, qualitative data will be collected through semi-structured interviews with nursing educators and thematically analysed. L35-36 |
| IV.          P2 L9-10. What method will be used to analyse qualitative data? | Thematic analysis will be used. | In phase 2, qualitative data will be collected through semi-structured interviews with nursing educators and thematically analysed. L35-36 |
|  |  |  |
| **What is already 1 known about the topic?** |  |  |
| V.          P3 L4. Briefly mention those seven categories or write it in a different way |  | added: " ranging from “unconcerned” to “informational”, “personal”, “management”, “consequence” “collaboration” and “refocusing”. " L 57-59 |
| VI.          P3 L9. factors which affects or factors which from |  | Corrected; innovation-related factors which form the concerns of nursing educators, L 63 |
| VII.          P3 L11. possibility of synergising |  | Corrected L65 |
|  |  |  |
| I**ntroduction** |  |  |
| VIII.          P4 L21 Define an abbreviation when using the first time |  | added abbreviation at L75, and changed throughout the manuscript. |
| IX.          P5 L10-11 Reverse the sentence to avoid confusion – …Patient safety concept must be integrated into the curriculum for producing competent nurses. (This is just an idea) |  | Corrected. Nursing educators must be willing to integrate and teach new PS concepts to produce nurses who can champion PS. L 102-103 |
| X.          P5 L14 ‘stages of concern’ – Please define the new concept when using the first time in the manuscript. | removed stages of concern as it was identified as too early to mention. It will be explained in details under Concerns Based Adoption Model (CBAM) | Changed to “Therefore, further research is needed to evaluate both the curricula and nurse educators' concerns on teaching these concepts. L 102-105 |
| XI.          P6 L1-3 – Reverse the sentence (Look at the comment for P5 L10-11). |  | rewrote: The lack of resources is the main barrier to promoting patient safety and quality practices in India. L 11-112 |
| XII.          P6 L7-9 –Please rewrite. Quite confusing |  | rewrote: A study done in a teaching hospital in Sri Lanka revealed that nursing officers who are competent in skills regard to adverse events and, near misses were 6.35%. L 116-118 |
| XIII.          P7 L1-7 – Include more information about the patient safety curriculum – Especially curriculum content |  | Content included: L130-136 |
| XIV.          P7 L14 - becoming competent using it – this should correct as ‘becoming competent in using it’ or ‘becoming a competent user’ |  | Changed: becoming competent **in** using it. L149 |
| XV.          P7 L16 – Sate reference for your claim |  | A 35-item Stages of Concern questionnaire is available in order to determine where someone is in the Stages of Concern range.(11) L 155-156 |
| XVI.          P8 L5-6 – Rewrite the sentence. Eg. …innovation highly depends on personal choice or innovation is highly influenced by intrinsic factors of a faculty. |  | changed to: it is important to note that acceptance and adoption of an innovation is highly influenced by intrinsic factors of an individual. L 157-158 |
| XVII.          P9 L6 - Sate reference for your claim |  | Roger's diffusion of innovation theory. Updated L 175 |
| XVIII.          P9 L8 - Sate reference for your claim |  | Roger's diffusion of innovation theory L 178 |
| XIX.          P9 L11 – follow a referencing style - Hurt et al (1997) or Hurt et al (33)? |  | Corrected. Hurt et al., (34)L 181 |
| XX.          P9 L12 – Replace (ii) by as |  | Hurt et al (1997) define Individual Innovativeness (II) as the “willingness of an individual to change”.L181 |
| XXI.          P9 L19 – Replace involves by **includes** or incorporates |  | changed to: includes L189 |
| XXII.          P10 L4 – Relate how the educational environment influences adoption | Research done in the management and IT industries has found that positive working environment such as strong managerial support, allocation of sufficient resources and incentives become crucial catalysts in embracing innovation. As such, it could be predicted that nursing educators' environment could affect education innovation adoption. | Updated L 196-199 |
| XXIII.          P11 L3 – Critical |  | Corrected. L 258 |
| XXIV.          P11 L4 - Replace – ‘is more an’ as ‘is more towards’ | We wanted to emphasize was that by nature, CR is essentially an ontology, not that it has more tendency to be an ontology. |  |
|  |  |  |
| **Aim of the study** |  |  |
| XXV.          P12 L14 – Aim should be SMART – don’t include details about methods in the aim |  | Corrected L 290- 291 |
| XXVI.          P14 L3 - education system or nursing educators? |  | Changed to Sri Lankan nursing educators. L 324 |
| XXVII.          P14 L5 - local nursing curricula in Sri Lanka |  | Corrected L 325 |
| XXVIII.          P14 L7 – integrators or developers? |  | Developers L 328 |
| XXIX.          P14 L14-16 – Repetition \| It is not necessary |  | Removed. |
| XXX.          P14 L22 - State reference – ‘extensive methods and intensive methods’ |  | Updated L 343 |
|  |  |  |
| **Sampling Technique** |  |  |
| XXXI.          P16 L13 – Are you excluding Tamil-speaking academics? If so, provide a rationale | 1. Unavailability of a study sample (number of Tamil speaking educators) to conduct a validation study.  2. Time limitation.  We would like to emphasize that we have no intention of excluding Tamil speaking educators or create any racial discrimination. If they recognise themselves as fluent in Sinhala as the secondary or tertiary language, they will be included in the sample. This will be mentioned as a limitation of the study. | Permanent nursing educators (N total=413) who are capable of reading, speaking and comprehending Sinhala language (NS≈378)  L 371-372 |
| XXXII.          P16 L14 – Why can’t you include faculty who teach basic sciences to nursing students? | 1. It is very unlikely that adverse events related patient safety content will be taught in basic sciences (anatomy, physiology and biochemistry).  2. The study aims to analyse perceptions of nursing educators who teach core nursing subjects and who can influence/ contribute to the improvement of core nursing curricula content. | teach at-least one nursing subject (clinical nursing subjects, fundamentals of nursing, management in nursing, trends and issues in nursing etc) whom will have the potential to provide feedback on nursing curriculum, will be selected for the sample. L374-5 |
| XXXIII.          P19 L20-21 – How will you follow up?   Also, please mention that this is not an anonymous data collection |  | Non-respondents will receive a reminder email on following week and a phone reminder on the 2nd week. L 483  added in phrase  "Data collection is not anonymized. To protect privacy and confidentiality, names and university affiliations will be replaced with codes during data processing. Any data requested during publications will also be anonymized." L 457-459 |
|  |  |  |
|  |  |  |
| **Phase 3- Intensive/ qualitative procedures (P19)** |  |  |
| XXXIV.          P20 L1. Semi-structured interviews - **Please attach an interview guide (including a pre-determined set of open questions and interview ground rules)** in this section**.** |  | Attached. S1File |
| ·        Where will you conduct the interview? |  | changed to: one to one interviews will be conducted in-person or virtually according to participant’s preference. If done in-person, it will be at the participants' academic institution. L 489- L 491 |
| ·        Which language will be used? |  | Interview will be conducted in participants preferred language (Sinhala, Tamil or English). A Tamil-Sinhala-English translator will be available during the interview for assistance upon permission from the participant. L 493-5 |
| ·        Recording and transcribing process and its confidentiality |  | Updated. L 495-L 501 |
|  |  |  |
| **Data Analysis:** |  |  |
| XXXV.          P20 L16. Curriculum content verification- **Elaborate more about how will you calculate curricular contents’ coverage percentages** | percentages will be calculated on the inclusion of five selected concepts in each curriculum. The selected adverse events related patient safety concepts are, 1) definition of adverse events (AE) in healthcare, 2) Adverse event reporting, 3) human factors in healthcare safety, 4) system factors in healthcare safety and 5) root cause analysis (RCA).  Students knowledge outcomes mentioned in WHO-MPSC relevant to above concepts (total 11) and introduction to incident reporting form provided by Directorate of Healthcare Quality and Safety, Sri Lanka will be checked against local curricula of each participating institution (S2 File). | L 511-521 |
| XXXVI.          P21 L15. **Follow scientific writing style** - Thematic analysis will be used to analyse…. |  | Updated 545-551 |
| XXXVII.          P21 L16. Describe more about **how you will conduct thematic analysis (Inductive or reductive?).** | Deductive thematic analysis will be conducted, based on the conceptual framework presented. Yet, the researchers will not get limited by the framework and will also include inductive analysis, given that new constructs could appear during the analysis phase. | L 546-548 |
| XXXVIII.          P20 L21 ‘Validity and reliability of the adapted SoCQ Sinhala version will be assessed’ **you mentioned that the SoCQ Sinhala version has already been validated in Sri Lanka. Why are you attempting to validate again?** | It was an attempt follow a good practice since wording of the questionnaire was changed to suit patient safety education context. "Subsequent validation" is suggested by Tsang et al.(7) |  |
| XXXIX.          Also, **please elaborate on the steps and analysis that you intended to use in the psychometric study** |  | Updated L 524-531 |
|  |  |  |
| **Phase 4: Interpretation phase:** |  |  |
| XL.          P22 L2. ‘quantitative and qualitative data will be collected and triangulated’ **explain more about the triangulation process.** |  | Updated L 554-561 |
|  |  |  |
| **Discussion** |  |  |
| XLI.          P24 L10-12. **Provide references for your claim** |  | Included L 583 |
| XLII.          P24 L18. Sri Lankan **nursing** education context |  | Updated: While the primary focus is on Sri Lanka, the study's insights and methodological approach have broader applicability to similar studies worldwide. By understanding the underlying mechanisms shaping **nursing education** in Sri Lanka, the research aims to provide context-specific suggestions, while also contributing to the broader knowledge base in education research. L 591 |
| XLIII.          P24 L23. **State the three domains of reality** |  | Added in. L 595 |
| XLIV.          P25 L13. **BMC Nursing journal or PLoS One** |  | Corrected. |

References

1.	Uyangoda J. Writing research proposals in the sosial sciences and humanities : a theoritical and practical guide [Internet]. Social Scientists’ Association; 2010 [cited 2022 Sep 30]. 135 p. Available from: https://books.google.com/books/about/Writing_Research_Proposals_in_the_Sosial.html?id=ko7cjwEACAAJ

2.	Yamane T. Statistics: an introductory analysis. 3rd ed. New York: Harper and Row; 1973. 1130 p.

3.	Israel GD. Determining sample size [Internet]. 1992 [cited 2023 Jul 18]. Available from: https://www.researchgate.net/profile/Subhash-Basu-3/post/how_could_i_determine_sample_size_for_my_study/attachment/5ebaa4924f9a520001e613b6/AS:890361492811785@1589290130539/download/samplesize1.pdf

4.	Gjersing L, Caplehorn JR, Clausen T. Cross-cultural adaptation of research instruments: Language, setting, time and statistical considerations. BMC Med Res Methodol. 2010;10.

5.	Guillemin F, Bombardier C, Beaton D. Cross-cultural adaptation of health-related quality of life measures: Literature review and proposed guidelines. J Clin Epidemiol [Internet]. 1993 Dec 1 [cited 2023 Jul 17];46(12):1417–32. Available from: http://www.jclinepi.com/article/089543569390142N/fulltext

6.	Beaton DE, Bombardier C, Guillemin F, Ferraz MB. Guidelines for the process of cross-cultural adaptation of self-report measures. Spine (Phila Pa 1976) [Internet]. 2000 [cited 2022 Oct 1];25(24):3186–91. Available from: https://journals.lww.com/spinejournal/Fulltext/2000/12150/Guidelines_for_the_Process_of_Cross_Cultural.14.aspx

7.	Tsang S, Royse CF, Terkawi AS. Guidelines for developing, translating, and validating a questionnaire in perioperative and pain medicine [Internet]. Vol. 11, Saudi Journal of Anaesthesia. 2017 [cited 2022 Oct 1]. p. S80–9. Available from: https://www.saudija.org/article.asp?issn=1658-354X;year=2017;volume=11;issue=5;spage=80;epage=89;aulast=Tsang

8.	Gail Baisley H. Pedagogies of presence ; contemplative education across the disciplines in Aotearoa New Zealand [Internet]. Massey University; 2020 [cited 2022 Oct 1]. Available from: https://mro.massey.ac.nz/handle/10179/15782

9.	Tonkin-Crine S, Anthierens S, Hood K, Yardley L, Cals JWL, Francis NA, et al. Discrepancies between qualitative and quantitative evaluation of randomised controlled trial results: Achieving clarity through mixed methods triangulation. Implement Sci [Internet]. 2016;11(1):1–8. Available from: http://dx.doi.org/10.1186/s13012-016-0436-0

10.	Sayer A. Method in Social Science, A realist approach [Internet]. 2nd ed. London and New York: Routledge; 2010. 336 p. Available from: https://www.taylorfrancis.com/books/mono/10.4324/9780203850374/method-social-science-andrew-sayer

11.	George AA, Hall GE, Stiegelbauer SM, Southwest Educational Development Laboratory. Measuring implementation in schools : the stages of concern questionnaire [Internet]. Austin, TX: SEDL. Available from; 2006. 98 p. Available from: http://www.sedl.org/pubs/catalog/items/cbam17.html

12.	Hurt HT, Joseph K, Cook CD. Scales for the measurement of innovativeness. Hum Commun Res. 1977;4(1):58–65.
