# Supplementary material for: A critical realist analysis of nursing educators’ willingness to learn and teach patient safety in Sri Lanka: Study protocol
Source: PLoS One. 2025 May 19;20(5):e0323561. doi: 10.1371/journal.pone.0323561 (PMC12088512; doi:10.1371/journal.pone.0323561)
Supplement: Attachment — Submitted filename: R5_DrPY.docx [file pone.0323561.s007.docx]

Overall, this is a good study!

Referencing: Does the in-text citation come after a full stop | .(1) | or before a full stop | (1). |

Abstract

L36 –Is it inductive or reductive thematic analysis?

What is already 1 known about the topic?

L63- Please carefully read the previous comment: ….factors which from

Introduction

L181 - Hurt et al (1997) define Individual Innovativeness (II) ….. Here, is the (II) necessary?

Suggestion: Hurt et al., (1997) define Individual Innovativeness as the “willingness of an individual to change”.
