# Supplementary material for: A critical realist analysis of nursing educators’ willingness to learn and teach patient safety in Sri Lanka: Study protocol
Source: PLoS One. 2025 May 19;20(5):e0323561. doi: 10.1371/journal.pone.0323561 (PMC12088512; doi:10.1371/journal.pone.0323561)
Supplement: Attachment — Submitted filename: Response to Reviewers R2.docx [file pone.0323561.s009.docx]

Responses to reviewers’ comments

Dear Editor and reviewers,

Thank you for reviewing our manuscript yet again. We highly appreciate the time and effort you have made to point out the amendments needed. We have tried our best to provide sufficient rationale to each of our decisions. Please find below, the responses and details of amendments done to the manuscript.

| **Reviewer comments** | **Explanation** | **Amendments done (Location in manuscript)** |
| --- | --- | --- |
|  |  |  |
| Reviewer #2: Overall, this is a good study! | Thank you very much for your positive feedback. It encourages us to strive and do better. |  |
|  |  |  |
| **Referencing:** |  |  |
| Does the in-text citation come after a full stop \| .(1) \| or before a full stop \| (1). \| | We were using Vancouver Referencing Style as directed in journals' author guidelines which instructs to have citations number after full-stop.. But we observed the published articles in the journal has in text citations after full-stop. We have changed accordingly. Thank you. | In text citations |
|  |  |  |
| **Abstract** |  |  |
| L36 –Is it inductive or reductive thematic analysis? | Deductive thematic analysis will be conducted, based on the conceptual framework presented . Yet, the researchers will not get limited by the framework and will also include inductive analysis, given that new constructs could appear during the analysis phase.(L 683-685). We only mentioned as thematic analysis in the abstract due to word limitation. |  |
|  |  |  |
| **What is already 1 known about the topic?** |  |  |
| L63- Please carefully read the previous comment: ….factors which from | Thank you for highlighting the error and your suggestion. We wanted to identify the factors which contribute to the emergence of different concerns through a critical realist perspective. We have decided to change it as "**factors which contribute to the emergence of different concerns".** For your consideration please. | L56 |
|  |  |  |
| **Introduction** |  |  |
| L181 - Hurt et al (1997) define Individual Innovativeness (II) ….. Here, is the (II) necessary?Suggestion: Hurt et al., (1997) define Individual Innovativeness as the “willingness of an individual to change”. | Thank you, changed as suggested. | L 175 |
|  |  |  |
|  |  |  |
|  |  |  |
|  |  |  |
| Reviewer #3: |  |  |
|  |  |  |
| 1. The abstract should adhere to the prescribed word limit. It is imperative to eliminate extraneous elements, such as ethical considerations, from the abstract. | Thank you for highlighting this. We have removed ethical considerations from abstract. |  |
|  |  |  |
| 2. In the context of scholarly writing, it is customary to place citations within brackets at the end of a sentence, with the full stop occurring after the closing bracket. | We were using Vancouver Referencing Style as directed in journals' author guidelines which instructs to have citations number after full-stop. But we observed the published articles in the journal has in text citations after full-stop. We have changed accordingly. Thank you. |  |
|  |  |  |
| 3. The term "programme" should be spelled with the correct spelling, not as "program. | Noted with thanks. Corrected. | L 80, L 328, |
|  |  |  |
| 4. Avoid commencing a sentence with a numeral. | Corrected, thanks. | L 111- Nearly half 46% |
|  |  |  |
| 5. Definitions of phrases or concepts should be shortened while ensuring that the meaning remains intact | Corrected, thanks. | L 175-The general innovativeness of an individual is typically viewed as a psychological construct or individual characteristic. It shapes an individual’s disposition toward newness regardless of the kind of innovation (33) L 186- Structural empowerment refers to workplace conditions and policies that facilitate four dimensions. These dimensions are access to information, support.... |
|  |  |  |
| 6. Avoid the use of abbreviations in the objectives. | noted with thanks | L 327- Patient safety L 333, L 337, L339 |
|  |  |  |
| 7. The "significance of the study" and "conceptual framework" components should precede the objectives. | noted with thanks | Changed |
|  |  |  |
| 8. The methodologies that will be employed in the study should be delineated under the section dedicated to study methodology, rather than within the introduction. | The article format suggests Introduction, materials and methods, discussion. But I cannot include methodology under methods as I discuss theoretical foundations under this section. It is more suitable to be in Introduction section rather than methods. So I added in a Methodology sub section in Introduction. For your consideration please | L 207 |
|  |  |  |
| 9. Whenever referring to the Ministry of Health, use the complete designation "Ministry of Health, Sri Lanka. | Thanks, changes were made. | L 24, L 373, L 474 |
|  |  |  |
| 10. If data collection is not anonymous, it is imperative to provide a more detailed explanation, particularly focusing on aspects such as confidentiality, data quality, and ethical considerations. | Thank you for highlighting this. We have included details of ethical considerations. | L 573-625 |
|  |  |  |
| 11. In the write-up, use the future tense appropriately to convey actions or events that will occur or be completed in the future. | Corrected, thanks. | L 309- this study will be useful for the future  L 55, L58 L421- will be ascertained |
