# Supplementary material for: A critical realist analysis of nursing educators’ willingness to learn and teach patient safety in Sri Lanka: Study protocol
Source: PLoS One. 2025 May 19;20(5):e0323561. doi: 10.1371/journal.pone.0323561 (PMC12088512; doi:10.1371/journal.pone.0323561)
Supplement: Attachment — Submitted filename: Responses to reviewers R3.docx [file pone.0323561.s011.docx]

**Responses to reviewers’ comments**

Dear Editor and reviewers,

Thank you for reviewing our manuscript yet again. We highly appreciate the time and effort you have made to point out the amendments needed.

We would like to express our sincere gratitude for allocating your time to review our protocol critically and thoroughly. We re-evaluated our conceptual framework, after reviewers’ comments on our methodology and the need hypotheses. We could not help but make an adjustment in the conceptual framework and research title as the protocol demanded these changes. We have attached the amended conceptual framework.

We had to change the title from “A critical realist analysis of nursing educators’ **concerns on patient safety** education in Sri Lanka: study protocol” to “A critical realist analysis of nursing educators’ **willingness to learn and teach patient safety** in Sri Lanka: study protocol. In the conceptual framework, Stages of Concern deemed more apt to be under Individual factors and it was changed accordingly. We have made these changes identifiable in the manuscript with track changes. For your kind consideration please.

We have tried our best to provide sufficient amendments and justifications when addressing each of your comments. Please find below, the responses and details of amendments done to the manuscript.

| **Reviewer / editor comments** | **Explanation** | **Amendments done (Location in manuscript- without track changes)** |
| --- | --- | --- |
|  |  |  |
| **Reviewer #4:** |  |  |
| Thanks for your valuable article. it seems that the needs for conducting this research should be more explained in the introduction. | Thanks for your encouraging feedback. We have expanded “significance of the study”. | L298-309 |
|  |  |  |
| **Reviewer #5:** |  |  |
| Abstract - It is clearer if authors mention "Methods" after introduction instead of setting and design. Under methods, you could describe all the components (setting, study population, inclusion and exclusion criteria, sample size, sampling method, study instruments....). | Thank you for your suggestion. Changed the subtopics. We did not elaborate on the sampling method, and sampling size in the abstract due to the word limit. But these have been explicitly mentioned in the body of the article. |  |
|  |  |  |
| Under data analysis, what are the statistical tests utilized for analysis? | Factor analysis- EFA and CFA, associations and correlations of questionnaire data were included in the abstract. | L42-44 |
|  |  |  |
| The study protocol is a lengthy one. contain a validation, audit and descriptive studies which involve quantitative and qualitative parts. Better if authors can **clearly mention as stages or phases** under the whole study. | This information is provided in Table 2. Comparison of study designs and in Abstract. | L30-32 L363 |
|  |  |  |
| There are some repetitions which could be avoided and leads to a concise protocol. | We could avoid some repetitions if we rearrange the manuscript according to the Phases (collating sampling, instruments, data collection and analysis together), But then it will deviate from the manuscript guideline of the journal. Nevertheless, minor changes were done to address this. |  |
|  |  |  |
| As this is not an analytical study, how the authors assess the hypothesis mentioned? | Thank you for highlighting this. We agree to your opinion that this study does not necessarily need hypotheses to execute it or for its' analysis. | removed hypotheses. |
|  |  |  |
| In hypothesis 1 - line 341 - repetition of " nursing educators" | removed. | removed. |
|  |  |  |
| Specific objectives 6 and 7 are more or less same. | 6. To examine the perceived attributes and environmental factors that facilitate or hinder the adoption of adverse events related patient safety concepts by nursing educators. 7. To identify the events that contribute to the facilitation or hindrance of the adoption of adverse events related patient safety concepts.  Perceived attributes and environmental factors are in the empirical domain. Phenomena in empirical domain are observed and experienced.  Perceived attributes on an innovative concept cannot be equated to actual events.  We accept that certain "opportunities" participants mention have the possibility of being identified as actual events. To overcome this: if a participant mentions certain opportunity (eg. training) was helpful in accepting new PS concepts, we will use this finding in empirical domain. We will further inquire to find out what directives/policies or recommendations were there enabling these opportunities. Latter findings will be identified as Actual Events. | 7. To identify the **events** that contribute to the facilitation or hindrance of the adoption of adverse events related patient safety concepts. 8. To analyse how variations in willingness to learn and teach PS emerge from the underlying generative mechanisms and structures  To improve clarity we amalgamated the objective on "actual domain” with "real domain" as both objectives are operationalized using causal analysis, retroduction and "synthesis".  **7. To synthesis the actual events, generative mechanisms and structures that contribute to the facilitation or hindrance of the adoption of adverse events related patient safety concepts.** |
|  |  |  |
| Specific objective 8 - Is this realistic? | In Critical realism, retroduction is the central mode of inference. Retroduction is the way to dive beyond the measurable elements of observations through events to identify the structures, context, and mechanisms that **explain the observation.**  Our belief in the existence of a mechanism can be based either on our ability to directly observe it (perceptual criteria), with or without tools to do so, or on our **ability to observe its effects** (causal criteria) (Bhaskar 1975, p. 179).  Retroduction allows researchers to identify a set of plausible candidate causal mechanisms. This enables a researcher to investigate the potential causal mechanisms and the conditions under which certain outcomes will or will not be realised.  Empirical corroboration allows to selection of the mechanism(s) that offers the best explanation (Mukumbang, 2021).These mechanisms belong to different strata of reality: (i) physical, (ii) biological, (iii) psychological, (iv) psychosocial, socio-economic,(v) cultural, and (vii) normative mechanisms (Danermark, 2019). Some examples of such analysis: Saxena, D. (2019), Wynn Jr, D. E., & Williams, C. K. (2008) | 8. To analyse how variations in willingness to learn and teach PS emerge from the underlying generative mechanisms and structures.  **new 7. To synthesis the actual events, generative mechanisms and structures that contribute to the adoption of adverse events related patient safety concepts.**  There is a possibility that those who are not willing to accept a new idea or new PS concepts in particular will not even be bothered to answer the questionnaire or participate in the study at all. Therefore, we will not be able to identify/synthesis what causes them to deny this new concept. This will be a limitation of this study and will be acknowledged in limitations. L682-684   . |
|  |  |  |
| Inclusion and exclusion criteria - Please mention clearly. | Added in Study Design. | L385-394 |
|  |  |  |
| Qualitative component - How do you know sample size of 12 - 14 will come to the saturation point? | We agree that it has to be changed to "semi-structured interviews will be conducted with nursing educators until data saturation is reached". | L443-444 |
|  |  |  |
| The sampling method should be more precise and practical for each study component. Eg. How you are going to recruit 105 from each institution mentioned/ how many from each/ sub sampling mechanisms..... | Included subsampling methods in sample calculation L410 | L 388- all available curricula in selected institutions will be included in content verification using a purposive sampling technique L 396-The participants will be selected from three types of institutions (state universities, private universities, and training schools) using stratified random sampling. L400- participants who were selected using stratified random sampling. L431- Participants will be selected purposive sampling method. |
|  |  |  |
| Validation is planned to be done on Hurt-Joseph-Cook individual innovativeness questionnaire. Pls clearly mention what is the validating procedure? Is it construct validity? If so is it factor analysis? EFA? CFA? Both? Then what are the steps? Is it PCA? ....... | EFA and CFA. The steps of validating procedure is mentioned in "Data analysis" Phase 1. L552- L556. | Extraction Method: Principal Component Analysis. Rotation Method: Varimax with Kaiser Normalization. |
|  |  |  |
| But SoC questionnaire is already validated in Sri Lanka with good psychometric properties. Then why you are going to perform CFA on it? | It is an attempt follow a good practice since wording of the questionnaire was changed to suit patient safety education context. "Subsequent validation" is suggested by Tsang et al. (2017) |  |
|  |  |  |
| How to ensure the validity and reliability of self-administered questionnaire which will be used in study in addition to two standard questionnaires? | The self-administered questionnaire has 3 parts. Part1- Demographics- standard questions are being used. Part2-SoC questionnaire- cronbatch alpha, subsequent validation CFA Part3-II questionnaire- cronbatch alpha, validation using EFA and CFA |  |
|  |  |  |
| As mentioned in the supporting materials, scoring of the Individual Innovation scale, > 68 = high; < 64 = low. Then what about the score between 64 - 68? | Thank you for pointing this out. We accept that this information is incomplete. Unfortunately, there is no description for the score between 68-64 in the scale. We would like to remove this part as the scoring above this description provides more clarity on innovativeness categories. |  |
|  |  |  |
| Anonymity (which is fundamental in obtaining views clearly and successful manner) could be secured if you are going to use a coding system. | Thank you for your valuble suggestion. It is mentioned in the protocol in "Data Collection" | L481-482 Data collection is not anonymised. In order to protect privacy and confidentiality; names and university affiliations will be replaced with codes during data processing. |

References

Danermark B, Ekström M, Karlsson JC. Explaining society: Critical realism in the social sciences. Routledge; 2019 Mar 20.

Saxena D. Guidelines for conducting a critical realist case study. International Journal of Adult Education and Technology (IJAET). 2021 Apr 1;12(2):18-30.

Tsang S, Royse CF, Terkawi AS. Guidelines for developing, translating, and validating a questionnaire in perioperative and pain medicine. Saudi journal of anaesthesia. 2017 May 1;11(Suppl 1):S80-9.

Wynn Jr DE, Williams CK. Critical realm-based explanatory case study research in information systems.
