# Supplementary material for: A critical realist analysis of nursing educators’ willingness to learn and teach patient safety in Sri Lanka: Study protocol
Source: PLoS One. 2025 May 19;20(5):e0323561. doi: 10.1371/journal.pone.0323561 (PMC12088512; doi:10.1371/journal.pone.0323561)
Supplement: Attachment — Submitted filename: Responses to reviewers R4.docx [file pone.0323561.s012.docx]

Responses to reviewers’ comments

Dear Editor and reviewers,

Thank you for reviewing our manuscript yet again. We highly appreciate the time and effort you have made to point out the amendments needed. We have tried our best to provide sufficient rationale to each of our decisions. Please find below, the responses and details of amendments done to the manuscript.

| **Reviewer / editor comments** | **Explanation** | **Amendments done (Location in manuscript- without track changes)** |
| --- | --- | --- |
|  |  |  |
| **Reviewer #5:** |  |  |
|  |  |  |
| The authors have addressed most of the comments to the point. | Thank you. |  |
| The study is interesting however, this study protocol is a lengthy. Better if could concise more. | Thank you for your feedback. We have made some adjustments to the abstract to make it more concise. However, given the broad scope of the study, which covers seven objectives, it is challenging to significantly reduce the content without compromising the clarity and detail necessary to convey the study's comprehensive nature. |  |
| Exclusion criteria should not be the vise versa of the inclusion criteria Eg. In this protocol inclusion criteria will be Sinhala conversant people. Then no need to mention that other than Sinhala conversant people will be excluded from the study. | Thank you for highlighting this. We have amended as Academic staff members who are on maternity leave, overseas study leave and overseas sabbatical leave, due to the due to the potential difficulty in including these individuals in semi-structured interviews during the study period, given their absence from the academic setting. | P19, L 374: Academic staff members who are on maternity leave, overseas study leave and overseas sabbatical leave. |
| In line 686/ 687, it is mentioned that there will be a translator???? If the study will include only Sinhala conversant participants, why the investigators will use a translator? | Thank you for your comment and for raising this important point. While the primary inclusion criterion is that participants should be conversant in Sinhala, we are mindful that there could be variation in language proficiency among the participants. In particular, some Sinhala-speaking participants may also be fluent in Tamil or English (their mother-tongue), and we want to ensure that we accommodate their preferences in terms of the language of the interview. The mention of a Tamil-Sinhala-English translator is to ensure that any potential participants, regardless of their language preferences or proficiency, are provided with the necessary support for clear communication during the interview. The presence of a translator is intended as a precautionary measure and will only be used upon the participant’s permission, ensuring that the process remains flexible and respectful of their language needs. |  |
|  |  |  |
| **Reviewer #6:** |  |  |
|  |  |  |
| The study is generally well written; However, I have suggested some minor changes as per below. | Thank you. |  |
|  |  |  |
| Rather use headings - Background;/Introduction; Methods and analysis and then discussions. Summarize the methods section and in the discussion briefly provide the significance of your study. | Amended | P2, L21-38 |
|  |  |  |
| -The introduction is generally well written with relevant literature. Well done to the authors! | Thank you for your encouraging words. |  |
| Page 6 line 106 Include references of these studies... An assertion such as this one should have been drawn from some existing information. | Included. | P5, L98 |
|  |  |  |
| Page 15 line 297: Suggestion: A summary of this section of your manuscript and the discussion section at the end of your manuscript is what should be included in the discussion section of your **abstract.** | Amended | P2, L31-38 |
|  |  |  |
| Page 16 line 319: These objectives align well with the topic. The study's realist approach ensures deep exploitation of factors, mechanisms and structures influencing nursing educators' willingness to learn and teach patient safety which directly connects to the objectives. | Thank you for validating our approach and confirming the alignment of our objectives with the topic. |  |
|  |  |  |
| Page 17 line 353-355: Include the reference for the statement | Included | P17-347 |
